# Succinate dehydrogenase loss suppresses pyrimidine biosynthesis via succinate-mediated inhibition of aspartate transcarbamylase

**DOI:** 10.1038/s42255-026-01524-w

**Published:** 2026-05-04

**Authors:** Madeleine L. Hart, David Sokolov, Serwah Danquah, Eric Zheng, Alex D. Doan, Kristian Davidsen, David MacPherson, Lucas B. Sullivan

**Affiliations:** 1 Human Biology Division, Fred Hutchinson Cancer Center, Seattle, WA, USA

## Abstract

Decreased availability of the amino acid aspartate constrains cell function across diverse biological contexts, but the temporal interplay between aspartate abundance, downstream metabolic changes and functional effects remains poorly understood. Here we show that succinate dehydrogenase (SDH) inhibition suppresses pyrimidine synthesis via dual effects of cellular aspartate depletion and succinate accumulation. Using an aspartate biosensor and live-cell imaging, we monitor aspartate levels and cell proliferation across several models of aspartate limitation. While complex I inhibition or knockout of aspartate biosynthetic enzymes lead to a strict decrease in aspartate levels and impair proliferation, SDH inhibition produces a unique aspartate rebound, yet fails to restore proliferation. Mechanistically, we find that SDH loss impairs pyrimidine biosynthesis via succinate accumulation, which competitively inhibits aspartate utilization by mammalian aspartate transcarbamylase (ATCase), a key step in pyrimidine biosynthesis. This metabolic interaction occurs in multiple models of SDH deficiency, causing pyrimidine insufficiency, replication stress and sensitivity to ATR kinase inhibition. Taken together, these findings define an unexpected role for succinate in modulating cellular nucleotide homeostasis and demonstrate how cascading metabolic interactions can unfold to impact cell function.

Cellular metabolism is among the most dynamic biological processes, with many biochemical reactions operating at sub-second timescales and metabolite pools turning over on the scale of seconds to hours^1^. Metabolic disruptions can therefore trigger rapid and evolving changes that reverberate across the metabolic network and occur with distinct temporal behaviours. The abundance of the amino acid aspartate is highly responsive to metabolic state, as aspartate is predominantly synthesized by cells via mitochondrial metabolism, after which it serves as a precursor for several major anabolic products. Aspartate limitation—the condition where aspartate levels are insufficient to maximally support cell function—has emerged as a critical functional determinant in many biological contexts^2–21^. However, it remains unclear how aspartate levels change over time during various contexts of aspartate limitation, and how these changes are registered on downstream metabolic fates and cell function. A more nuanced understanding of these factors is important to both better understand the integration of aspartate with metabolic state and to identify opportunities to treat human diseases that interface with aspartate limitation.

## Results

### Temporally resolved measurements of aspartate levels and proliferation reveal nuanced and distinct dynamics across aspartate limitation paradigms

**Fig. 1 |:**
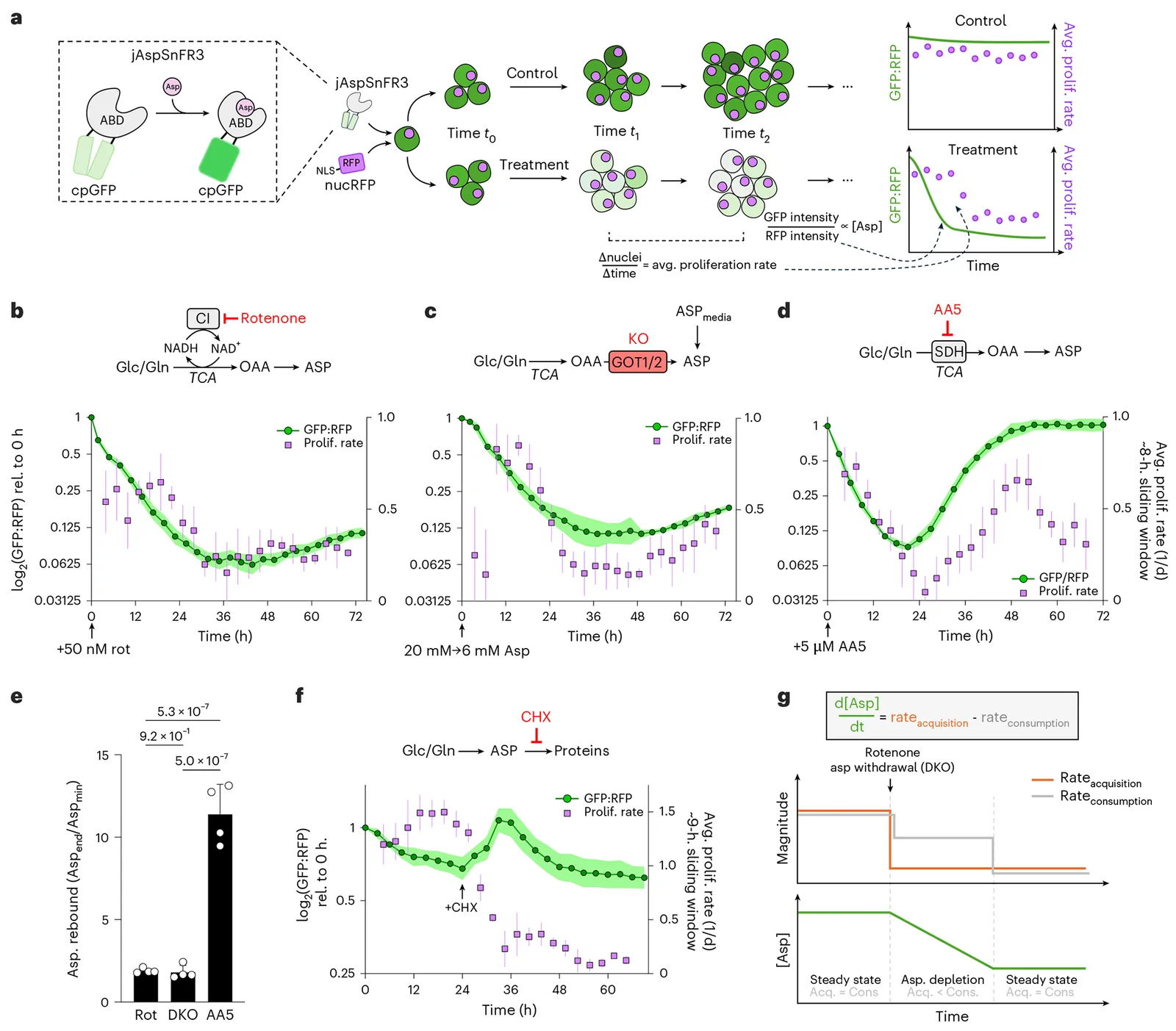
Temporally resolved measurements of aspartate levels and proliferation rate reveal distinct dynamics across aspartate limitation paradigms. **a**, Schematic illustrating the experimental setup allowing simultaneous measurement of relative aspartate levels (jAspSnFR3) and proliferation rates (NucRFP) of cells in culture. GFP:RFP intensities correspond to relative aspartate levels and average (avg.) proliferation rates are calculated using the equation (log_2_(nuclei_*t*2_/nuclei_*t*1_))/(*t*_2_ − *t*_1_) (Methods). **b**, Relative aspartate levels (green line, left *y* axis) and absolute proliferation rates (purple points, right *y* axis) of 143B cells in DMEM without pyruvate treated with the CI inhibitor rotenone (50 nM), which indirectly blocks aspartate synthesis by preventing NAD^+^ regeneration by complex I (CI) (*n* = 4 replicate wells per treatment condition). **c**, Relative aspartate levels and absolute proliferation rates of GOT1/2 DKO 143B cells after switching from 20 mM to 6 mM aspartate in DMEM without pyruvate (*n* = 4 replicate wells per treatment condition). **d**, Relative aspartate levels and absolute proliferation rates of 143B cells in DMEM with 1 mM pyruvate treated with the SDH inhibitor Atpenin A5 (AA5; 5 μM), which directly blocks aspartate synthesis by inhibiting oxidative TCA cycling (*n* = 4 replicate wells per treatment condition). **e**, Comparison of the degree of aspartate rebound, calculated by dividing the GFP:RFP at 72 h by the minimal GFP:RFP measured during an experiment, between the three aspartate limitation paradigms in **b**–**d** (*n* = 4 replicate wells per treatment condition). **f**, Relative aspartate levels and absolute proliferation rates of 143B cells treated with the translation inhibitor cycloheximide (CHX, 1 μg ml^−1^) at 24 h (*n* = 4 replicate wells per treatment condition). **g**, Schematic illustrating a model in which aspartate dynamics are determined by rates of aspartate acquisition (Acq.) and consumption (Cons.), relevant to rotenone and GOT1/2 DKO experiments. Data are represented as mean ± s.d. Statistical significance determined using an ordinary one-way analysis of variance (ANOVA) with uncorrected Fisher’s least significant difference (LSD) and a single pooled variance. All statistics displayed on graphs represent *P* values unless noted otherwise. ABD, aspartate binding domain; NLS, nuclear localization signal; CI, respiratory complex I; Glc, glucose; Gln, glutamine; OAA, oxaloacetate; ASP, aspartate.

To enable time-resolved, non-destructive measurement of relative aspartate abundance in living cells, we employed jAspSnFR3 (22), a genetically encoded aspartate biosensor, which consists of an engineered aspartate binding domain (ABD) linked to a circularly permuted green fluorescent protein (cpGFP) that is activated upon aspartate binding at the ABD (Fig. 1a). Expression of jAspSnFR3 along with a nuclear-localized variant of the red fluorescent protein mRuby2 (NucRFP) produces an experimental system where relative cytosolic aspartate abundance is reported by the green fluorescent protein (GFP):red fluorescent protein (RFP) intensity ratio, and cellular proliferation rates between time points can be calculated using nuclei instances (Fig. 1a)^22^. Measuring both variables simultaneously by live-cell imaging therefore allows us to dissect the temporal relationship between aspartate abundance and cell proliferation in unprecedented detail.

**Extended Data Fig. 1 |:**
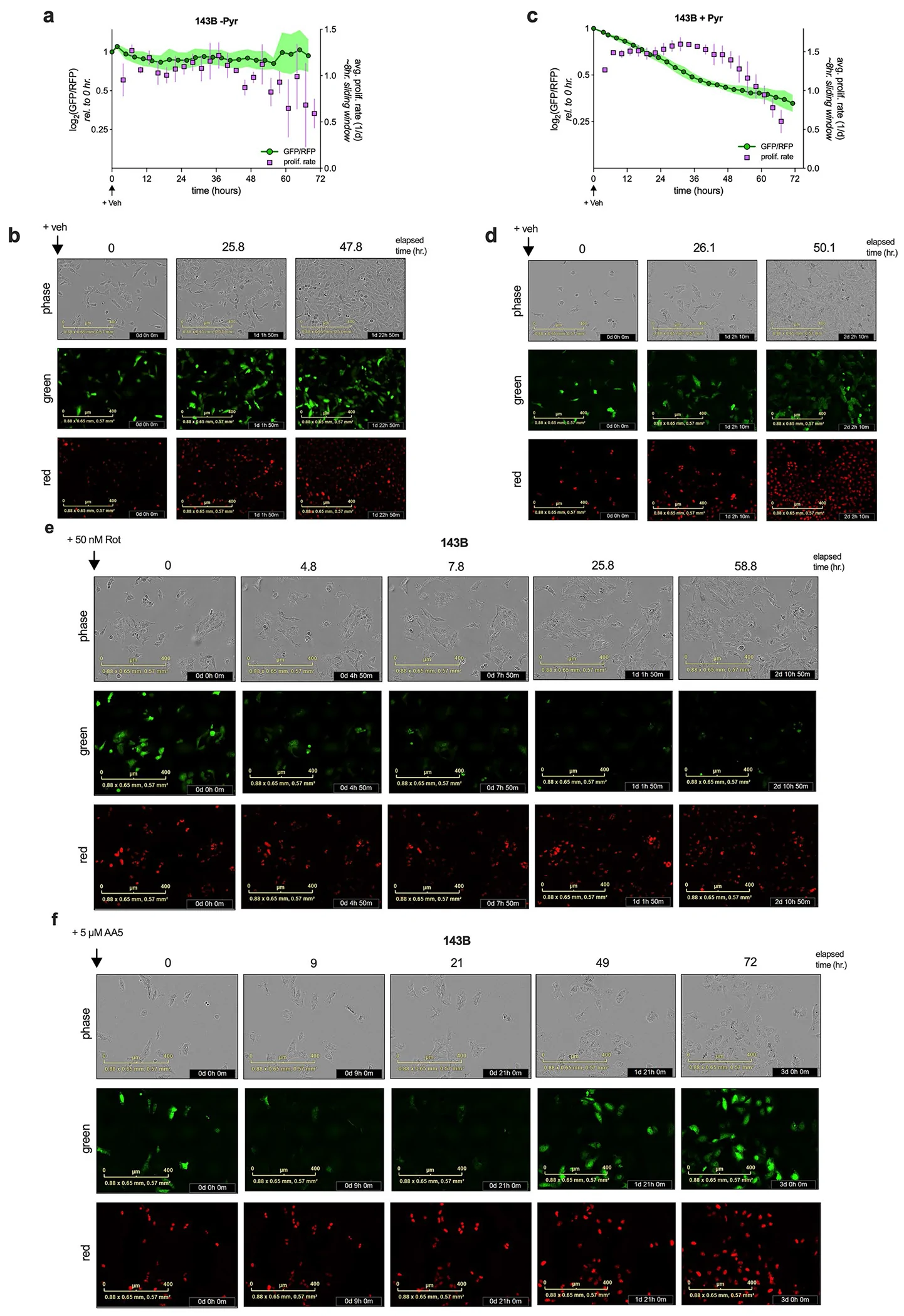
Related to Fig. 1. **a**, relative aspartate levels (green line, left y-axis) and absolute proliferation rates (purple points, right y-axis) of 143B cells in DMEM without pyruvate treated with DMSO (*n* = 4 replicate wells per treatment condition). **b**, representative images from panel (**a**) taken at 0-, 25.8-, and 47.8-h post-treatment. Phase contrast, green (jAspSnFR3), and red (nucRFP) channels are shown. **c**, relative aspartate levels and absolute proliferation rates of 143B cells in DMEM with 1 mM pyruvate treated with DMSO (*n* = 3 replicate wells per treatment condition). **d**, representative images from panel (**c**) taken at 0-, 26.1-, and 50.1-h post-treatment. Phase contrast, green, and red channels are shown. **e**, representative images from the live-cell imaging experiment in Fig. 1b taken at five time points post-treatment. Phase contrast, green, and red channels are shown. **f**, representative images from the live-cell imaging experiment in Fig. 1d taken at five time points post-treatment. Phase contrast, green, and red channels are shown. Data represented as mean +/−S.D.

First, we used this system to examine aspartate/proliferation dynamics in rapidly dividing cells under standard culture conditions. Clonal 143B osteosarcoma cells expressing jAspSnFR3/NucRFP (hereafter referred to as ‘sensor cells’) were seeded onto multi-well plates in Dulbecco’s modified Eagle medium (DMEM), acclimated for 24 h, and then imaged approximately every 3 h for a total of ~72 h in an Incucyte live-cell imaging system (Methods). To assess relative intracellular aspartate levels, total integrated GFP intensity is normalized to the total integrated RFP intensity per well. Meanwhile, nuclei counts are used to calculate average proliferation rates in a sliding ~8-h window throughout the duration of the experiment and plotted alongside relative GFP:RFP intensities (Extended Data Fig. 1a). In control treatments, aspartate levels remained high and relatively unchanged, consistent with the lack of any perturbation and with the notion that cellular aspartate metabolism is at homeostasis. Likewise, for the first ~48 h of the assay, proliferation rates remained stable at expected values for this cell line^7^, after which they become variable and slightly decrease, likely resulting from cells reaching confluency (Extended Data Fig. 1b and Supplementary Video 1). Consistent with the notion that the cells are operating with finite nutrient and space constraints, repeating the experiment in DMEM supplemented with 1 mM pyruvate (a common medium additive that can increase cell proliferation) leads to a more pronounced tapering of proliferation after 48 h, corresponding to confluence, as well as a gradual decrease in aspartate abundance, potentially due to nutrient depletion (Extended Data Fig. 1c,d and Supplementary Video 2)^22^. Overall, these results argue that dual expression of jAspSnFR3 and NucRFP does not significantly alter cell proliferation or metabolism, and that aspartate levels and proliferation are relatively stable in unperturbed cells pre-confluency.

**Extended Data Fig. 2 |:**
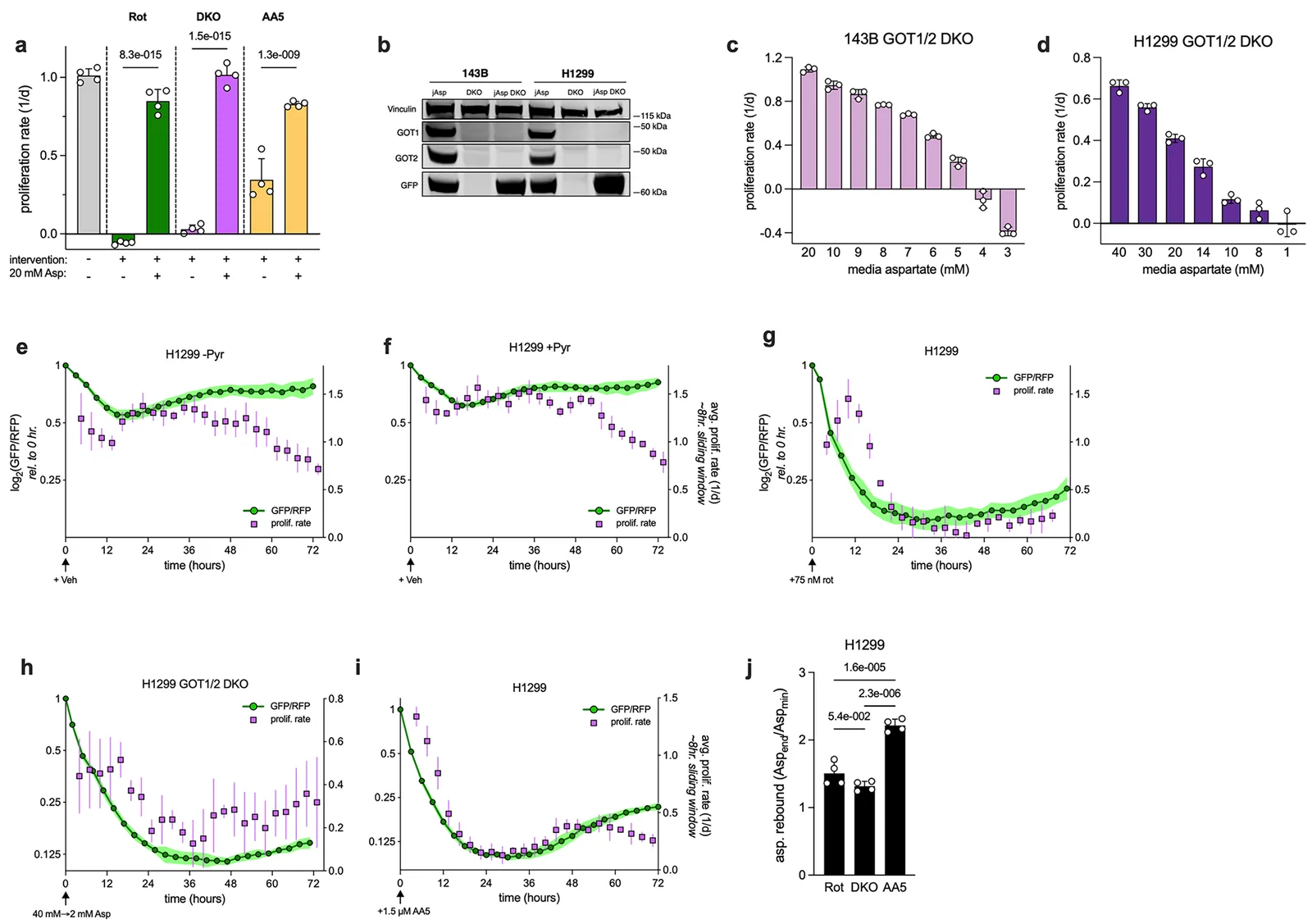
GOT1/2 DKO validation and aspartate/proliferation measurements in H1299 cells. **a**, average proliferation rates (determined using a conventional, end point proliferation assay) of 143B jAspSnFR3/NucRFP or GOT1/2 DKO 143B jAspSnFR3/NucRFP cells under the three paradigms of aspartate limitation [50 nM rotenone (Rot), aspartate withdrawal (5 mM) in GOT1/2 DKOs (DKO), and 5 μM Atpenin A5 (AA5)], with or without 20 mM aspartate (*n* = 4 replicate wells per treatment condition). All conditions used DMEM without pyruvate, with the exception of AA5, which used DMEM with 1 mM pyruvate. **b**, representative Western blots showing expression of vinculin (loading control), GOT1, GOT2, and GFP (jAspSnFR3) in jAspSnFR3-expressing (jAsp), GOT1/2 DKO cells (DKO), and jAspSnFR3-expressing GOT1/2 DKO (jAsp DKO) 143B and H1299 cells. **c-d**, average proliferation rates of 143B (**c**) or H1299 (**d**) GOT1/2 DKO cells (determined using a conventional, end point proliferation assay) in DMEM with the indicated initial aspartate concentrations (*n* = 3 replicate wells per treatment condition). **e-f**, relative aspartate levels (green line, left y-axis) and absolute proliferation rates (purple points, right y-axis) of H1299 cells treated with DMSO in DMEM without pyruvate (e) or with 1 mM pyruvate (f) (*n* = 4 replicate wells per treatment condition). **g**, relative aspartate levels and absolute proliferation rates of H1299 cells treated with 75 nM rotenone in DMEM without pyruvate (*n* = 4 replicate wells per treatment condition). **h**, relative aspartate levels and absolute proliferation rates of GOT1/2 DKO H1299 cells after switching from 40 mM aspartate into 2 mM aspartate in DMEM without pyruvate (*n* = 4 replicate wells per treatment condition). **i**, relative aspartate levels and absolute proliferation rates of H1299 cells in DMEM with 1 mM pyruvate treated with 1.5 μM Atpenin A5 (AA5) (*n* = 4 replicate wells per treatment condition). **j**, comparison of the degree of aspartate rebound measured in the experiments in panels **g**-**i** (*n* = 4 replicate wells per treatment condition). Data represented as mean +/− S.D. Statistical significance determined using an ordinary one-way ANOVA with uncorrected Fisher’s LSD and a single pooled variance. All statistics displayed on graphs represent *P* values unless noted otherwise.

Next, we leveraged this system to investigate temporal changes in aspartate levels and proliferation in several paradigms of aspartate limitation. First, we conducted a similar experiment on sensor cells cultured in DMEM without pyruvate and treated with rotenone, an electron transport chain (ETC) complex I inhibitor that blocks NAD^+^ regeneration from NADH, thereby slowing NAD^+^-dependent reactions in the tricarboxylic acid (TCA) cycle and impairing aspartate synthesis^2–4,9^ (Fig. 1b). Treatment with a dose of rotenone that robustly inhibits CI activity, but whose antiproliferative effects in 143B cells are rescuable with exogenous aspartate^2,7^ (Extended Data Fig. 2a), caused a rapid decay in cellular aspartate levels, which stabilized at a lower abundance after approximately 36 h (Fig. 1b, Extended Data Fig. 1e and Supplementary Video 3). In contrast, proliferation rates remained relatively stable at ~0.5 doublings per day (roughly half that of unperturbed 143B cells) for the first 18 h following rotenone treatment, at which point they shifted to a different, lower pseudo-steady state of ~0.25 doublings per day for the remainder of the assay (Fig. 1b). These results reveal nuanced dynamics in aspartate levels and proliferation that would otherwise be masked in single-time point liquid chromatography–mass spectrometry (LC–MS) experiments or conventional proliferation assays and indicate that cell proliferation responds to rotenone-induced aspartate limitation in two distinct phases.

Next, we sought a paradigm of aspartate limitation that does not rely on pharmacological perturbation. To this end, we leveraged 143B cells lacking both glutamic-oxaloacetic transaminases 1 and 2 (GOT1 and GOT2)—which are aspartate auxotrophs^22^—and generated clonal lines of these cells expressing jAspSnFR3/NucRFP (hereafter referred to as ‘GOT1/2 double knockout (DKO) sensor cells’) (Extended Data Fig. 2b). While GOT1/2 DKO sensor cells are maintained in 20–40 mM aspartate to maximally support cell proliferation, in agreement with previously measured parameters for non-specific aspartate uptake^16,3,5,12,22^, titrating medium aspartate dose-dependently reduced their proliferation rate (Extended Data Fig. 2c), outlining an experimental system in which aspartate acquisition can be modulated independent of its production. We imaged GOT1/2 DKO cells that had acclimated to DMEM containing excess (20 mM) aspartate upon switching into medium with 6 mM aspartate—a concentration that restrains proliferation without overt lethality (Extended Data Fig. 2c). Similar to rotenone, aspartate withdrawal in this system led to an immediate and steady decay of aspartate levels, which largely stabilized by 36 h and increased marginally by the end of the assay (Fig. 1c and Supplementary Video 4). While proliferation rates were variably low for the first 6 h, likely due to cells acclimating after the saline wash at time zero (necessary to remove residual aspartate), proliferation quickly stabilized at a moderately high rate (~0.75 doublings per day) before shifting to a second, lower pseudo-steady state of ~0.25 doublings per day for the remainder of the assay (Fig. 1c).

Overall, aspartate limitation induced by rotenone treatment or aspartate starvation in engineered auxotrophs produces similar aspartate/proliferation dynamics with three salient features: (1) proliferation defects lag changes in aspartate levels, with either paradigm showing an initial ~12–24 h time window in which aspartate levels significantly decrease while proliferation remains constant; (2) proliferation rates show a biphasic pattern, with a higher pseudo-steady state transitioning into a second, lower pseudo-steady state shortly before aspartate levels approach a local minimum; and (3) at the end of the assay, cells exist in a regime where both aspartate levels and proliferation rate are low relative to their initial magnitudes.

Finally, we tested a third paradigm of aspartate limitation centred on inhibition of succinate dehydrogenase (SDH), a TCA cycle enzyme implicated in several cancer types. Notably, SDH loss can also cause aspartate limitation, with metabolic features distinct from other ETC inhibitors^7^. We acclimated sensor cells in DMEM with pyruvate before treating with Atpenin A5 (AA5)—a potent and specific SDH inhibitor that directly inhibits aspartate synthesis from oxidative TCA cycling^7^—and tracked the resulting aspartate/proliferation dynamics (Fig. 1d). Initial aspartate and proliferation kinetics were largely concordant with the other two aspartate limitation paradigms, with a monotonic decrease in aspartate levels accompanying a slightly delayed decrease in proliferation; however, instead of levelling out at a minimum, aspartate levels rebounded starting at ~24 h, reaching a GFP:RFP signal comparable with their initial values by around 48 h (Fig. 1d, Extended Data Fig. 1f and Supplementary Video 5). To better understand the extent of this aspartate rebound in the three aspartate limitation paradigms, we calculated an ‘aspartate rebound’ metric by dividing the final GFP:RFP by the minimum GFP:RFP during the assay per replicate. Comparing this metric among the three paradigms reveals that the relatively modest rebounds in aspartate signal in rotenone-treated cells and aspartate-starved GOT1/2 DKO cells are dwarfed by the rebound in AA5-treated cells (Fig. 1e). Proliferation dynamics upon AA5 treatment also departed from those of rotenone-treated/DKO cells, hitting a local minimum shortly after 24 h, rebounding by ~48 h, and then decreasing again by the end of the assay (Fig. 1d). These dynamics differ from the other two aspartate limitation paradigms both in that aspartate levels/proliferation rates rebound following an initial decay, and in that cells eventually settle into a regime where aspartate levels are partially restored, but proliferation rates remain low.

To generalize these findings beyond 143B cells, we established and analysed the three aforementioned aspartate limitation paradigms in H1299 non-small cell lung cancer cells expressing jAspSnFR3/NucRFP (Extended Data Fig. 2b)^22^. While maximal proliferation rates, aspartate uptake rates, and drug sensitivities differ in this system, aspartate/ proliferation dynamics in all three paradigms were comparable with those of 143B cells (Extended Data Fig. 2d–j).

All three aspartate limitation paradigms suggest that aspartate levels initially drop due to reduced aspartate acquisition (biosynthesis or uptake), while cells continue to consume their aspartate reserves as they generate biosynthetic intermediates to support proliferation. To test whether reducing aspartate consumption without impairing its acquisition would lead to the converse dynamics (that is, an accumulation of aspartate), we treated sensor cells with the protein synthesis inhibitor cycloheximide (CHX) 24 h after the start of an imaging assay. Indeed, CHX treatment caused a rapid decrease in proliferation rate and a corresponding spike in relative aspartate abundance (Fig. 1f), which remained high but decreased towards the end of the assay, likely from feedback inhibition of aspartate synthesis. These results indicate that cellular aspartate dynamics are responsive to both aspartate acquisition and consumption and illustrate that aspartate levels and proliferation rate are not inherently coupled.

In aggregate, our experiments are consistent with a model in which aspartate dynamics in proliferating cells are determined by rates of aspartate acquisition (biosynthesis or uptake) and consumption (Fig. 1g). Cells in standard conditions have matched, high levels of aspartate acquisition and aspartate consumption, leading to a stable aspartate concentration over time. Interventions that impair aspartate acquisition have an immediate but moderate effect on cell proliferation; however, there is an initial time period during which proliferation (which is inherently proportional to aspartate consumption) remains constant, leading to a depletion of aspartate pools. At least in the case of CI inhibition or aspartate starvation in GOT1/2 DKO cells, aspartate pools continue to decrease until they reach a threshold at which aspartate presumably becomes limiting for macromolecular synthesis; aspartate consumption then decreases to match production, and the cells enter a new pseudo-steady state of matched, low aspartate acquisition and consumption (Fig. 1g).

While CI inhibition, GOT1/2 DKO aspartate starvation and SDH inhibition all exert comparable long-term (3–4-day), aspartate-dependent antiproliferative effects (Extended Data Fig. 2a), our results suggest that cellular aspartate and proliferation dynamics are strikingly distinct upon SDH inhibition compared with the other two paradigms. These findings hinted at differences in aspartate acquisition or consumption following SDH inhibition that we further investigated.

### SDH inhibition impairs aspartate utilization into pyrimidine biosynthesis

**Extended Data Fig. 3 |:**
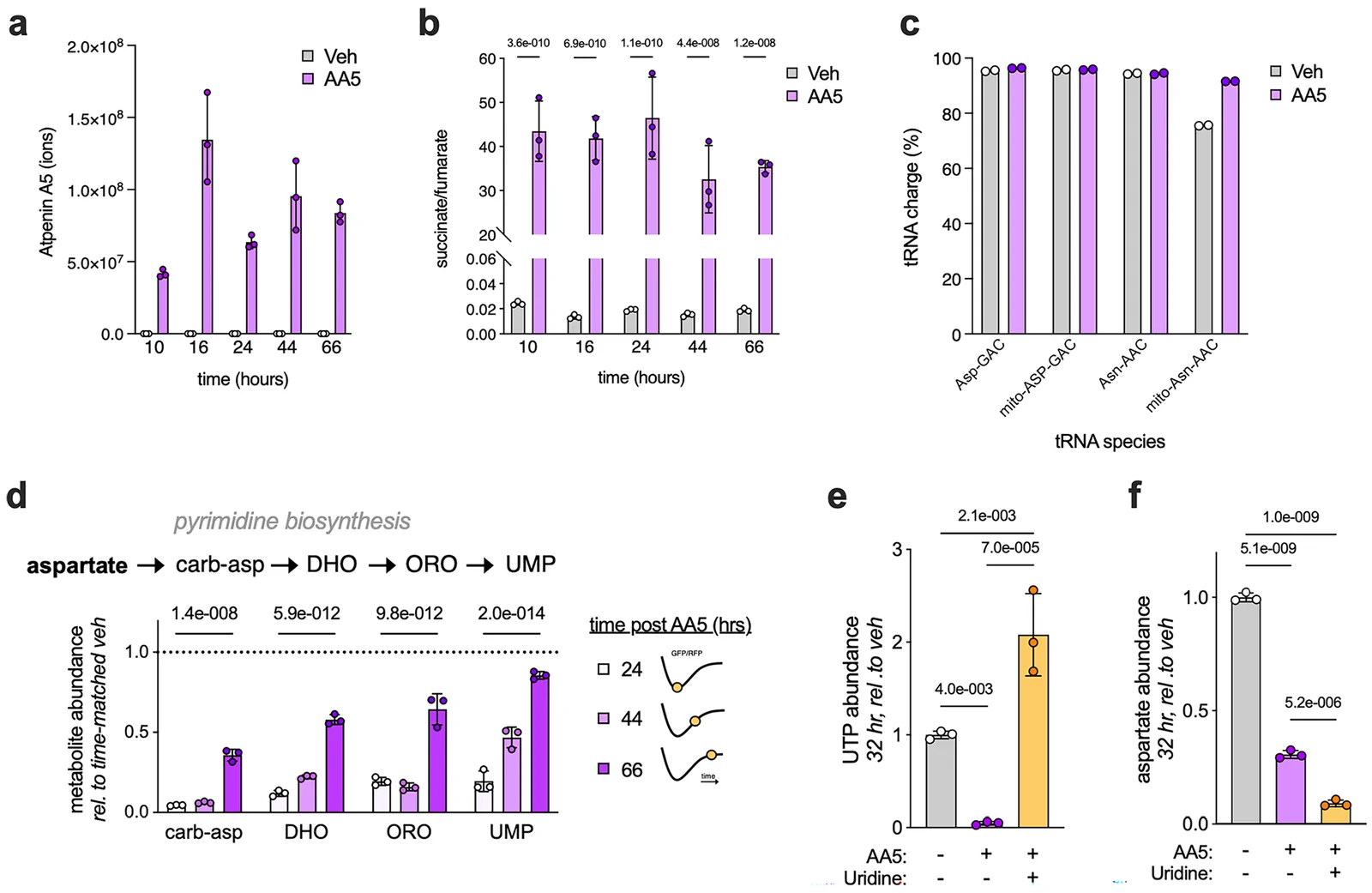
AA5 detection and quantification of aspartate fates in AA5-treated cells. **a**, ion counts of Atpenin A5 (AA5) measured using LC–MS of 143B cells at several time points following treatment with vehicle control or 5 μM AA5 (*n* = 3 replicate wells per treatment condition). **b**, succinate/fumarate ratios measured using LC–MS of 143B cells at several time points following treatment with 5 μM AA5 or vehicle control (*n* = 3 replicate wells per treatment condition). **c**, absolute charge of four cytosolic and mitochondrial aspartate and asparagine tRNA species measured in 143B cells 30 h after treatment with 5 μM AA5 or vehicle control (*n* = 2 replicate wells per treatment condition). **d**, relative abundances of carbamoyl-aspartate (carb-asp), dihydroorotate (DHO), orotate (ORO), and uridine monophosphate (UMP) measured using LC–MS of 143B cells at several time points following treatment with 5 μM AA5 (*n* = 3 replicate wells per treatment condition). Each abundance is normalized to the corresponding time-matched vehicle-treated control; no change compared to vehicle is represented by the dotted line at y = 1. Cartoons illustrate the approximate location of each time point on the prototypical GFP:RFP rebound curve in AA5-treated cells. **e**, relative uridine triphosphate (UTP) levels measured using LC–MS of 143B cells at 32 h post-treatment with vehicle control, 5 μM AA5, or 5 μM AA5 and 200 μM uridine (*n* = 3 replicate wells per treatment condition). **f**, relative aspartate levels measured using LC–MS of 143B cells at 32 h post-treatment with vehicle control, 5 μM AA5, or 5 μM AA5 and 200 μM uridine (*n* = 3 replicate wells per treatment condition). Unless otherwise noted, experiments were conducted in DMEM with 1 mM pyruvate. Data represented as mean +/− S.D. Statistical significance determined using an ordinary two-way ANOVA with uncorrected Fisher’s LSD and a single pooled variance (panels **b**, **e**, and **f**) or ordinary two-way ANOVA with a single pooled variance and Tukey’s multiple comparisons correction (panel **d**). All statistics displayed on graphs represent *P* values unless noted otherwise.

**Fig. 2 |:**
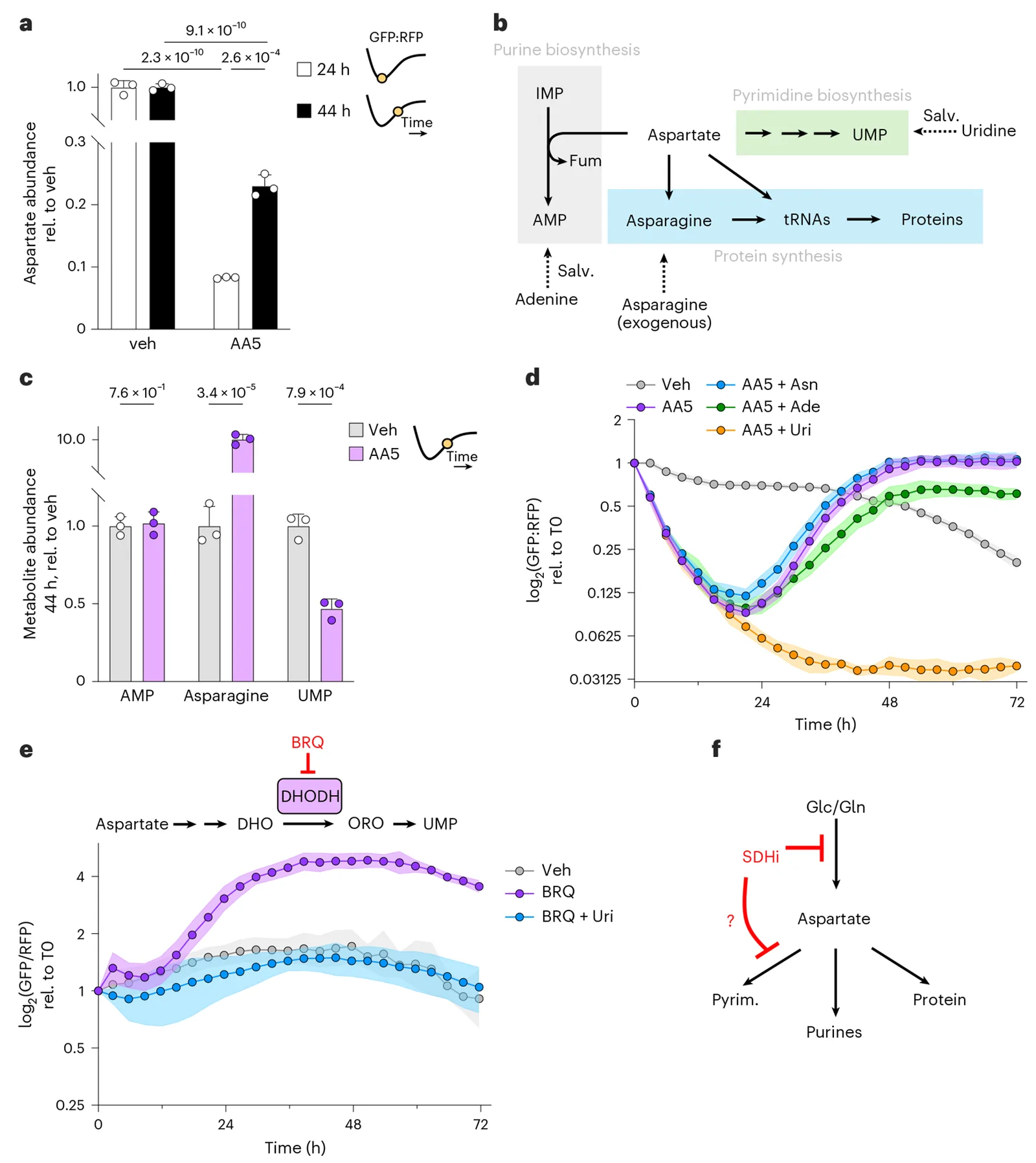
SDH inhibition impairs aspartate utilization into pyrimidine biosynthesis. **a**, Aspartate levels (measured using LC–MS) of 143B cells at 24 h and 44 h post-treatment with 5 μM AA5, each relative to vehicle control (DMSO) (*n* = 3 replicate wells per treatment condition). Cartoons illustrate the approximate location of each time point on the prototypical GFP:RFP rebound curve in AA5-treated cells **b**, Schematic illustrating aspartate fates in our system, including metabolites that can be salvaged (Salv.) into each fate. **c**, Relative abundances of AMP, asparagine and UMP (measured using LC–MS) of 143B cells at 44 h post-treatment with 5 μM AA5 or vehicle control (DMSO) (*n* = 3 replicate wells per treatment condition). The cartoon indicates the position of this time point on the GFP:RFP rebound curve in AA5-treated cells. **d**, Relative aspartate levels of 143B sensor cells treated with vehicle control (DMSO), 5 μM AA5 alone or 5 μM AA5 supplemented with 500 μM asparagine (Asn), 100 μM adenine (Ade) or 200 μM uridine (Uri) (*n* = 4). **e**, Relative aspartate levels of 143B sensor cells treated with vehicle control (DMSO), 2 μM DHODH inhibitor brequinar (BRQ) or 2 μM BRQ and 200 μM uridine (BRQ + Uri) (*n* = 4). **f**, Schematic illustrating the hypothesis that SDH inhibition simultaneously impairs aspartate synthesis and consumption into pyrimidine biosynthesis. Unless otherwise noted, experiments were conducted in DMEM with 1 mM pyruvate. Data are represented as mean ± s.d. Statistical significance determined using an ordinary two-way ANOVA with uncorrected Fisher’s LSD and a single pooled variance (**a**) or multiple unpaired *t*-tests (**c**). All statistics displayed on graphs represent *P* values unless noted otherwise. Fum, fumarate; DHO, dihydroorotate; ORO, orotate.

To confirm the authenticity of the aspartate rebound measured by jAspSnFR3, we quantified relative whole-cell aspartate abundance using LC–MS at different time points following AA5 or vehicle treatment. Consistent with biosensor measurements, aspartate levels were significantly higher at 44 h than 24 h following AA5 treatment (Fig. 2a). We next sought to rule out the possibility that the aspartate rebound was caused by degradation of AA5 and disinhibition of SDH during the imaging assays. To this end, we quantified AA5 itself as well as the succinate/fumarate ratio (a metabolic surrogate for SDH activity^23^) at several time points following AA5 treatment. AA5 was undetectable in vehicle-treated cells and AA5 abundances remained high between 10 and 66 h post-treatment (Extended Data Fig. 3a), arguing against meaningful drug degradation. Meanwhile, AA5 treatment caused a >1,000-fold increase in the succinate:fumarate ratio compared with vehicle-treated controls at all time points, indicating durable SDH inhibition over the entire assay (Extended Data Fig. 3b).

Under the model of aspartate dynamics introduced in the previous section (Fig. 1g), the aspartate rebound ought to arise from increased aspartate acquisition, decreased aspartate consumption or a combination of the two. The short timescale in which the rebound occurs, lack of aspartate in the culture medium and sustained SDH inhibition by AA5 during the assay (Extended Data Fig. 3a,b) argue against an increase in aspartate uptake or synthesis via the canonical route. Instead, we turned our attention to aspartate consumption. Aspartate is consumed for the biosynthesis of proteins (via charging aspartyl-tRNAs), asparagine and both purine and pyrimidine nucleotides (Fig. 2b). To determine whether aspartate consumption into protein synthesis may be affected upon SDH inhibition, we quantified the charge of aspartyl- and asparaginyl-tRNAs in 143B cells treated with AA5 or vehicle using tRNA-seq^24^. This analysis revealed sustained high charging of all relevant tRNA species in AA5-treated cells, arguing against aspartate limitation into protein synthesis as a driver of the aspartate rebound (Extended Data Fig. 3c).

To evaluate the consumption of aspartate in SDH-inhibited cells into asparagine, purine, and pyrimidine biosynthesis, respectively, we quantified relative abundances of asparagine, adenosine monophosphate (AMP) and uridine monophosphate (UMP) at 44 h after AA5 treatment (when aspartate levels are ~mid-rebound). While AMP levels were unchanged and asparagine levels were significantly higher, UMP levels were depleted in AA5-treated cells compared with vehicle controls, suggesting that aspartate consumption into pyrimidines is reduced during the aspartate rebound (Fig. 2c). For a closer look at pyrimidine biosynthesis, we quantified the relative abundances of the pyrimidine intermediates carbamoyl-aspartate, dihydroorotate, orotate and UMP at 24, 44 and 66 h following AA5 treatment in 143B cells using LC–MS. Notably, all four were depleted in AA5-treated cells relative to vehicle-treated controls at each time point (Extended Data Fig. 3d), further suggesting impaired aspartate consumption into de novo pyrimidine synthesis upon SDH inhibition. Interestingly, levels of these metabolites increased towards the end of the experiment, suggesting that pyrimidine synthesis partially recovers over time.

**Extended Data Fig. 4 |:**
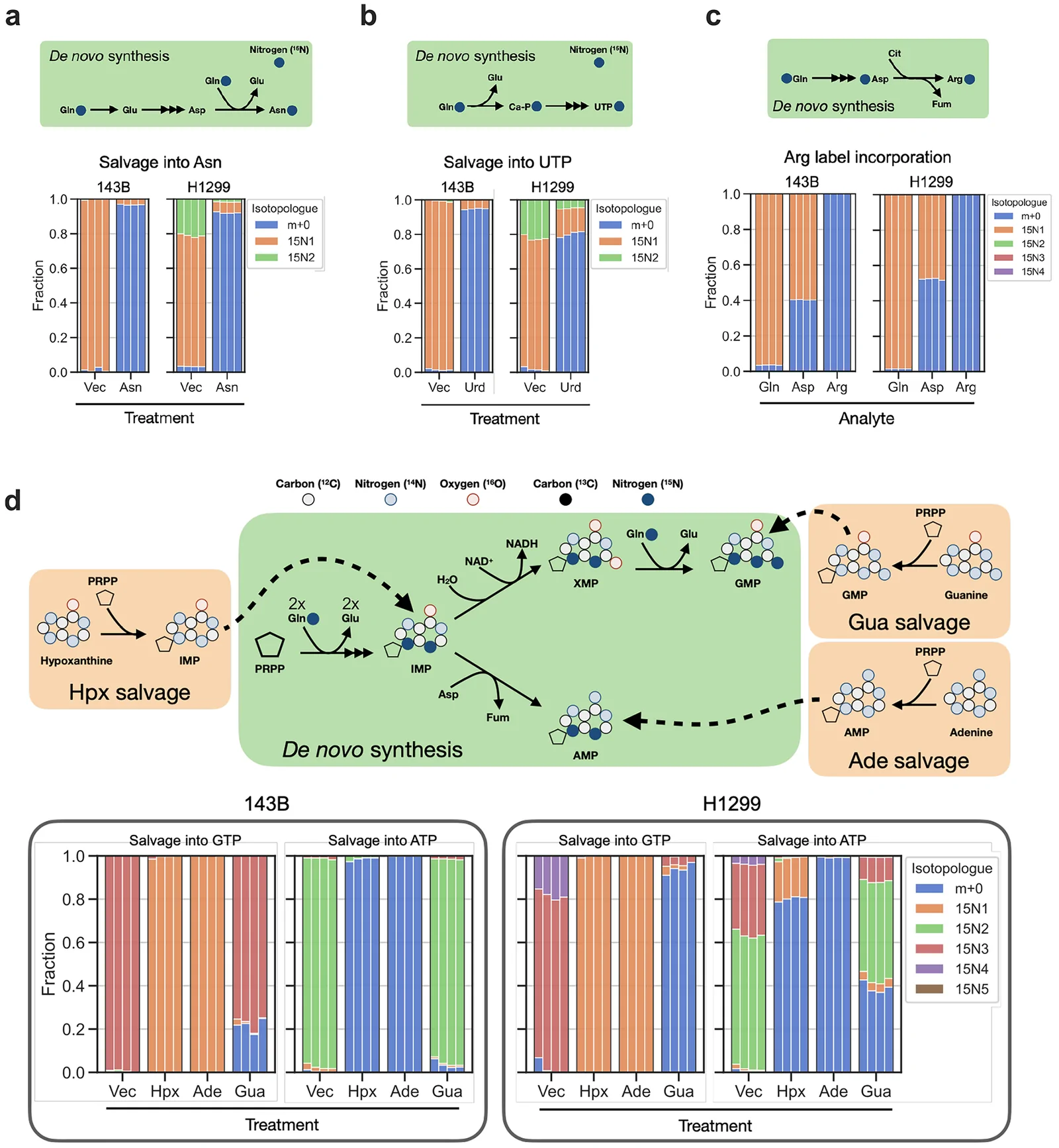
Identification of nutrient conditions to effectively salvage aspartate fates. **a**, diagram demonstrating glutamine (Gln) amide-^15^N label incorporation into asparagine (Asn) and Gln amide-^15^N label incorporation into Asn at steady-state in 143B and H1299 cells grown in DMEM supplemented with vehicle (Vec), or 500 μM Asn (*n* = 4, replicates plotted as individual bars). **b**, diagram demonstrating Gln amide-^15^N label incorporation into uridine triphosphate (UTP) and Gln amide-^15^N label incorporation into UTP at steady-state in 143B and H1299 cells grown in DMEM supplemented with vehicle (Vec), or 200 μM uridine (Urd) (*n* = 4, replicates plotted as individual bars). **c**, diagram demonstrating Gln alpha-^15^N label incorporation into aspartate and subsequently arginine (Arg) and isotopologue distributions for Gln, Asp, and Arg at steady state in 143B and H1299 cells grown in DMEM with no salvageable metabolites added (*n* = 4, replicates plotted as individual bars). **d**, diagram demonstrating Gln amide-^15^N label incorporation into purines and the resulting changes following salvage of hypoxanthine (Hpx), adenine (Ade) or guanine (Gua). AMP/ATP and GMP/GTP are either derived from de novo synthesis or salvage. AMP can be salvaged from adenine directly or through hypoxanthine/IMP as a product of adenine deamination. Bottom isotopologue distributions show Gln amide-^15^N label incorporation into GTP and ATP at steady-state in 143B and H1299 cells grown in DMEM supplemented with vehicle (Vec) or 100 μM Hpx, Ade or Gua (*n* = 4, replicates plotted as individual bars).

Next, we sought to exogenously fulfil each individual aspartate fate and determine the effects on aspartate dynamics following SDH inhibition. Since each fate has discrete uptake and incorporation characteristics, we first investigated the nutrient conditions necessary to bypass the demand for aspartate consumption for the synthesis of asparagine, pyrimidines, and purines. To do so, we used isotope tracing strategies in 143B and H1299 cells to label endogenously produced aspartate fates and then determined the metabolite supplementation conditions that could displace the labelled species, indicating that de novo synthesis from aspartate was no longer necessary. As expected, treatment with unlabelled asparagine suppressed de novo asparagine synthesis and treatment with unlabelled uridine robustly suppressed the contribution of de novo pyrimidine synthesis to the UTP pool (Extended Data Fig. 4a,b). Although aspartate can serve as a nitrogen donor for arginine biosynthesis, many cancer cell lines suppress arginine synthesis and instead import arginine from culture medium^19,25^. Indeed, label incorporation was undetectable from glutamine into arginine (Extended Data Fig. 4c). Among purine nucleobase treatments, adenine was sufficient to meet all purine demands, bypassing the aspartate consumption step specific to adenylate nucleotide synthesis and supporting guanylate nucleotide production, presumably through deamination to generate inosine monophosphate (IMP), and bypassing the earlier aspartate consumption step common to all de novo synthesized purines (Extended Data Fig. 4d). Altogether, this investigation identified concentrations of exogenous adenine, uridine and asparagine that can efficiently fulfil the non-protein metabolic fates of aspartate in this system (Fig. 2b).

We then conducted a 72-h imaging assay in which sensor cells were treated with AA5 and either asparagine, adenine or uridine to determine the effects on aspartate dynamics. While adenine and asparagine did not substantially affect the aspartate rebound, uridine supplementation completely abolished it, resulting in monotonic aspartate depletion over the course of the assay (Fig. 2d). Using LC–MS, we confirmed that uridine supplementation significantly reduced aspartate levels in AA5-treated cells and restored UTP levels (Extended Data Fig. 3e,f), corroborating the sensor results and indicating that pyrimidine deficiency is necessary for the aspartate rebound effect upon SDH inhibition.

Finally, we sought to determine whether inhibition of pyrimidine synthesis in this system is sufficient to increase aspartate levels. To do so, we used brequinar (BRQ), a specific inhibitor of the dihydroorotate dehydrogenase (DHODH) step of pyrimidine synthesis^26,27^. Interestingly, BRQ treatment alone caused a spike in aspartate levels with similar temporal kinetics as the AA5-mediated rebound that was negated by uridine co-treatment, suggesting that pyrimidine synthesis impairment is sufficient to cause an aspartate rebound (Fig. 2e)^2 ,28^. Altogether, these results argue that aspartate levels rebound following SDH inhibition due to a specific impairment of pyrimidine nucleotide biosynthesis. The fact that pyrimidine biosynthesis intermediates remain depleted from 24 to 44 h after AA5-treament (Extended Data Fig. 3d) despite a significant increase in aspartate levels during this time period (Fig. 2a) suggests that SDH inhibition causes a secondary metabolic effect that impairs de novo pyrimidine biosynthesis beyond simply limiting aspartate availability (Fig. 2f).

### Distributed and hierarchical metabolic growth limitations upon SDH inhibition

**Extended Data Fig. 5 |:**
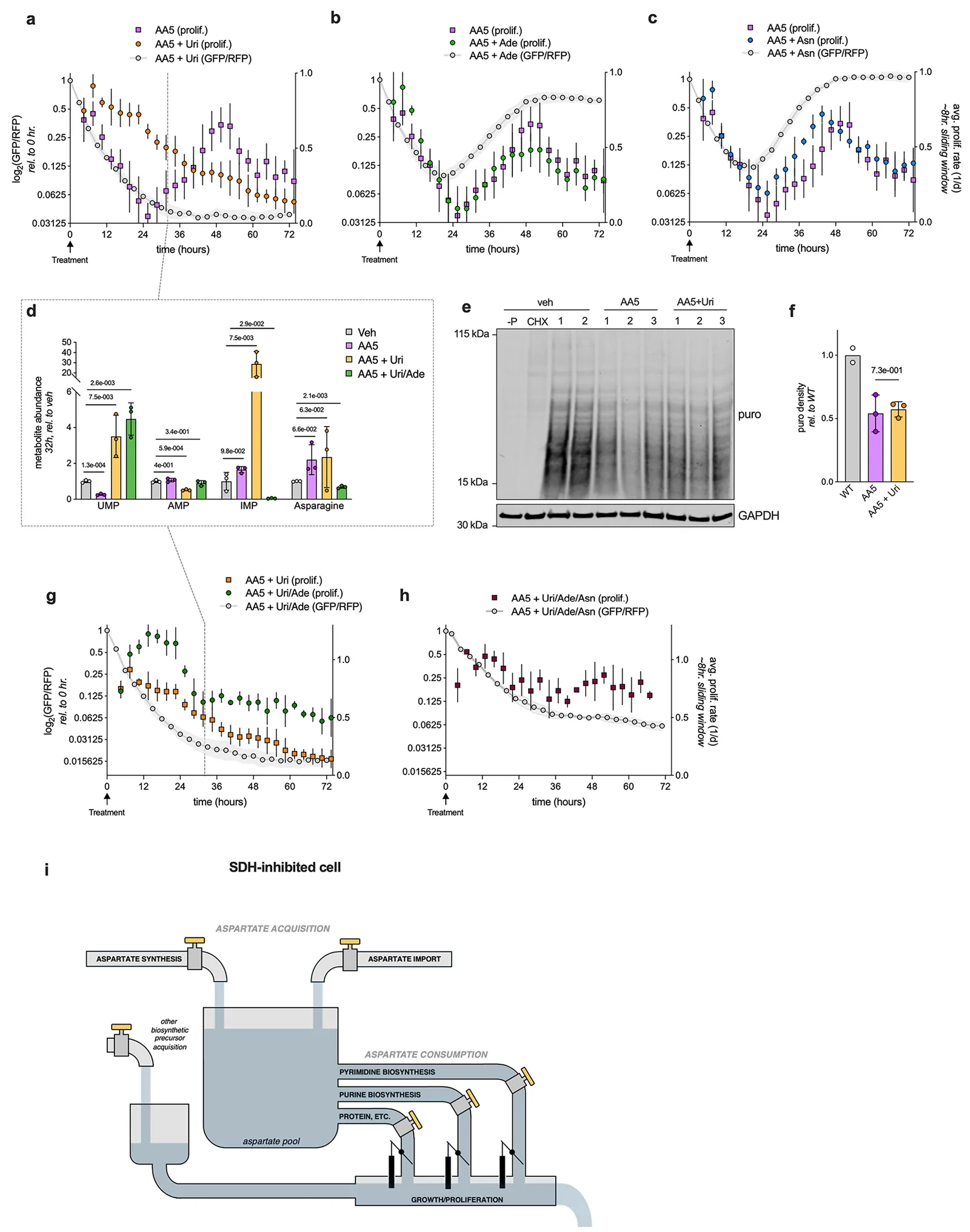
Distributed and hierarchical metabolic growth limitations upon SDH inhibition. **a**, relative aspartate levels (grey line, left y-axis) and absolute proliferation rates (individual points, right y-axis) of 143B sensor cells treated with 5 μM AA5 with or without 200 μM uridine (Uri) (*n* = 4 replicate wells per treatment condition). Dotted line marks time point corresponding to data in panel **d**. **b**, relative aspartate levels and absolute proliferation rates of 143B sensor cells treated with 5 μM AA5 with or without 100 μM adenine (Ade) (*n* = 4 replicate wells per treatment condition). **c**, relative aspartate levels and absolute proliferation rates of 143B sensor cells treated with 5 μM AA5 with or without 500 μM asparagine (Asn) (*n* = 4 replicate wells per treatment condition). **d**, relative levels of UMP, AMP, IMP, and asparagine (Asn) measured using LC–MS in 143B cells 32 h after treatment with vehicle control, 5 μM AA5, 5 μM AA5 with 200 μM uridine (Uri), or 5 μM AA5 with 200 μM uridine (Uri) and 100 μM adenine (Ade) (*n* = 3 replicate wells per treatment condition). **e**, 24-hour puromycin incorporation assay using 143B cells treated with vehicle control (veh), 5 μM AA5, or 5 μM AA5 with 200 μM uridine (Uri). -P denotes a no-puromycin control, and CHX denotes cells treated with 1 μg/ml of the translation inhibitor cycloheximide. GAPDH serves as a loading control (*n* = 2 for veh, *n* = 3 for AA5 +/− Uri). **f**, quantification of relative puromycin lane densities in panel **e** (*n* = 2 technical replicates for veh condition, *n* = 3 technical replicates for AA5 and AA5+Uri conditions). **g**, relative aspartate levels and absolute proliferation rates of 143B sensor cells treated with 5 μM AA5 and 200 μM uridine (Uri, reproduced from panel **a**) or 200 μM uridine and 100 μM adenine (Uri/Ade). Dotted line marks time point corresponding to data in panel **d** (*n* = 4). **h**, relative aspartate levels and absolute proliferation rates of 143B sensor cells treated with 5 μM AA5, 200 μM uridine, 100 μM adenine, and 500 μM asparagine (Uri/Ade/Asn) (*n* = 4 replicate wells per treatment condition). **i**, schematic illustrating a conceptual model of aspartate metabolism in SDH-inhibited cells. The aspartate pool is filled by aspartate acquisition (consisting of aspartate synthesis and import) and drained as aspartate is consumed into pyrimidines, purines, and protein, which have an obligatory ‘order.’ Upon constraining aspartate acquisition, aspartate levels fall until they no longer fulfil pyrimidine demands, which closes the gate to stop cell growth/proliferation regardless of the flow through the other aspartate fate/biosynthesis precursor pipes. Note that complementing any single aspartate fate pipe (for example, by supplementing uridine) permits continued aspartate consumption until levels fall to limit the next lowest fate pipe. Unless otherwise noted, experiments were conducted in DMEM with 1 mM pyruvate. Data represented as mean +/− S.D. Statistical significance determined using multiple two-sided unpaired t-tests and the two-stage step-up method of Benjamini, Krieger, and Yekutieli to account for multiple comparisons (panel **d**) or an ordinary one-way ANOVA with uncorrected Fisher’s LSD and a single pooled variance (panel **f**). All statistics displayed on graphs represent *P* values unless noted otherwise.

The fact that salvageable aspartate fates differed in preventing the aspartate rebound posed the question of how their supplementation impacts cell proliferation upon SDH inhibition. Interestingly, uridine prevented the decrease in proliferation rate during the initial 24 h following AA5 treatment, after which proliferation rates monotonically decreased instead of rebounding as observed in the AA5 only treatment condition (Extended Data Fig. 5a). Adenine and asparagine, however, did not substantially affect proliferation dynamics following AA5 co-treatment (Extended Data Fig. 5b,c), consistent with their lack of impact on aspartate dynamics (Fig. 2d). These data suggest that uridine initially solves the proximal proliferation defect from SDH inhibition, but that cells then run into a secondary, aspartate-related metabolic limitation as aspartate levels are further consumed for proliferation. Indeed, LC–MS at 32 h following AA5/uridine co-treatment revealed that AMP (but not asparagine) was significantly depleted in AA5/uridine co-treated cells relative to controls (Extended Data Fig. 5d). IMP, the metabolite immediately upstream of the aspartate-dependent step in purine synthesis (Fig. 2b), was also drastically accumulated, consistent with a purine deficiency due to reduced aspartate levels impairing IMP to AMP conversion, as has been found in other settings of aspartate limitation^2,16^ (Extended Data Fig. 5d).

To verify that these cells were not also deficient in the proteogenic aspartate fates at this time, we measured relative protein synthesis rates using a puromycin incorporation assay 24 h following treatment with vehicle, AA5 or AA5/uridine. While both AA5-containing treatments had reduced puromycin incorporation relative to vehicle-treated cells, there was no significant difference between AA5 and AA5/uridine-treated conditions (Extended Data Fig. 5e,f), indicating that uridine treatment does not further impair protein synthesis in AA5-treated cells at this time point.

To functionally test whether purine deficiency is responsible for the proliferation decrease upon AA5/uridine co-treatment, we measured aspartate/proliferation dynamics in cells treated with AA5 and both uridine and adenine. Uridine/adenine co-supplementation further improved proliferation to ~1 doubling per day for 24 h following AA5 treatment, after which proliferation shifted to a lower but relatively stable ~0.5 doublings per day for the remainder of the assay (Extended Data Fig. 5g), consistent with uridine supplementation precipitating a secondary purine deficiency in SDH-impaired cells. Notably, GFP:RFP levels still monotonically decreased throughout the assay, suggesting that continued aspartate consumption into its other fates (asparagine/protein synthesis) still outpaced acquisition in this context.

Finally, to test whether the proliferation shift in AA5/uridine/adenine treated cells may reflect an emergent deficiency in asparagine synthesis upon pyrimidine/purine rescue, we measured aspartate/proliferation dynamics after additionally supplementing with asparagine. Consistent with this hypothesis, proliferation rates in AA5/uridine/adenine/asparagine-treated cells were sustained at around 0.8 doublings per day throughout the entire assay, whereas aspartate levels still decayed but plateaued at relatively higher levels (Extended Data Fig. 5h). Overall, these results reveal that the metabolic growth limitations in cells upon SDH inhibition are (1) distributed, with no single aspartate fate able to provide a sustained proliferation benefit in the absence of the other fates, but also (2) hierarchical, with pyrimidine deficiencies superseding purine deficiencies, which supersede deficiencies in the proteogenic aspartate fates. This is consistent with a model whereby aspartate fates are all required for sustained cellular proliferation despite exhibiting different ‘aspartate thresholds’ at which they become impaired. Rescuing a single fate permits continued aspartate consumption until aspartate levels become limiting for the next fate in the hierarchy (Extended Data Fig. 5i).

### Succinate inhibits mammalian ATCase

**Fig. 3 |:**
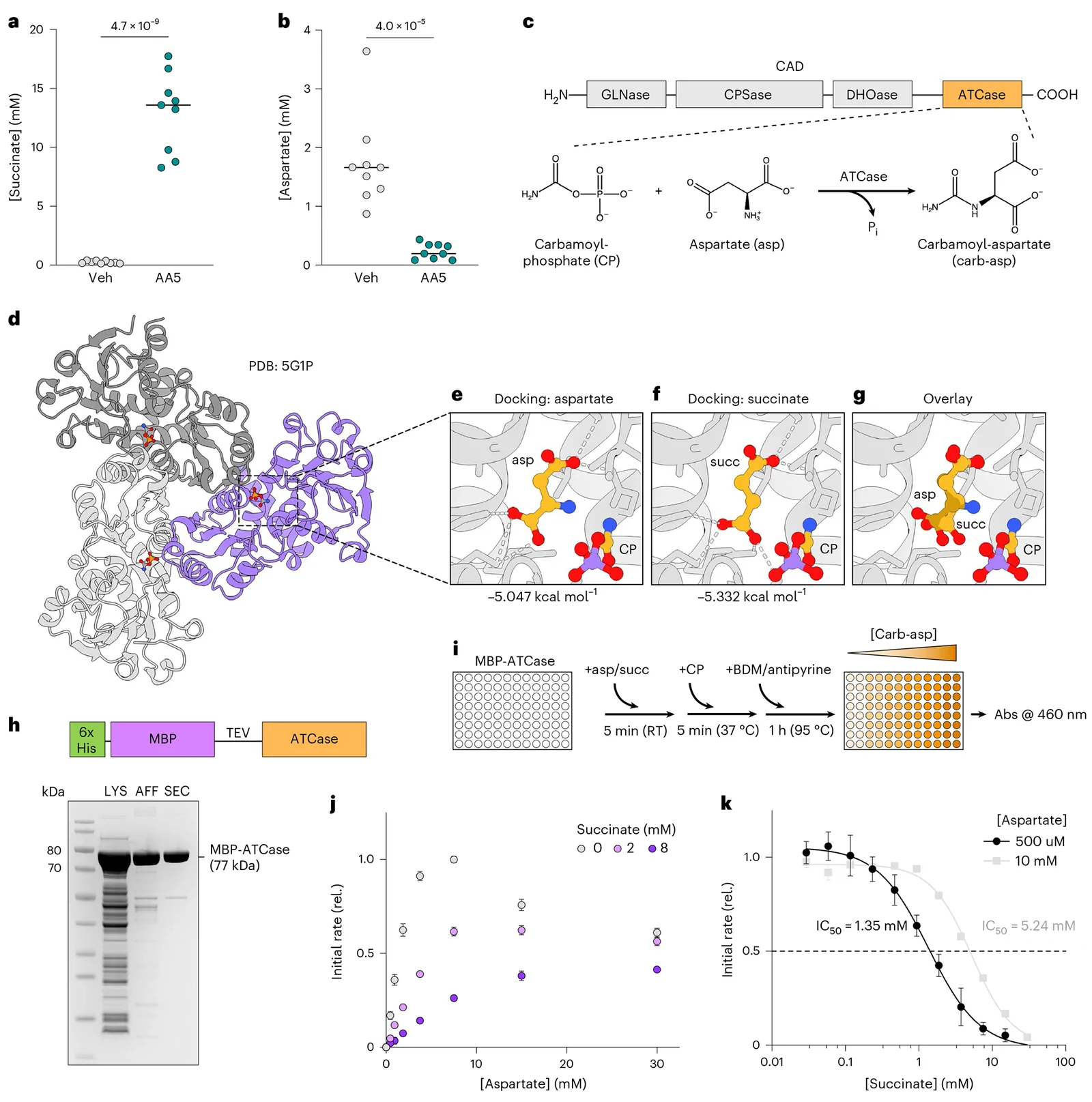
Succinate inhibits mammalian ATCase. **a**,**b**, Whole-cell succinate (**a**) and aspartate (**b**) concentrations in 143B cells measured using LC–MS at multiple time points following treatment with vehicle or 5 μM AA5 (*n* = 9 replicate wells, multiple time points from several different LC–MS experiments). **c**, A schematic of the mammalian CAD protein with the C-terminal ATCase domain highlighted, and the reaction catalysed by ATCase. **d**, A previously solved crystal structure of the mammalian ATCase trimer (PDB: 5G1P) bound to CP, coloured by monomer, which was used for molecular docking analyses. **e**–**g**, Predicted binding poses and affinities of aspartate (**e**), succinate (**f**) and aspartate/succinate (**g**) in the substrate binding pocket of CP-bound mammalian ATCase. Ligands are coloured by atom: yellow, carbon; red, oxygen; blue, nitrogen; purple, phosphorus. **h**, A schematic of the 6×His-tagged MBP-ATCase construct used in subsequent panels and a Coomassie-stained protein gel demonstrating expression and purification of MBP-ATCase. LYS, cleared bacterial lysate; AFF, elute following affinity purification; SEC, elute following size-exclusion chromatography.**i**, Schematic illustrating the absorbance-based ATCase activity assay, which measures accumulation of the reaction product, carbamoyl-aspartate (Methods). **j**, Representative activity assay depicting initial enzymatic rates of purified MBP-ATCase incubated with 10 mM CP and the indicated concentrations of aspartate and succinate. Rates are normalized to the 0 mM succinate and 7.5 mM aspartate condition (*n* = 3 technical replicates). **k**, Representative activity assay depicting initial enzymatic rates of purified MBP-ATCase incubated with 10 mM CP and the indicated concentrations of succinate at either 500 μM or 10mM aspartate (*n* = 3 technical replicates). Rates are normalized to the 0 mM succinate conditions for each respective aspartate concentration and fit to a sigmoidal curve using Prism. Data are represented as mean ± s.d. Statistical significance determined using two-sided unpaired *t*-tests. All statistics displayed on graphs represent *P* values unless noted otherwise. asp, aspartate; succ, succinate; BDM, butanedione monoxime.

In SDH-impaired cells, pyrimidine precursors are depleted and generally remain depleted compared with vehicle-treated controls even as aspartate levels recover, suggesting that SDH inhibition alters metabolism in a way that specifically disfavours pyrimidine synthesis beyond simply limiting aspartate availability. A key metabolic distinction between SDH inhibition and other causes of aspartate limitation is the accumulation of succinate, the substrate of SDH. In addition to its role as a TCA cycle intermediate, succinate is an ‘oncometabolite’ known to have multiple biochemical effects, including competitively inhibiting various α-ketoglutarate-dependent dioxygenases involved in oxygen sensing and DNA/histone demethylation^29^. To rigorously determine the effects of SDH inhibition on succinate and aspartate in our system, we used quantitative LC–MS to estimate whole-cell concentrations of these metabolites in 143B cells treated with AA5 or vehicle control, combining multiple treatment durations. We found that vehicle-treated 143B cells maintain median succinate levels around 200 μM and aspartate levels around 1.7 mM, consistent with other measurements of unperturbed mammalian cells^22,30^. SDH inhibition increased succinate levels by approximately 70-fold, reaching a median concentration of around 14 mM, which is comparable to what has been measured in SDH-deficient tumours and cell lines^31–34^ (Fig. 3a). Meanwhile, SDH inhibition decreased aspartate concentrations by roughly eightfold (with some variability depending on time point) to a median concentration of approximately 200 μM (Fig. 3b). These results confirm that SDH inhibition in our system both depletes aspartate and dramatically increases cellular succinate concentrations, motivating us to search for mechanistic links between succinate and pyrimidine biosynthesis.

**Extended Data Fig. 6 |:**
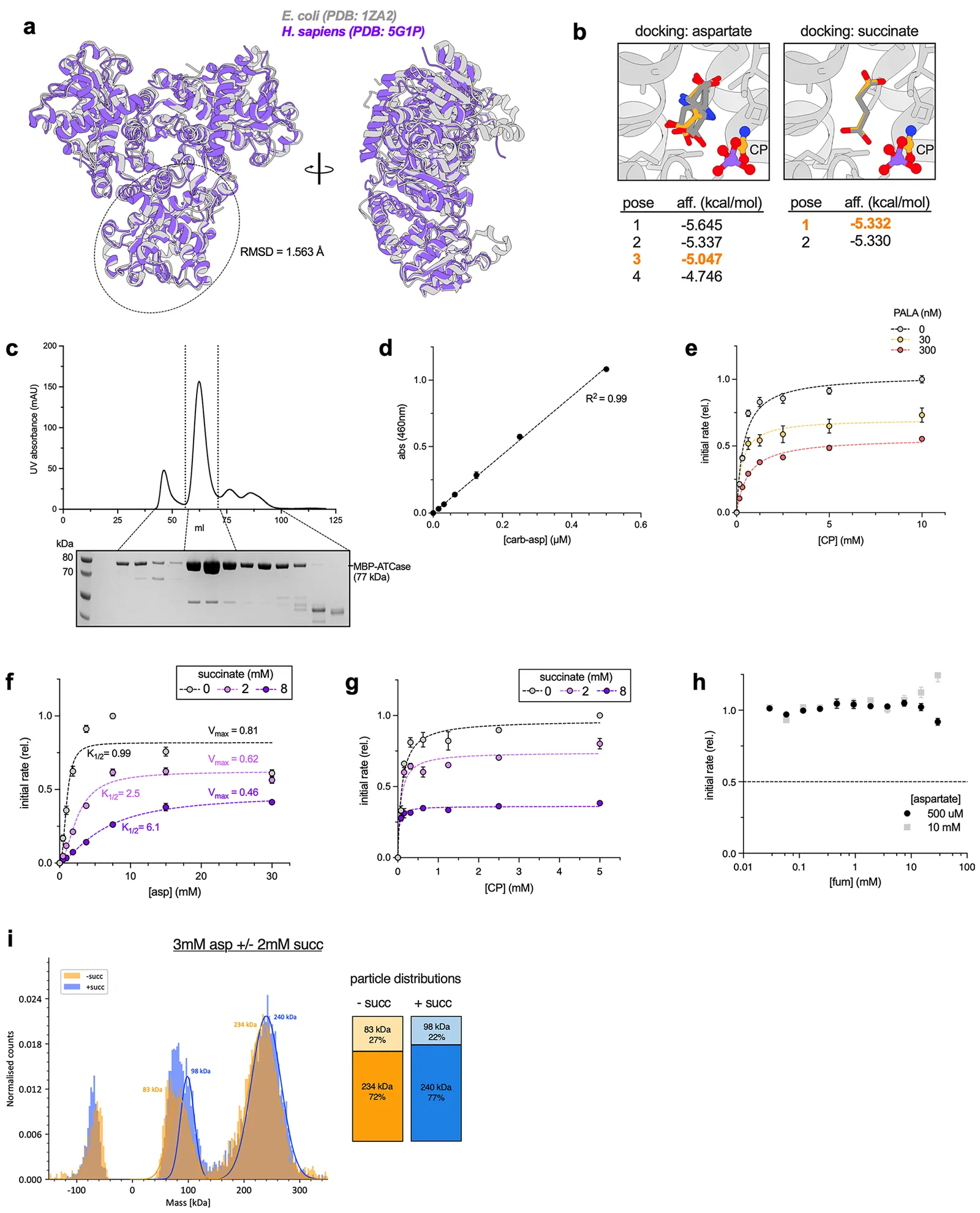
Related to Fig. 3. **a**, alignment of previously solved crystal structures of *E. coli* (PDB :1ZA2) and human (PDB: 5G1P) ATCase catalytic trimers. Root mean square deviation (RMSD) reflects structural similarity between the circled monomers. **b**, predicted binding poses and affinities for the top four-scoring aspartate poses and the top two-scoring succinate poses using molecular docking analyses. Poses depicted in Fig. 3 are highlighted in yellow. Ligands are coloured by atom: yellow/grey = carbon, red = oxygen, blue = nitrogen, purple = phosphorus **c**, UV absorbance trace from size-exclusion chromatography (SEC) of MBP-ATCase affinity chromatography elute, with accompanying Coomassie-stained protein gel measuring MBP-ATCase abundance in the fraction ranges indicated by the dotted lines. Vertical dotted lines indicate fractions taken for downstream analyses. **d**, representative calibration curve for ATCase activity assay plotting absorbance as a function of carbamoyl-aspartate (carb-asp) concentration and fit with linear regression using Prism (*n* = 3 technical replicates). **e**, initial enzymatic rates for purified MBP-ATCase incubated with 5 mM aspartate and the indicated concentrations of CP and PALA (*n* = 3 technical replicates). Data are fit to a Michaelis-Menten model using Prism and rates are normalized to the 10 mM CP, 0 nM PALA condition. **f**, data from Fig. 3j fit with cooperative sigmoidal curves using Prism. V_max_ and K_1/2_ values are shown next to the respective conditions (*n* = 3 technical replicates). **g**, initial enzymatic rates for purified MBP-ATCase incubated with 10 mM aspartate and the indicated concentrations of CP and succinate (*n* = 3 technical replicates). Data are fit to a Michaelis-Menten model using Prism and rates are normalized to the 10 mM CP, 0 nM succinate condition. **h**, initial enzymatic rates for purified MBP-ATCase incubated with 10 mM CP and the indicated concentrations of aspartate and fumarate (fum) (*n* = 3 technical replicates). Rates are normalized to the 0 mM fumarate condition for each respective aspartate concentration. **i**, particle size distributions obtained using mass photometry on isolated MBP-ATCase incubated with 5 mM CP, 3 mM aspartate, and with or without 2 mM succinate (+/− succ). Predicted molecular weights are labelled above the corresponding Gaussian fits. **j**, quantification of particle distributions in (i), excluding peaks which are mirrored on either side of 0 kDa, a common buffer artefact seen in MP experiments. Data represented as mean +/− S.D. All statistics displayed on graphs represent *P* values unless noted otherwise.

SDH inhibition depletes carbamoyl-aspartate and downstream pyrimidine intermediates (Extended Data Fig. 3d), suggesting that impairment occurs at the first step of pyrimidine synthesis—the formation of carbamoyl-aspartate from aspartate and carbamoyl-phosphate— which is catalysed by aspartate transcarbamylase (ATCase) (Fig. 3c). Interestingly, several classic enzymological studies noted that succinate can competitively inhibit *Escherichia coli* ATCase in vitro with a *K*_i_ in the range of ~0.4–20 mM, depending on pH^35–37^. To our knowledge, succinate inhibition of mammalian ATCase has not been described, nor has this interaction been observed in living cells. Nonetheless, as bacterial and human ATCase share notable sequence and structural similarity, including at the active site^38^ (Extended Data Fig. 6a), we reasoned that succinate-mediated inhibition of ATCase could explain the effects of SDH impairment on pyrimidine biosynthesis.

Despite sharing a catalytic fold, bacterial and human ATCase are non-identical and differ in some notable respects. Bacterial ATCase is a standalone enzyme that forms catalytic homotrimers flanked by regulatory subunits, which dimerize to form dodecamers, whereas human ATCase comprises the C-terminal domain of a mega-enzyme called CAD (carbamoyl-phosphate synthetase 2, aspartate transcarbamylase and dihydroorotase) that combines the catalytic activities of the first three steps in pyrimidine biosynthesis and likely trimerizes via the ATCase domain^39^ (Fig. 3c). To evaluate if succinate could bind the ATCase substrate pocket in a manner similar to aspartate, we performed molecular docking analyses using a published X-ray crystal structure of the ATCase domain of human CAD^38^. For this analysis, we chose the structure of carbamoyl phosphate (CP)-bound human ATCase (Fig. 3d), as CP binds ATCase before aspartate and prepares the binding pocket to accept aspartate^38,40^. As we could not find a structure of human ATCase bound to both CP and aspartate, we first docked aspartate into the substrate pocket of CP-bound ATCase. This analysis revealed several top-scoring poses that orient the bound aspartate with its α-amino group proximal to the carbonyl of CP, consistent with the proposed ATCase reaction mechanism^40^ (Fig. 3e). Repeating this docking analysis with succinate returned two top-scoring poses in which succinate occupies nearly the same pose as aspartate, minus the α-amino group (Fig. 3f,g and Extended Data Fig. 6b). Notably, the predicted binding affinities for succinate and aspartate are similar (−5.332 and −5.047 kcal mol^−1^, respectively), supporting the hypothesis that succinate acts as a substrate-analogue inhibitor of human ATCase.

To test this hypothesis experimentally, we cloned, recombinantly expressed and purified the ATCase domain of human CAD fused N-terminally to a 6×His-tagged maltose binding protein (MBP) (Fig. 3h). Size-exclusion chromatography of affinity-purified MBP-ATCase revealed a single major peak at the expected mass of the trimer (~241 kDa) (Extended Data Fig. 6c), confirming that the MBP tag did not prevent oligomerization. Using an adapted plate-based ATCase activity assay^41^ that relies on a colorimetric readout of carbamoyl-aspartate formation (Fig. 3i and Extended Data Fig. 6d), we measured relative initial enzymatic rates of MBP-ATCase in excess aspartate and a titration of CP. This showed saturation kinetics that were consistent with a previous study^38^. Furthermore, initial rates were dose-dependently decreased with nanomolar doses of the classical ATCase inhibitor, PALA (*N*-phosphonacetyl-L-aspartate)^42^, as expected (Extended Data Fig. 6e). Next, we measured initial enzymatic rates of MBP-ATCase in excess CP, a titration of aspartate, and three concentrations of succinate (0, 2 and 8 mM). This analysis revealed a potent and dose-dependent inhibition of enzyme activity by succinate (Fig. 3j).

The fact that human ATCase exhibits positive cooperativity with regard to aspartate and that high aspartate concentrations paradoxically inhibit human ATCase activity^38^ (Fig. 3j) prevents a straightforward modelling of our enzyme kinetic data and assigning of a single *K*_i_ value for succinate. Nevertheless, fitting the aforementioned activity data to sigmoidal curves suggests that succinate increases *K*_1/2_ and decreases *V*_max_, consistent with mixed inhibition (Extended Data Fig. 6f); meanwhile, succinate inhibits ATCase non-competitively with regard to CP (Extended Data Fig. 6g). To better delineate the relationship between enzyme activity, succinate and aspartate, we performed an activity assay in excess CP and fixed the aspartate concentration at two values: 500 μM, slightly above the median whole-cell aspartate concentration we measured in SDH-inhibited cells (Fig. 3b) and 10 mM, representing a supraphysiological aspartate concentration. Titrating succinate in these conditions revealed a sigmoidal dependence between succinate concentration and enzyme activity, with IC_50_ values for succinate of 1.35 mM and 5.24 mM at 500 μM and 10 mM aspartate, respectively (Fig. 3k). Finally, we repeated this experiment with fumarate, which differs from succinate only by a central double bond, and saw no appreciable inhibition of ATCase at fumarate levels up to 30 mM (Extended Data Fig. 6h). Thus, succinate specifically inhibits human ATCase in a manner that depends on aspartate concentration, with IC50 values that are well below the median whole-cell succinate concentrations in SDH-inhibited cells.

One mechanism by which succinate could inhibit human ATCase is by disrupting its trimerization, which is essential for catalysis as adjacent monomers contribute to substrate pocket formation^43^ (Fig. 3d). To address this possibility, we used mass photometry (MP) to query the particle size distribution of purified MBP-ATCase in solution with excess CP and sub-saturating concentrations of aspartate, with or without 2 mM succinate. Under both substrate conditions, MP revealed bimodal particle size distributions consistent with an equilibrium between monomeric and trimeric MBP-ATCase. The addition of succinate did not substantially change this equilibrium—and potentially even slightly favoured trimer formation (Extended Data Fig. 6i)— arguing that succinate does not inhibit human ATCase by disrupting oligomerization. Overall, our in vitro results reveal that succinate can inhibit human ATCase in a manner that is at least partially competitive with aspartate and argue strongly for succinate accumulation as the driver of pyrimidine biosynthesis impairment in SDH-inhibited cells.

### Metabolic control of aspartate and succinate abundance defines ATCase activity in cells

**Extended Data Fig. 7 |:**
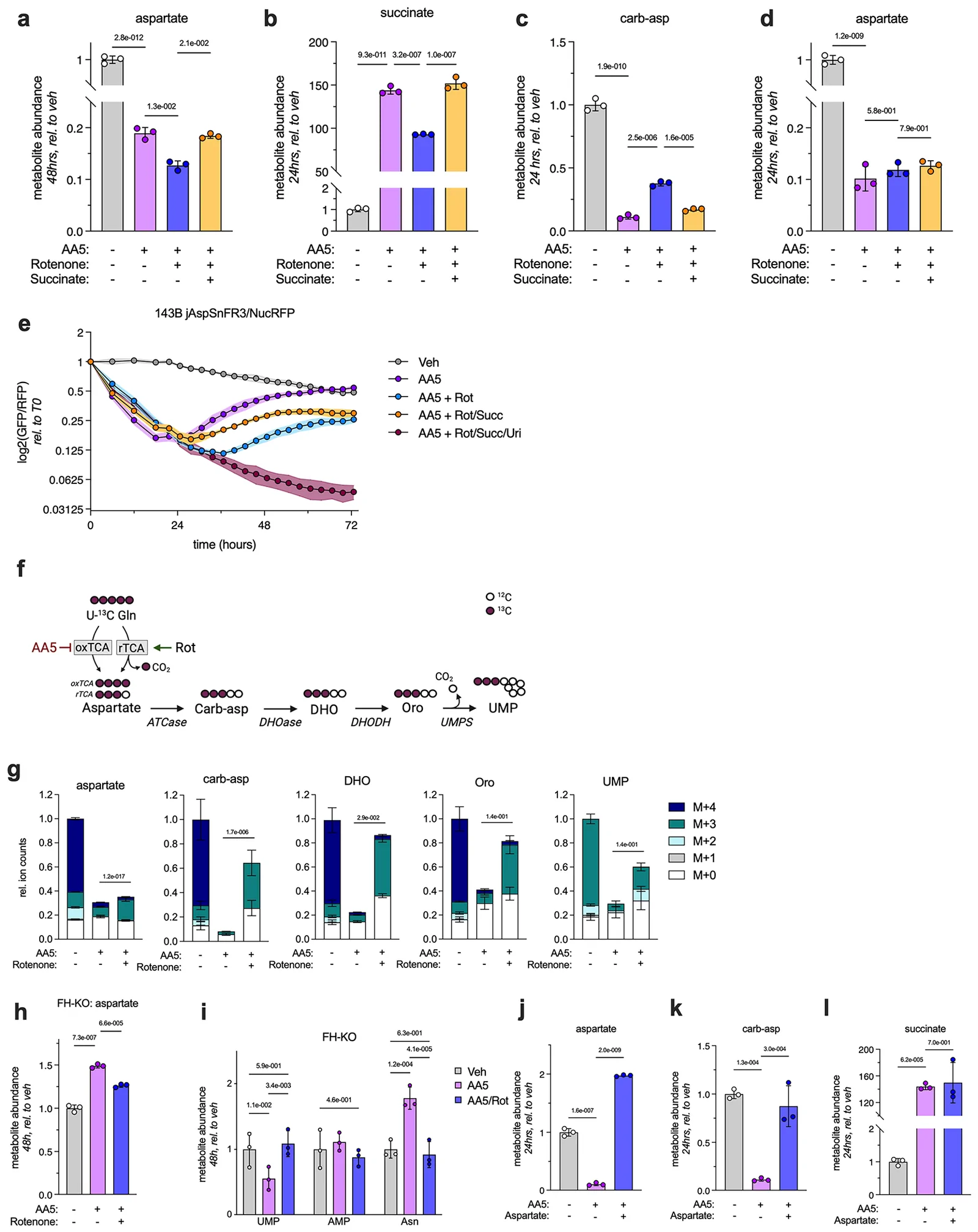
Related to Fig. 4. **a**, relative aspartate abundances measured by LC–MS on 143B cells 48 h after treatment with vehicle control or the indicated combinations of 5 μM AA5, 50 nM rotenone, and 10 mM succinate (*n* = 3 replicate wells per treatment condition). **b–d**, relative succinate (b), carbamoyl-aspartate (c), and aspartate (d) abundances measured by LC–MS on 143B cells 24 h after treatment with vehicle control or the indicated combinations of 5 μM AA5, 50 nM rotenone, and 10 mM succinate (*n* = 3 replicate wells per treatment condition). **e**, relative aspartate levels of 143B sensor cells treated for 72 h with the indicated combinations of 5 μM AA5, 50 nM rotenone (Rot), 10 mM succinate (Succ), and 200 μM uridine (Uri) (*n* = 3). **f**, schematic illustrating U-^13^C glutamine labelling into aspartate and downstream pyrimidine synthesis intermediates via reductive TCA cycling (rTCA), which is stimulated by rotenone co-treatment^7^. **g**, isotopologue distributions of aspartate, carbamoyl-aspartate (carb-asp), dihydroorotate (DHO), orotate (ORO), and uridine monophosphate (UMP) after 30 h of U-^13^C glutamine tracing in 143B cells treated with vehicle control, 5 μM AA5, or 5 μM AA5 and 50 nM rotenone (*n* = 3 replicate wells per treatment condition). **h**, relative aspartate abundances measured by LC–MS on FH-KO 143B cells 48 h after treatment with vehicle control, 5 μM AA5, or 5 μM AA5 and 50 nM rotenone (*n* = 3 replicate wells per treatment condition). **i**, relative AMP and asparagine (Asn) abundances measured by LC–MS on FH-KO 143B cells 48 h after treatment with vehicle control, 5 μM AA5, or 5 μM AA5 and 50 nM rotenone (Rot) (*n* = 3 replicate wells per treatment condition). **j-l**, relative aspartate (j), carbamoyl-aspartate (carb-asp) (k), and succinate (l) abundances measured by LC–MS on 143B cells 24 h after treatment with vehicle control, 5 μM AA5, or 5 μM AA5 and 20 mM aspartate (*n* = 3 replicate wells per treatment condition). Unless otherwise noted, experiments were conducted in DMEM with 1 mM pyruvate. Data represented as mean +/− S.D. Statistical significance determined using an ordinary two-way ANOVA with uncorrected Fisher’s LSD and a single pooled variance, except panel **h**, which used an ordinary two-way ANOVA with main effects only, uncorrected Fisher’s LSD and a single pooled variance. All statistics displayed on graphs represent *P* values unless noted otherwise. For panel **h**, *P* values denote the results of statistical testing on the M + 3 fraction of each metabolite. ATCase, aspartate transcarbamylase; DHOase, dihydroorotase; DHODH, dihydroorotate dehydrogenase; UMPS, UMP synthase.

**Fig. 4 |:**
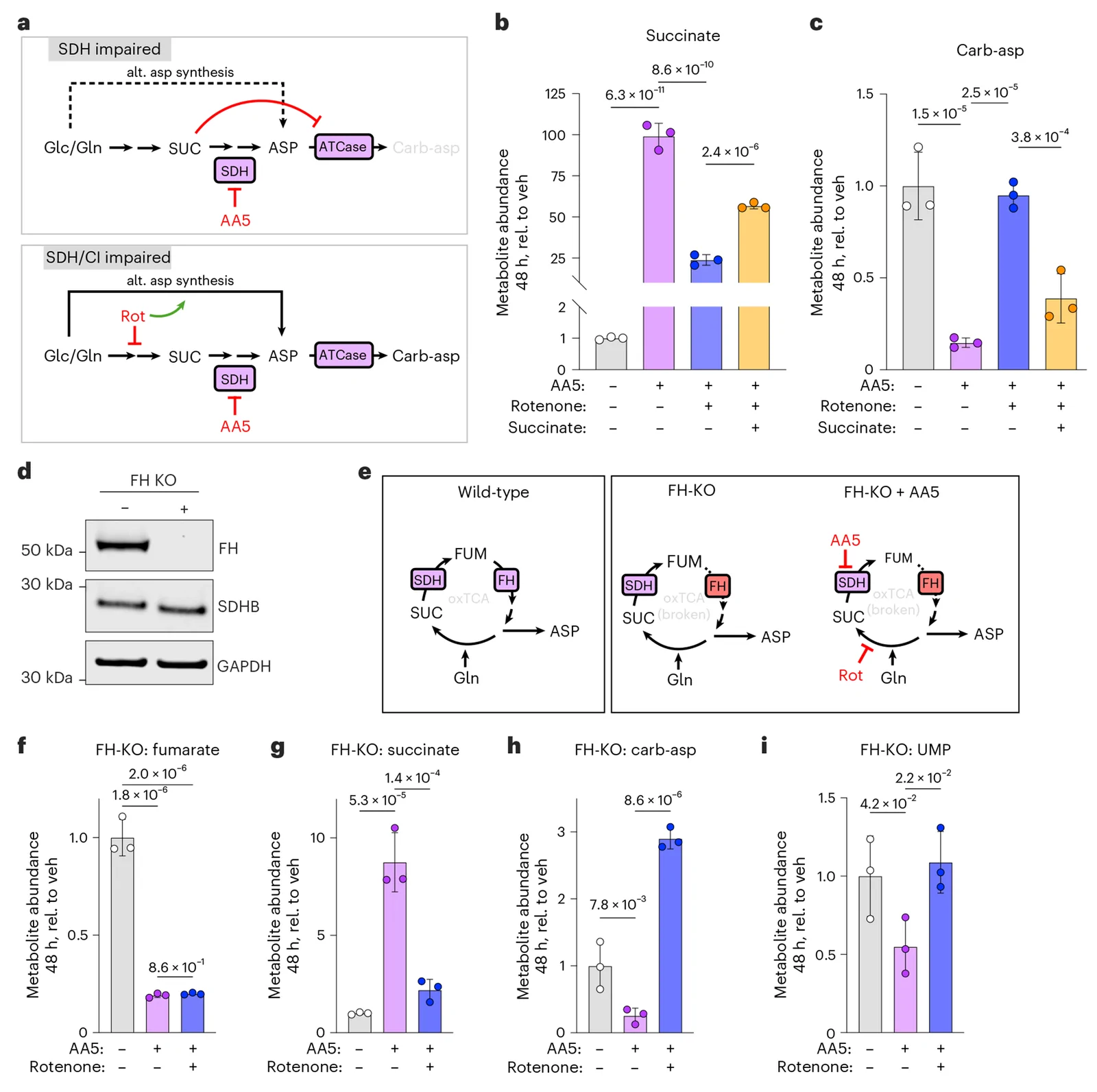
Metabolic control of aspartate and succinate abundance defines ATCase activity in cells. **a**, Schematic illustrating the proposed effects of AA5 and rotenone (Rot) on aspartate synthesis and ATCase activity in cells. **b**, Relative succinate abundances measured by LC–MS on 143B cells 48 h after treatment with vehicle control or the indicated combinations of 5 μM AA5, 50 nM rotenone and 10 mM succinate. **c**, Relative carbamoyl-aspartate (carb-asp) abundances measured by LC–MS on 143B cells 48 h after treatment with vehicle control or the indicated combinations of 5 μM AA5, 50 nM rotenone and 10 mM succinate. **d**, Western blot showing levels of FH, SDHB and GAPDH loading control in 143B FH-KO cells and parental 143B cells. **e**, Schematic illustrating the TCA cycle status of FH-KO cells compared with wild-type parental cells and describing the effects of AA5/rotenone treatment in FH-KO cells. **f**–**i**, Relative fumarate (**f**), succinate (**g**), carbamoyl-aspartate (**h**) and UMP (**i**) abundances measured by LC–MS on 143B FH-KO cells 48 h after treatment with vehicle control or the indicated combinations of 5 μM AA5 and 50 nM rotenone. *n* = 3 biological replicates for all panels; data are represented as mean ± s.d. Unless otherwise noted, experiments were conducted in DMEM with 1 mM pyruvate. Statistical significance was determined using an ordinary two-way ANOVA with uncorrected Fisher’s LSD and a single pooled variance. All statistics displayed on graphs represent *P* values unless noted otherwise. SUC, succinate; FUM, fumarate; oxTCA, oxidative TCA cycle; UMP, uridine monophosphate.

We previously reported that SDH-inhibited cells benefit from co-inhibition of ETC complex I (CI), which improves cell proliferation by decreasing mitochondrial NAD^+^/NADH to promote alternative aspartate synthesis^7^. CI inhibition is also expected to decrease succinate levels by slowing the activity of the NAD^+^-dependent α-ketoglutarate dehydrogenase (αKGDH) enzyme ^,44^, which may benefit SDH-impaired cells by lowering succinate to disinhibit ATCase (Fig. 4a). First, we confirmed that the CI inhibitor rotenone significantly decreases succinate levels in AA5-treated cells, an effect which could be partially reversed by succinate supplementation (Fig. 4b). Next, we examined carbamoyl-aspartate abundance in these conditions as a metabolic indicator of ATCase activity; indeed, CI inhibition significantly increased carb-asp levels at 48 h post-treatment, and succinate supplementation largely blunted this effect (Fig. 4c). Aspartate levels were slightly lower in SDH/CI impaired cells at this time point and increase upon treatment with exogenous succinate (Extended Data Fig. 7a), further demonstrating a metabolic interaction between impaired pyrimidine synthesis and aspartate levels. We also confirmed these effects were mostly present at 24 h post AA5/rotenone co-treatment (Extended Data Fig. 7b–d). Evaluating these treatments in 143B sensor cells, we found that rotenone co-treatment delays and suppresses the AA5-induced aspartate rebound, consistent with rotenone partially restoring pyrimidine synthesis, and that succinate addition reactivates the rebound, whereas uridine supplementation abolishes it completely (Extended Data Fig. 7e). To further validate that rotenone restores pyrimidine synthesis to SDH-inhibited cells, we traced U-^13^C glutamine into pyrimidine synthesis intermediates at 32 h in cells treated with vehicle, AA5 or AA5/rotenone (Extended Data Fig. 7f,g). As expected, levels of most pyrimidine intermediates were significantly higher in AA5/rotenone-treated cells, with isotopologue distributions consistent with increased biosynthesis from aspartate (Extended Data Fig. 7g). Overall, these results indicate that CI suppression disinhibits ATCase in SDH-impaired cells by reducing succinate levels and further supports a model whereby cellular ATCase activity is influenced by succinate abundance.

For an orthogonal system in which we could modulate cellular succinate abundance and probe effects on ATCase activity, we used CRISPR/Cas9 to generate monoclonal 143B cells deficient in fumarate hydratase (FH), the TCA cycle enzyme immediately downstream of SDH (Fig. 4d). FH-KO cells are similar to SDH-KO cells in that they have no oxidative TCA cycling; however, they differ in their preferential accumulation of fumarate, which does not inhibit ATCase in vitro (Extended Data Fig. 6h), to a greater degree than succinate (Fig. 4e)^45^. We found that treating FH-KO cells with AA5 decreased fumarate levels by nearly tenfold and similarly increased succinate levels (Fig. 4f,g), effectively transforming their predominant ‘fumarate accumulation’ phenotype into a ‘succinate accumulation’ phenotype (Fig. 4e). AA5 treatment in FH-KO cells increased aspartate and decreased carbamoyl-aspartate levels (Extended Data Fig. 7h and Fig. 4h), resulting in a corresponding UMP deficiency (Fig. 4i) but not deficiencies in AMP or asparagine (Extended Data Fig. 7i). Rotenone co-treatment rescued these effects, consistent with their dependence on succinate accumulation (Fig. 4g–i and Extended Data Fig. 7h,i). Overall, these data suggest that SDH inhibition suppresses ATCase activity and aspartate consumption into pyrimidine synthesis in FH-KO cells by increasing succinate levels. These results also argue that pyrimidine synthesis defects are not a generalized consequence of oxidative TCA cycle dysfunction, but rather a specific consequence of the succinate accumulation that is characteristic of SDH impairment^31^.

Finally, our biosensor and in vitro data demonstrate that succinate serves at least partially as a competitive inhibitor of ATCase, suggesting that increasing aspartate concentrations should be sufficient to re-establish ATCase activity in SDH-impaired cells. Consistent with this idea, exogenous aspartate supplementation rescued the decreases in aspartate and carbamoyl-aspartate following AA5 treatment without affecting succinate abundance (Extended Data Fig. 7j–l). Altogether, these results further pinpoint aspartate and succinate as important metabolic determinants governing ATCase activity and pyrimidine biosynthesis in living cells.

### SDH inhibition causes replication stress by impairing pyrimidine synthesis

**Extended Data Fig. 8 |:**
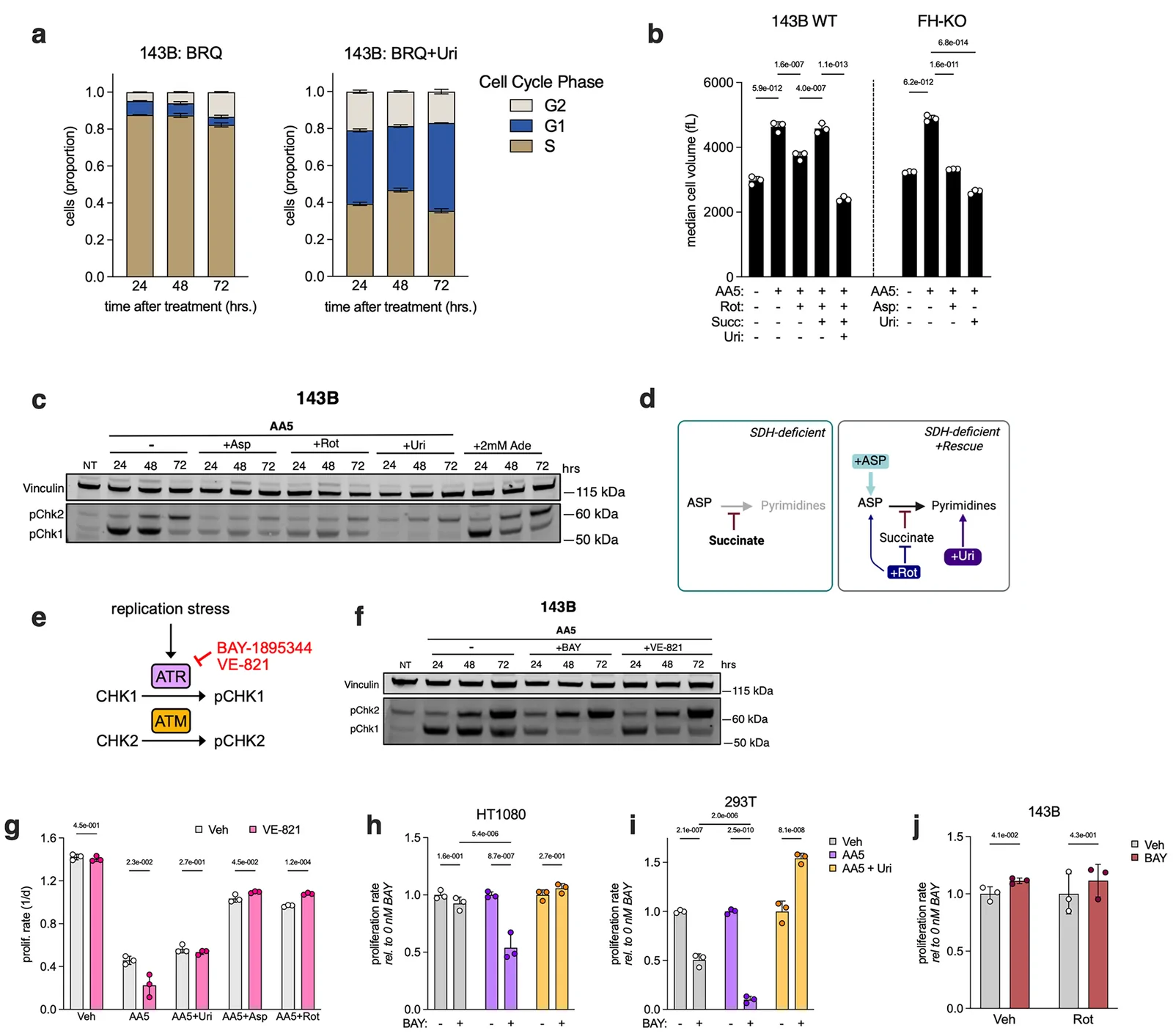
Related to Fig. 5. **a**, representative cell cycle experiment showing the proportion of 143B cells in each cell cycle phase at the indicated time points following treatment with 2 μM brequinar (BRQ) with or without 200 μM uridine (Uri) (*n* = 3 replicate wells per treatment condition). **b**, median cell volumes of 143B wild-type (WT) or 143B FH knockout (FH-KO) cells after 72 h of treatment with the indicated combinations of 5 μM AA5, 50 nM rotenone (Rot), 20 mM succinate (Succ), 200 μM uridine (Uri), and 20 mM aspartate (Asp) (*n* = 3 replicate wells per treatment condition). **c**, representative Western blot demonstrating levels of phosphorylated CHK1 (pCHK1), phosphorylated CHK2 (pCHK2), and vinculin loading control in 143B cells at the indicated time points following treatment with control (NT), 2 mM adenine (Ade), 5 μM AA5, or 5 μM AA5 with either 20 mM aspartate (Asp), 50 nM rotenone (Rot), or 200 μM uridine (Uri). **d**, schematic illustrating various metabolic rescues of pyrimidine synthesis in SDH-deficient cells. **e**, identical schematic as Fig. 5e, with the addition of the ATR inhibitor VE-821 and ATM kinase, which phosphorylates CHK2. **f**, representative Western blot demonstrating levels of phosphorylated CHK1 (pCHK1), phosphorylated CHK2 (pCHK2), and vinculin loading control in 143B cells at the indicated time points following treatment with control (NT), 5 μM AA5, or 5 μM AA5 with either 20 nM BAY-1895344 or 0.6 μM VE-821. This blot is representative of three independent experiments from separate cell lysates, all showing the same results. **g**, absolute proliferation rates (measured using a conventional, 72-hour end point proliferation assay) of 143B cells treated with vehicle control or 0.6 μM VE-821 and the indicated combinations of vehicle control, 5 μM AA5, 200 μM uridine (Uri), 20 mM aspartate (Asp), and 50 nM rotenone (Rot) (*n* = 3 replicate wells per treatment condition). **h-i**, relative proliferation rates (measured using a conventional, 72-hour end point proliferation assay) of HT1080 (h) and 293 T (i) cells treated with the indicated combinations of 5 μM AA5 and either 10 nM (h) or 50 nM (i) BAY-1895344 (BAY) (*n* = 3 replicate wells per treatment condition). **j**, relative proliferation rates (measured using a conventional, 72-hour end point proliferation assay) of 143B cells treated with the indicated combinations of 50 nM rotenone (Rot) and either vehicle control or 20 nM BAY-1895344 (BAY) in DMEM without pyruvate (*n* = 3 replicate wells per treatment condition). Unless otherwise noted, experiments were conducted in DMEM with 1 mM pyruvate. Data represented as mean +/− S.D. Statistical significance determined using an ordinary one-way ANOVA with Tukey’s multiple comparisons test and a single pooled variance (panel **b**), ordinary two-way ANOVAs with uncorrected Fisher’s LSD and a single pooled variance (panels **h**-**i**) or multiple two-sided unpaired t-tests (panels **g**, **j**). All statistics displayed on graphs represent *P* values unless noted otherwise.

**Fig. 5 |:**
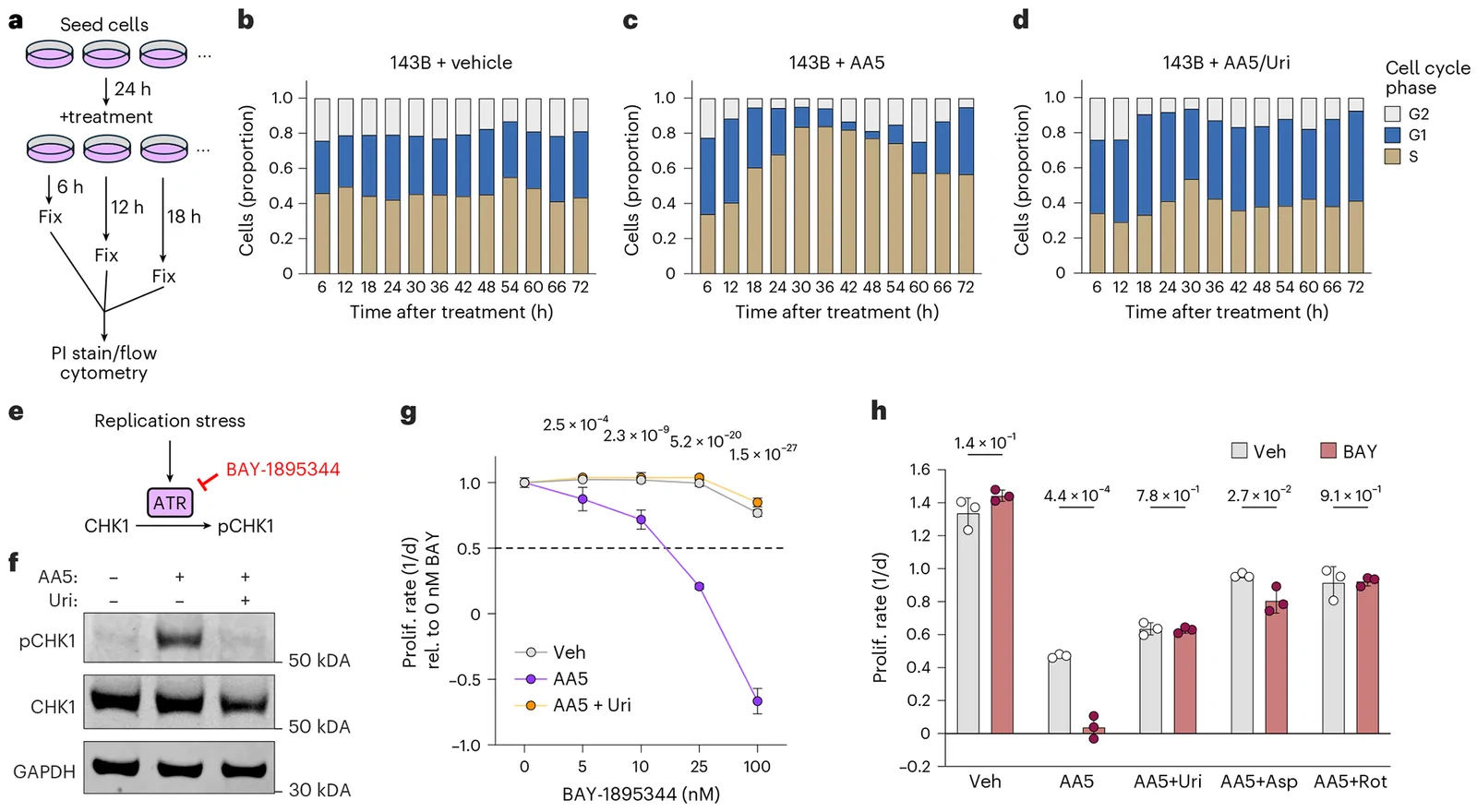
SDH inhibition causes replication stress by inhibiting pyrimidine biosynthesis. **a**, Schematic illustrating the experimental setup for cell cycle analysis of 143B cells treated with AA5 for various time intervals. **b**–**d**, Representative cell cycle experiment showing the proportion of cells in each cell cycle phase in 143B cells treated with vehicle control (**b**), 5 μM AA5 (**c**) or 5 μM AA5 and 200 μM uridine (**d**) at the indicated time points post-treatment (*n* = 1). **e**, Schematic illustrating ATR’s function in sensing replication stress and phosphorylating CHK1 in response. The ATR inhibitor BAY-1895344 (BAY) is indicated. **f**, Representative western blot demonstrating levels of phosphorylated CHK1 (pCHK1), total CHK1 and GAPDH loading control in 143B cells treated with vehicle control, 5 μM AA5 or 5 μM AA5 and 200 μM uridine for 24 h. **g**, Proliferation rates (normalized to 0 nM BAY in each respective condition and measured using a conventional, 72-h end point proliferation assay) of 143B cells treated with vehicle control, 5 μM AA5 or 5 μM AA5 and 200 μM uridine and the indicated doses of BAY (*n* = 3 replicate wells per treatment condition). **h**, Absolute proliferation rates (measured using a conventional, 72-h end point proliferation assay) of 143B cells treated with vehicle control or 20 nM BAY and the indicated combinations of vehicle control, 5 μM AA5, 200 μM uridine (Uri), 20 mM aspartate (Asp) and 50 nM rotenone (Rot) (*n* = 3 replicate wells per treatment condition). Unless otherwise noted, experiments were conducted in DMEM with 1 mM pyruvate. Data are represented as mean ± s.d. Statistical significance determined using an ordinary two-way ANOVA with uncorrected Fisher’s LSD and a single pooled variance (**g**) or multiple two-sided unpaired *t*-tests (**h**). All statistics displayed on graphs represent *P* values unless noted otherwise. In **g**, *P* values denote the results of statistical testing comparing Veh and AA5 conditions at each dose of BAY.

Imbalanced nucleotide availability can cause replication stress, a physiological state characterized by DNA replication stalling and DNA damage that can lead to impaired proliferation or cell death if not mitigated^46–49^. As a prolonged S phase is a typical marker of replication stress^46,50^, we used propidium iodide staining and flow cytometry to characterize the cell cycles of unsynchronized 143B cells at several time points following treatment with AA5 or vehicle control (Fig. 5a). While vehicle-treated cells showed roughly equivalent proportions of cells in G1, S and G2 phases throughout the experiment (Fig. 5b), AA5-treated cells saw a dramatic increase in the proportion cells in S phase from approximately 18 to 36 h post-treatment (Fig. 5c). Notably, the proportion of S phase cells then progressively decreased by 72 h post-treatment (Fig. 5c), a time frame characterized by both aspartate and proliferation rate rebounds (Fig. 1d). This S phase accumulation phenotype was rescued by uridine co-treatment (Fig. 5d), indicating that it results from a pyrimidine deficiency. Indeed, inhibiting pyrimidine synthesis using BRQ also caused a similar S phase accumulation phenotype that was rescuable by uridine (Extended Data Fig. 8a). In contrast to the S phase accumulation of AA5-treated cells, which begin to partially resolve around 48 h post-treatment, BRQ-induced S phase accumulation largely persisted up to 72 h, highlighting the distinction between BRQ’s ‘full block’ of pyrimidine synthesis at DHODH versus a competitive inhibition of pyrimidine synthesis at ATCase by succinate, which may be overcome with sufficient aspartate accumulation. We note that this ‘synchronize and release’ effect following SDH inhibition likely underlies the cell proliferation rebound observed in AA5-treated cells (Fig. 1d).

In cancer cells, cell cycling defects caused by nucleotide imbalance-mediated replication stress can occur without commensurate decreases in growth signalling, resulting in an increase in cell size^49^. Consistent with these studies, we noted that AA5 treatment significantly increases cell size; a phenotype that can be partially rescued with rotenone, re-established with succinate addition and completely rescued by uridine supplementation (Extended Data Fig. 8b). FH-KO cells are similarly sized to their parental cells but swell significantly upon AA5 treatment. This enlargement could be rescued with aspartate or uridine (Extended Data Fig. 8b), supporting increased cell size as a parameter that is phenotypically linked to pyrimidine synthesis and replication stress in these contexts.

To directly evaluate replication stress signalling in response to SDH inhibition, we measured phosphorylation ofthe canonical replication stress-associated checkpoint kinases 1 and 2 (CHK1/2)^51^ (Fig. 5e). As a positive control, we treated 143B cells with excess adenine, which causes replication stress secondary to a purine nucleotide imbalance, leading to CHK1 and subsequent CHK2 phosphorylation over time, as previously reported^49^ (Extended Data Fig. 8c). Notably, AA5 treatment induced CHK1 phosphorylation as early as 24 h post-treatment (Fig. 5f and Extended Data Fig. 8c), and pCHK1 levels were diminished by 72 h, at which point AA5-treated cells begin to overcome S phase arrest (Fig. 5c and Extended Data Fig. 8c). AA5-induced CHK phosphorylation could also be substantially rescued by uridine, aspartate or rotenone, which are metabolically distinct treatments that all converge on alleviating pyrimidine deficiency (Fig. 5f and Extended Data Fig. 8c,d).

ATR signalling is essential during replication stress to prevent a catastrophic loss of DNA integrity and subsequent cell death, quiescence or senescence^46,51,52^. Notably, a recent study found that ATR inhibition synergizes with pyrimidine synthesis inhibition at the DHODH step^53^. Thus, we tested whether SDH impairment would also sensitize cells to the ATR inhibitors BAY-1895344 (54) (BAY) or VE-821 (55), which we verified to block CHK1 phosphorylation (Extended Data Fig. 8e,f). Indeed, while up to 100 nM BAY had little effect on 143B cell proliferation, AA5 treatment dramatically sensitized the cells to this drug, an effect that could be abolished by uridine co-treatment (Fig. 5g). This phenotype was reproduced using VE-821 (Extended Data Fig. 8g), and we also observed pyrimidine-dependent sensitization to BAY upon AA5 treatment in other transformed and non-transformed cell lines (Extended Data Fig. 8h,i). Notably, ATR inhibitor sensitivity is not a general feature of aspartate-limited or slow-growing cells, as rotenone-treated cells in pyruvate-free medium were not sensitized to BAY (Extended Data Fig. 8j). Instead, our data indicate that this synergy is dependent on pyrimidine synthesis impairments, as aspartate, rotenone or uridine treatment were all sufficient to abolish the toxicity of BAY and VE-821 in AA5-treated cells (Fig. 5h and Extended Data Fig. 8g). Collectively, these results suggest a model whereby SDH inhibition-induced succinate accumulation impairs pyrimidine biosynthesis, causing replication stress, S phase arrest and sensitivity to interventions that prevent activation of replication stress signalling.

### SDH loss impairs ATCase activity in cells and tumours

**Extended Data Fig. 9 |:**
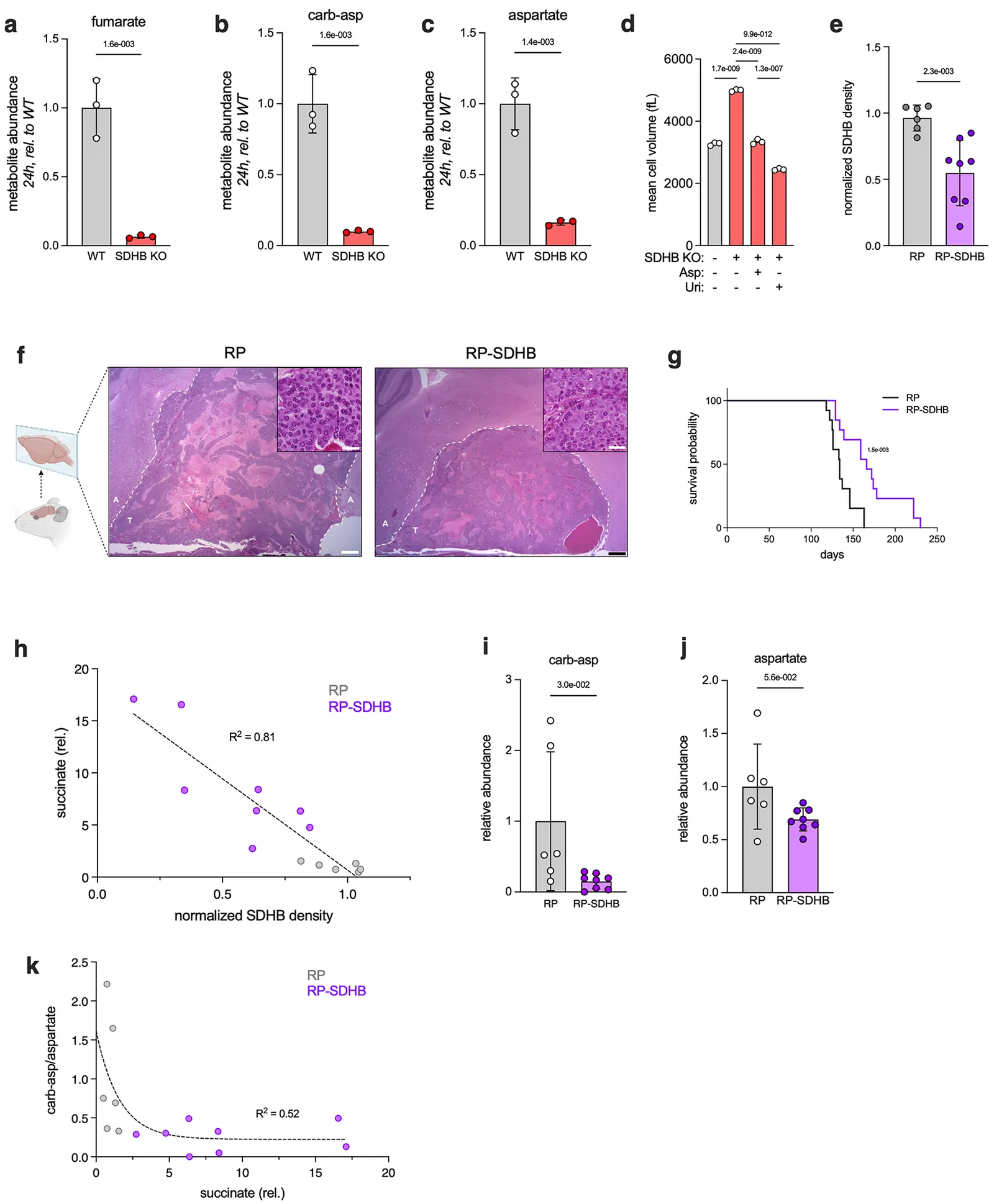
Related to Fig. 6. **a-c**, relative fumarate (a), carbamoylaspartate (b), and aspartate (c) abundances measured by LC–MS on SDHB-KO 143B cells 24 h after media change (*n* = 3 replicate wells per treatment condition). **d** mean cell volumes of wild-type or SDHB-KO 143B cells 48 h after treatment with vehicle control, 20 mM aspartate or 200 μM uridine (*n* = 3 replicate wells per treatment condition). **e**, relative SDHB band density in tumour extracts from *Rb*^−/−^, *Tp53*^−/−^, *Sdhb*^−/−^ (RP-SDHB) or *Rb*^−/−^, *Tp53*^−/−^ mice (RP) calculated from the blot in Fig. 6i (*n* = 6 RP mice, 8 RP-SDHB mice). **f**, representative hematoxylin and eosin (H&E) stained, formalin-fixed tumour sections from one RP and RP-SDHB mouse. Larger images are 2X magnification, insets are 60X. Dotted lines separate tumour (T) from adjacent (A) tissue, 2X scale bar = 600 μm, inset scale bar = 20 μm. **g**, Kaplan-Meier plot demonstrating overall survival of RP-SDHB and RP mice (*n* = 13). **h**, normalized SDHB band density from Fig. 6i plotted against relative succinate abundances from Fig. 6j on a per-tumour basis. Dotted line and R^2^ value show results of linear regression analysis (*n* = 6 RP, 8 RP-SDHB). **i-j**, relative carbamoyl-aspartate (i) and aspartate (j) abundances measured by LC–MS on pituitary tumour extracts from RP and RP-SDHB mice (*n* = 6 RP mice, 8 RP-SDHB mice). **k**, relative succinate abundances from Fig. 6j plotted against carbamoyl-aspartate/aspartate ratios from Fig. 6k on a per-sample basis. Dotted line and R^2^ value show the results of nonlinear regression analysis fitting a one-phase exponential decay (*n* = 6 RP mice, 8 RP-SDHB mice). Unless otherwise noted, experiments were conducted in DMEM with 1 mM pyruvate, data represented as median +/− quartiles (panels **h**-**i**) or mean +/− S.D. (all other panels). Statistical significance determined using two-sided unpaired t-tests (panels **a**-**c**, **e**, **i**-**j**) Mantel-Cox test (panel **g**) or an ordinary one-way ANOVA with uncorrected Fisher’s LSD and a single pooled variance (panel **d**). All statistics displayed on graphs represent *P* values unless noted otherwise.

**Fig. 6 |:**
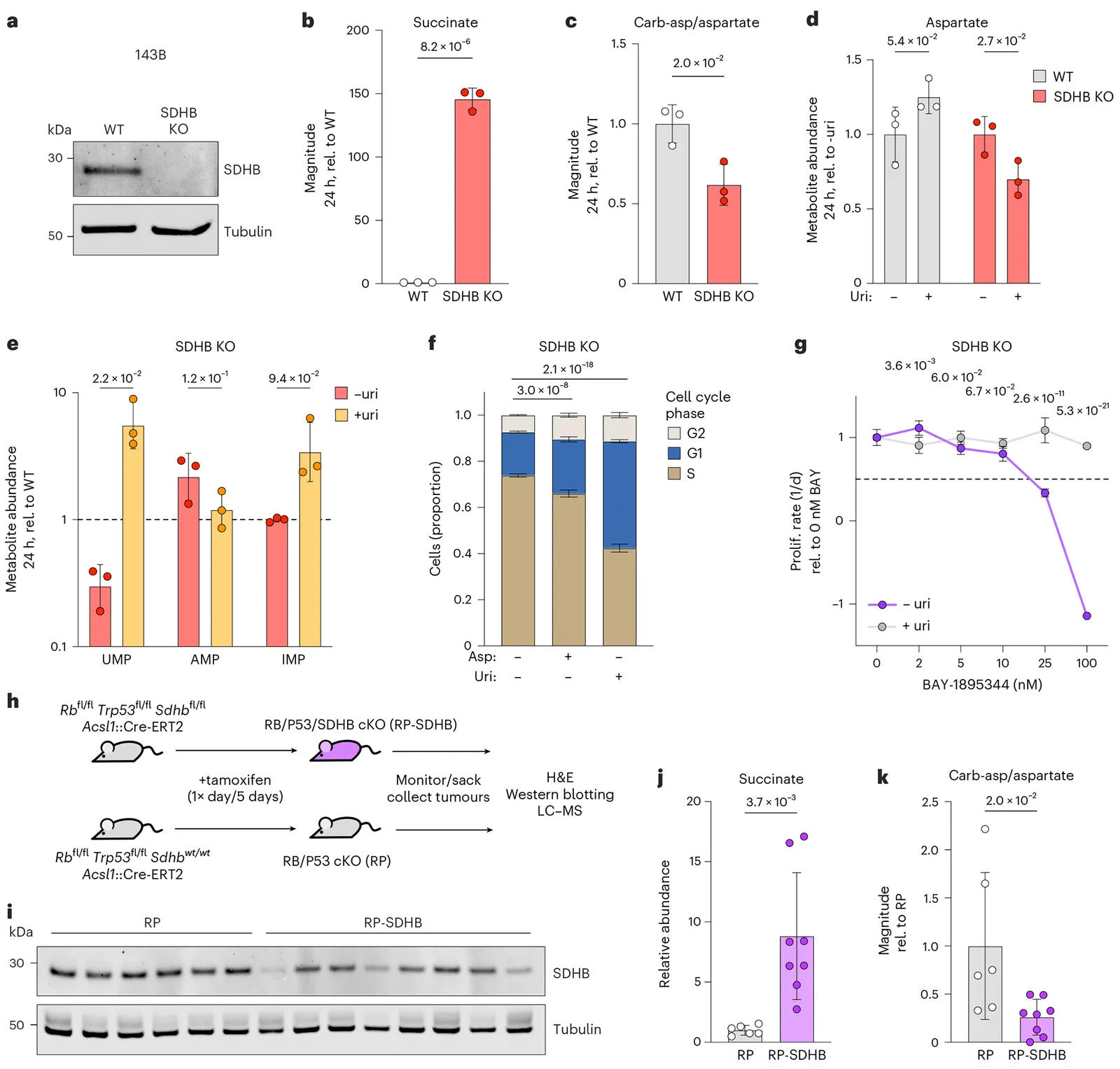
SDH loss impairs ATCase activity in cells and tumours. **a**, Representative western blot demonstrating levels of SDHB and tubulin loading control in SDHB-KO 143B cells. **b**, Relative succinate abundances measured by LC–MS on SDHB-KO 143B cells 24 h after medium change (*n* = 3 replicate wells per treatment condition). **c**, Relative carbamoyl-aspartate:aspartate ratio measured by LC–MS in SDHB-KO 143B cells 24 h after medium change (*n* = 3 replicate wells per treatment condition). **d**, Relative aspartate abundances (normalized to untreated condition for each cell line) measured by LC–MS on WT and SDHB-KO 143B cells 24 h after treatment with vehicle control or 200 μM uridine (*n* = 3 replicate wells per treatment condition). **e**, Relative abundances of UMP, AMP and IMP measured by LC–MS on SDHB-KO 143B cells 24 h after treatment with vehicle control or 200 μM uridine (*n* = 3 replicate wells per treatment condition). Abundances are normalized to those of WT 143B cells in the same experiment, which is represented by the dashed line at *y* = 1. **f**, Representative cell cycle experiment showing the proportion of SDHB-KO 143B cells in each cell cycle phase 24 h after treatment with vehicle control, 20 mM aspartate or 200 μM uridine (*n* = 3 replicate wells per treatment condition). **g**, Proliferation rates (normalized to 0 nM BAY in each respective condition and measured using a conventional, 72-h end point proliferation assay) of SDHB-KO 143B cells treated with vehicle control or 200 μM uridine and the indicated doses of BAY (*n* = 3 replicate wells per treatment condition). **h**, Schematic illustrating the design and experimental layout of the *Rb*^−/−^, *Tp53*^−/−^, *Sdhb*^−/−^ (RP-SDHB) mouse model. **i**, Western blot demonstrating levels of SDHB and tubulin loading control in individual *Rb*^−/−^, *Tp53*^−/−^, *Sdhb*^−/−^ (RP-SDHB) and littermate control (*Rb*^−/−^, *Tp53*^−/−^, RP) pituitary tumour extracts (*n* = 6 RP mice, 8 RP-SDHB mice). **j**, Relative succinate levels measured using LC–MS on RP and RP-SDHB tumour extracts in **i** (*n* = 6 RP mice, 8 RP-SDHB mice). **k**, Relative carbamoyl-aspartate/aspartate ratio measured using LC–MS on RP and RP-SDHB tumour extracts in **i** (*n* = 6 RP mice, *n* = 8 RP-SDHB mice). Unless otherwise noted, experiments were conducted in DMEM with 1 mM pyruvate; data are represented as mean ± s.d. Statistical significance determined using two-sided unpaired *t*-tests (**b**–**e**,**j**,**k**) or an ordinary two-way ANOVA with uncorrected Fisher’s LSD and a single pooled variance (**f**,**g**). All statistics are displayed on graphs represent *P* values unless noted otherwise. In **f**, *P* values denote results of statistical testing comparing the S phase fraction in the indicated treatment conditions.

To rule out any potential off-target effects of AA5 and test the effects of chronic SDH loss on pyrimidine synthesis, we used CRISPR/Cas9 to generate clonal SDHB-KO 143B cells^7^ (Fig. 6a) that show depletion of fumarate and accumulation of succinate compared with parental cells, in accordance with impaired SDH activity (Fig. 6b and Extended Data Fig. 9a). In this system of chronic SDH impairment, there is no notion of an aspartate rebound. Instead, we would expect SDH-KO cells to exist in a ‘post-rebound’ state of low ATCase activity, impaired pyrimidine synthesis and persistent replication stress. Indeed, SDH-KO cells have a lower carbamoyl-asp/aspartate ratio and decreased UMP levels compared with parental controls, consistent with ATCase impairment and resulting pyrimidine deficiency (Fig. 6c,e and Extended Data Fig. 9b,c).

Given our previous results, we would also predict that supplementing uridine to pyrimidine-limited SDH-KO cells would reengage aspartate consumption, thereby depleting aspartate levels until they became limiting for purine synthesis (Extended Data Fig. 5a,d). Consistent with this model, uridine supplementation significantly lowers aspartate levels in SDH-KO cells but not parental controls (Fig. 6d) and uridine-treated SDH-KO cells show evidence of emergent AMP deficiency and IMP accumulation (Fig. 6e). Next, we analysed cell size, cell cycling, and ATRi sensitivity to determine whether permanent SDH loss results in persistent replication stress. Notably, SDH-KO cells show significantly larger cell volumes than parental controls, an increased proportion of cells in S phase, and sensitivity to BAY (Fig. 6f,g and Extended Data Fig. 9d). All three of these phenotypes could be partially or fully rescued by aspartate or uridine, supporting the hypothesis that genetic SDH loss causes replication stress via the mechanisms outlined above.

Finally, we sought to test the relationship between SDH function and ATCase activity in a more physiological setting. SDH subunits have been implicated as tumour suppressors for several neuroendocrine tumour types, including pituitary adenomas^56–63^. To this end, we generated mice in which the tumour suppressors *Rb*, *Trp53* and *Sdhb* are simultaneously knocked out using tamoxifen-inducible Cre-ERT2 under the neuroendocrine-specific *Ascl1* promoter (Fig. 6h). These mice reproducibly form pituitary adenomas over several months following tamoxifen induction, although SDHB loss did not produce a more aggressive disease in this system (Extended Data Fig. 9f,g).

We collected tumours at the end point and analysed samples by both western blotting and LC–MS to examine SDH loss and its effects on metabolic state (Fig. 6h). Compared to pituitary tumours from *Rb*- and *Trp53*-null (RP) littermate controls, tumours from *Rb*-, *Trp53*- and *Sdhb*-null (RP-SDHB) mice showed substantially lower, but not uniformly absent, SDHB protein levels, likely reflecting some mosaicism in the resulting tumours (Fig. 6i and Extended Data Fig. 9e). Nonetheless, RP-SDHB tumours collectively showed higher succinate levels than RP controls (Fig. 6j), consistent with impaired SDH activity, which anticorrelated with SDHB protein abundance on a per-sample basis (Fig. 6j and Extended Data Fig. 9h). Next, we examined the carbamoyl-aspartate/aspartate ratio as a metabolic indicator of ATCase activity. Consistent with SDH loss inhibiting ATCase activity, the carbamoyl-aspartate/aspartate ratio was significantly lower in RP-SDHB tumours compared with RP controls (Fig. 6k and Extended Data Fig. 9i,j). On a per-sample basis, carb-asp/aspartate and succinate showed an exponential relationship, consistent with a threshold of succinate abundance above which ATCase activity becomes impaired (Extended Data Fig. 9k).

**Fig. 7 |:**
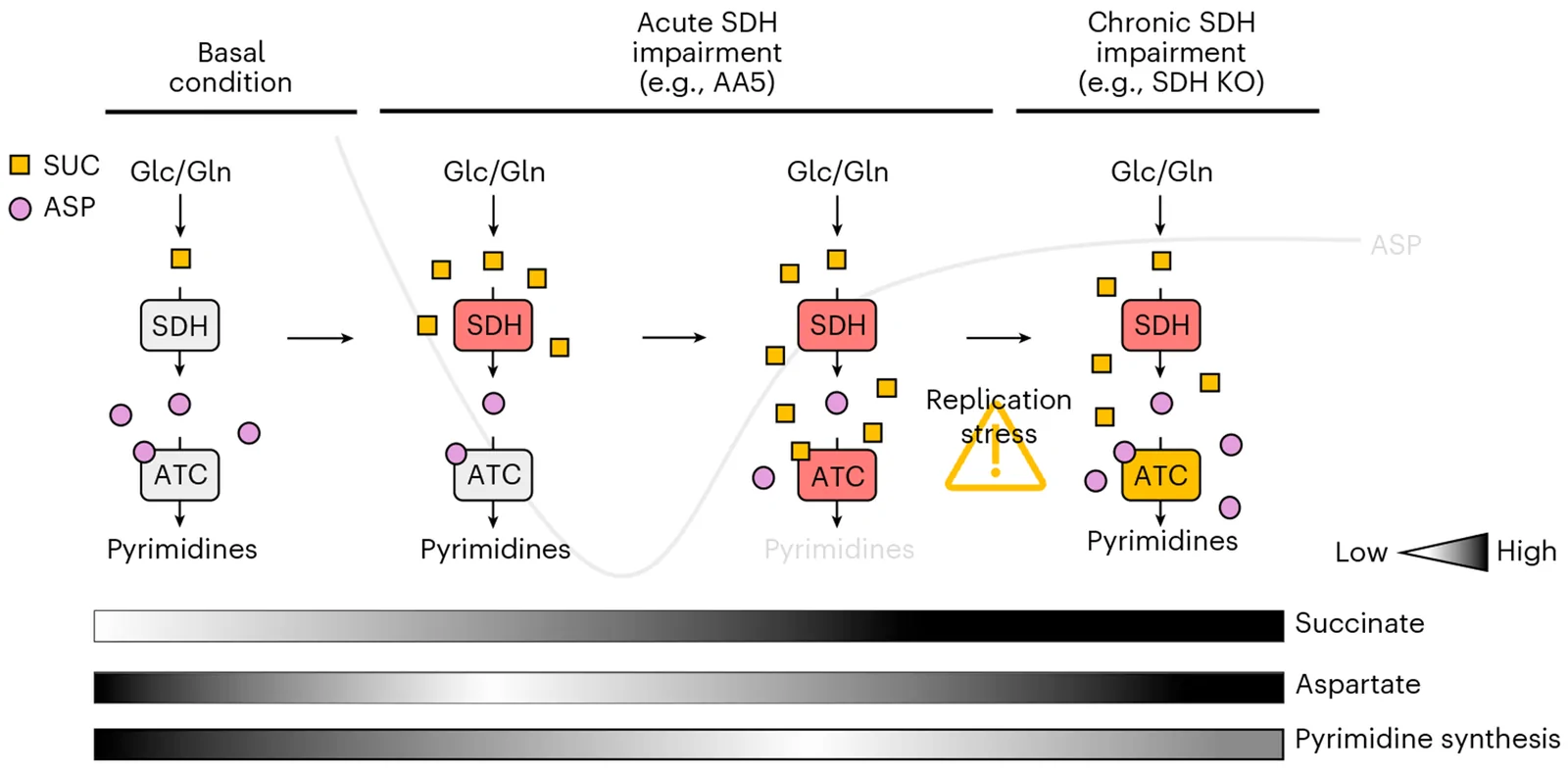
Model depicting the interaction between SDH status, metabolite levels and ATCase activity. SDH inhibition acutely decreases aspartate abundance and causes accumulation of succinate, which competitively impairs aspartate utilization for pyrimidine synthesis at ATCase. The resulting pyrimidine deficiency then disproportionately decreases aspartate consumption, allowing aspartate to accumulate until it outcompetes succinate, re-engaging pyrimidine synthesis. Cells with chronic SDH deficiency experience persistent pyrimidine synthesis impairments and replication stress at equilibrium, as aspartate levels stabilize at the threshold of limitation for ATCase function.

Overall, our results suggest a cohesive model describing the interaction between SDH activity, pyrimidine synthesis and aspartate abundance under both and acute and chronic settings ofSDH impairment (Fig. 7). Under basal cellular conditions, both SDH and ATCase are active, aspartate levels are high and succinate pools are low. Acute SDH impairment causes an immediate decrease in aspartate acquisition (production), while proliferation impairments lag metabolic changes, leading to a net decrease in aspartate levels. At some point, aspartate levels decrease and succinate accumulates enough to inhibit pyrimidine synthesis at ATCase, leading to pyrimidine deficiency, replication stress, and S phase accumulation. In this regime, overall aspartate consumption dips even lower than its acquisition, leading to an aspartate rebound. As aspartate levels increase, however, they once again become competitive with succinate, licensing some ATCase activity/pyrimidine synthesis and temporarily releasing the cells from S phase. Cells now exist in a regime in which aspartate levels are kept ‘artificially’ high due to succinate-mediated ATCase inhibition: aspartate levels rise until ATCase is sufficiently disinhibited to support proliferation, after which aspartate is consumed and once again falls below the threshold needed to sustain ATCase activity, pyrimidine synthesis and proliferation. Cells exhibiting chronic SDH impairment exist permanently in this last regime, characterized by persistent replication stress and slow cell cycling (Fig. 7).

## Discussion

Here, we leverage an aspartate biosensor and live-cell imaging to exemplify a model in which cellular aspartate abundance dynamics are determined by its acquisition and consumption. The variable aspartate behaviours found under different metabolic contexts observed here also reinforce the principle that metabolite levels neither directly equate to pathway flux nor inherently identify how metabolic perturbations impact cell function, two common misconceptions in metabolism research^64^. In the case of aspartate limitation, we reveal that aspartate depletion causes functional effects once aspartate becomes limiting for the synthesis of specific anabolic fates. For SDH inhibition, we find a hierarchy of deficiencies for synthesis of pyrimidines, then purines, then proteogenic fates. Overall, these results highlight the power of non-destructive, temporally resolved measurements to capture complex metabolic dynamics that are missed by traditional metabolomics measurements conducted at limited time points.

Our mechanistic investigation of the metabolic consequences following SDH deficiency reveals a role for succinate as a modulator of mammalian pyrimidine biosynthesis at ATCase. While aspartate is a substrate for both purine and pyrimidine biosynthesis (both of which are required for cell proliferation) we propose that succinate-mediated ATCase inhibition leads to a disproportionate effect on pyrimidine synthesis, which emerges as the superseding metabolic defect following SDH loss in contexts where pyrimidines cannot otherwise be salvaged. As succinate accumulation is observed in physiological contexts beyond SDH-mutant cancers, including hypoxia, ischaemia/reperfusion, liver inflammation, macrophage function and brown adipose tissue thermogenesis^31,65–76^, it will be important to consider the potential roles that pyrimidine synthesis impairments play in these contexts, which may also depend on the availability of salvageable nucleotide precursors in the microenvironment^77–79^. Finally, our findings in SDH-KO cells and tumours raise the question of whether SDH-mutant tumours may be sensitive to interventions targeting the consequences of pyrimidine deficiency, including replication stress. Consistent with this possibility, several studies using preclinical models of SDH-deficient cancers have found evidence of alterations to nucleotide homeostasis and genomic integrity^32,80–82^. Overall, this work expands the growing understanding of how metabolites can regulate cell function beyond their direct roles as catabolic or anabolic substrates.

## Methods

### Cell culture

Cell lines (143B, H1299, HCT116, 293T pLentiX and HT1080) were acquired from ATCC, authenticated using short tandem repeat profiling, and regularly tested to be free from *Mycoplasma* contamination (MycoProbe, R&D Systems). Cells were maintained in DMEM (Gibco, 50-003-PB) supplemented with 3.7 g l^−1^ sodium bicarbonate (Sigma-Aldrich, S6297), 10% fetal bovine serum (FBS) (Gibco, 26140079) and 1% penicillin–streptomycin solution (Sigma-Aldrich, P4333). Cells were cultured in a humidified incubator at 37 °C with 5% CO_2_.

### Generation of jAspSnFR3/NucRFP cell lines

Nuclear RFP cell lines were generated as previously described^22^. jAspSnFR3 lentivirus was generated by co-transfection of HEK293T pLentiX cells with p-Lenti-jAspSnFR3 (Addgene, 203488) and the packaging plasmids pMDLg/pRRE (Addgene, 12251), pRSV-Rev, (Addgene, 12253) and pMD2.G (Addgene, 12259) using FuGENE transfection reagent (Fisher, PRE2693) in DMEM (Fisher, MT10017CV) without FBS or penicillin–streptomycin. The supernatant containing lentiviral particles was filtered through a 0.45-μm membrane (Fisher, 9720514) and was supplemented with 8 μg μl^−1^ Polybrene (Sigma, TR-1003-G) before infection. For infection, 143B and GOT1/2 DKO 143B cells were seeded at 50% confluency in six-well dishes and centrifuged with lentivirus (900 g, 90 min, 30 °C). After 24 h the medium was replaced and after 48 h cells were treated with 150 μg ml^−1^ hygromycin (Sigma-Aldrich, H7772-1G) and maintained in selection medium until all uninfected control cells died. After selection, cells were expanded and single-cell cloned by limiting dilution, plating 0.5 cells per well using two 96-well plates. These clones were incubated until 10–30% confluency and screened for high GFP and RFP signal using an Incucyte S3 (Sartorius). The highest expressing monoclonal cells were selected and further expanded on six-well plates and again screened for high fluorescence using the Incucyte. From this, single clones were chosen, expanded and used for all subsequent experiments. H1299 and GOT1/2 DKO H1299 cells expressing jAspSnFR3 and nuclear RFP were previously generated^22^, whereas wild-type (WT) 143B, GOT DKO 143B, and cells were engineered to express nuclear RFP (Cellomics, PLV-10205-50) and pLenti-jAspSnFR3 for this paper. GOT1/2 DKO 143B and H1299 cells (no aspartate sensor) with and without SLC1A3 expression were also previously generated^22^.

### jAspSnFR3 and NucRFP Incucyte measurements

Experiments were conducted in either DMEM without pyruvate (Corning 50-013-PB) or DMEM with 1 mM pyruvate (Sigma, P8574) as indicated in the figure legends, supplemented with 3.7 g l^−1^ sodium bicarbonate, 10% dialysed FBS (Sigma-Aldrich, F0392) and 1% penicillin–streptomycin solution. To start an experiment, cells were trypsinized (Thermo Fisher, 25200056), resuspended in medium, counted using a Coulter Counter (Beckman Coulter, Multisizer 4) and seeded onto 24-well dishes (Nunc, 142475) with an initial seeding density of 15,000, 18,000, 50,000 or 20,000 cells per well for H1299, 143B, H1299/143B GOT1/2 DKO, respectively. After 24 h of incubation, the plates were moved into an Incucyte S3 (Sartorius) live-cell imaging platform inside a humidified incubator at 37 °C with 5% CO_2_ for a pre-treatment scan. Once the scan was complete, plates were removed for treatment. Drug treatments such as Atpenin A5 (MedChemExpress, HY-126653) and Rotenone (Sigma-Aldrich, R8875) were spiked in as 2× solutions in DMEM without pyruvate and dialysed FBS along with 2× sodium pyruvate, where applicable. For treatments with varying medium aspartate (Sigma-Aldrich, A7219) wells were washed with phosphate-buffered saline (PBS) and filled with DMEM containing the various aspartate concentrations. For plates receiving asparagine (Sigma-Aldrich, A7094) or uridine (Sigma-Aldrich, U3003), stocks were generated in water and made into 2× stocks in medium, before a final dilution into experimental medium so that the final concentration was 500 μM (Asn) or 200 μM (Uri), with vehicle wells receiving an equivalent volume of medium with water in the place of the metabolite additives. Adenine (Sigma, A2786) was prepared fresh for each experiment by dissolving powder in 500 μl 1 M HCl, neutralizing with 500 μl 1 M NaOH and filtering through a 0.22-μm filter (Fisher, FB12566506). This solution was then added to fresh medium so that the final concentration was 100 μM. Live-cell imaging was performed on the Incucyte S3 using the GFP and RFP channels with default exposure times. Images were processed using the associated Incucyte software to subtract background, define areas of cell confluence and GFP:RFP signal and extract integrated GFP:RFP values per well. Reported GFP:RFP values were normalized to the pre-treatment scan on a per-well basis. Nuclei were counted as RFP instances at each time point, and average proliferation rates were calculated in 7–9-h sliding windows according to the formula:

$$\text{Proliferation rate}=\frac{\log_{2}(\frac{{\text{nuclei}}_{t_{2}}}{{\text{nuclei}}_{t_{1}}})}{t_{2}-t_{1}}$$

where *t*_1_ and *t*_2_ are initial and final scan times measured in days, respectively, and proliferation rate is calculated as cell doublings per day. Aspartate rebound was quantified by dividing the final normalized GFP:RFP value with the minimum normalized GFP:RFP value during an assay, per well.

### Conventional proliferation assays and cell volume measurements

Cells were trypsinized, resuspended in medium and counted (Beckman Coulter Counter Multisizer 4 or Nexcelom Auto T4 Cell-o-meter) and seeded overnight onto 24-well dishes (Corning, 3516) with similar initial seeding densities described above. After overnight incubation, three wells were counted for a starting cell count at the time of treatment. Cells were washed in PBS and 1–2 ml of the treatment medium was added to each well. Experiments were conducted in DMEM without pyruvate supplemented with 3.7 g l^−1^ sodium bicarbonate 10% dialysed FBS and 1% penicillin–streptomycin solution, with or without 1 mM sodium pyruvate, 20 mM aspartate or 0.5 mM asparagine, 200 μM uridine, 100 μM adenine or 10–25 mM succinic acid (Sigma, S3674). Drug treatments included rotenone (Sigma-Aldrich, R8875), cycloheximide (Sigma, C7698), Atpenin A5 (MedChemExpress, HY-126653), BAY-1895344 (Selleckchem, S8666) and dimethylsulfoxide (DMSO) as a vehicle (Sigma, D2650). Cells were incubated in a humidified incubator at 37 °C with 5% CO_2_ then counted after 2–4 days. At the end point, cells were counted using a Beckman Coulter Counter Multisizer 4 instrument, which measures individual particle (cell) volumes in addition to counting cells. The proliferation rate was determined as described previously^7^.

### Generation of KO cells

Knockout cell lines were generated as previously described^7,22^. Three single-guide RNA (sgRNA) sequences targeting FH were purchased (Synthego) (Supplementary Table 1). Each sgRNA was resuspended in nuclease-free water, combined with SF buffer (Lonza, V4XC-2032) and sNLS-spCas9 (Aldevron, 9212). Then, 2 × 10^5^ 143B cells were resuspended in the resulting solution containing ribonucleoprotein complexes (RNPs) and electroporated using a 4D-Nucleofector (Amaxa, Lonza) using electroporation programme FP-133. Nucleofected cells were then moved to a 12-well plate (Corning, 3513) and, after achieving confluence, were single-cell cloned by limiting dilution by plating 0.5 cells per well in a 96-well plate. Gene KO was confirmed using western blots on the nucleofected pool and each single-cell clone used in this study. GOT1/2 DKO cells were generated in the same way and were previously described^22^.

### Western blotting

Protein lysates were collected in RIPA buffer (Sigma, R0278) supplemented with protease inhibitors (Fisher, A32953) and phosphatase inhibitors (Fisher, 78442). Protein concentration was determined using a Bicinchoninic Acid Assay (Fisher, 23225) using bovine serum albumin (BSA) as a protein standard. Equal amounts of protein were denatured with Bolt 4× Loading Dye (Thermo Fisher, B0007) and Bolt 10× reducing agent (Thermo Fisher, B0004), heated at 95 °C for 2–5 min and loaded onto 4–12% by SDS–polyacrylamide gels (Invitrogen, NW04127). After electrophoretic separation, proteins were transferred onto a 0.22-mm nitrocellulose membrane using iBlot2 transfer stacks (Fisher, IB23001) and transferred with the P0 system setting. Membranes were blocked with 5% BSA in Tris-buffered saline with 0.1% Tween-20 (TBS-T) and incubated at 4 °C overnight with the following antibodies: anti-GOT2 (Proteintech, 14800-1-AP, 1:1,000 dilution), anti-GOT1 (Cell Signalling, 34423S, 1:1,000 dilution), anti-GFP (Sigma, 11814460001, 1:1,000 dilution), anti-FH (Origene, TA500675S, 1:1,000 dilution), anti-SDHB (Atlas, HPA002868, 1:1,000 dilution), anti-pChk1 (Cell Signalling, 2348S, 1:1,000 dilution), anti-Chk1 (Cell Signalling, 2G1D5, 1:1,000 dilution), anti-pChk2 (Cell Signalling, 2197S, 1:1,000 dilution), anti-GAPDH (Cell Signalling, 5174S, 1:5,000 dilution), anti-Vinculin (Sigma, SAB4200729, 1:10,000 dilution) and anti-tubulin (Sigma, T6199). The next morning, membranes were washed three times with TBS-T and the following secondary antibodies were added: 800CW goat anti-mouse IgG (LiCOR, 926-32210; 1:15,000 dilution) and 680RD goat anti-rabbit IgG (LiCOR, 926-68071; 1:15,000 dilution). Membranes were washed three more times with TBS-T and imaged on a LiCOR Odyssey Near-Infrared imaging system.

### RNA extractions and tRNA aminoacylation quantification

143B cells were grown on six-well plates in DMEM without pyruvate, in dialysed FBS. For the Atpenin A5 treatment, 1 mM pyruvate was added to the medium. At 50% confluency, cells were treated with drugs in replicates for 30 h. 143B cells were treated with vehicle (DMSO), rotenone (50 nM) or Atpenin A5 (5 μM). For RNA extraction, the medium on the cells was quickly and thoroughly aspirated before adding 3 ml TRIzol (Thermo Fisher, A33250) to cover all the cells. From this point onward, everything was kept ice-cold to prevent hydrolysis of the aminoacylation. After a 2-min incubation, the cell material was scraped down the slope mixing it with the TRIzol, then 2 × 1.5 ml was transferred to 2-ml Eppendorf tubes and 0.3 ml chloroform was added. The tubes were vortexed 2 min and then centrifuged (17,000*g*, 5 min). From each tube, 0.75 ml of the upper layer was transferred to a tube with 0.8 ml isopropanol (IPA) (Thermo Fisher, A464SK), then mixed and incubated 60 min at −20 °C. Tubes were then centrifuged (17,000*g*, 15 min) and RNA pellets were washed twice with 1 ml 80% IPA containing 100 mM sodium acetate (pH 4.5) (Sigma, S7545). Washes are critical to prevent glycerol present in TRIzol from inhibiting the subsequent periodate oxidation step. A last wash was performed using 1 ml 100% IPA and after removing the supernatant the RNA pellets were air-dried at room temperature, then stored dry at −80 °C. Aminoacylation levels, referred to as ‘charge’, were measured by sequencing and determining the fraction of tRNAs protected from periodate oxidation and 3’ nucleotide cleavage, as previously described in detail^24^. The values for each of the multiple tRNA transcripts decoding each codon were calculated as an expression weighted average of all codon-specific transcript charges.

### Polar metabolite extractions

#### Time course.

For LC–MS measurements across several time points, 143B cells were seeded overnight at either 2 × 10^5^, 1 × 10^5^ or 0.5 × 10^5^ cells per well of a six-well dish for the 10/16-h, 24-h and 44-h time points, respectively. The next day, cells were washed twice with PBS and changed to the indicated medium supplemented with 1 mM pyruvate, 10% dialysed FBS, 1% penicillin–streptomycin and treatments as indicated, and returned to the tissue culture incubator. Polar metabolites were extracted from cells by three washes with ice-cold blood bank saline (Fisher, 23293184) then 300 μl of 80% HPLC-grade methanol in HPLC-grade water was added to each well and cells were scraped with the back of a P1000 pipette tip and transferred to Eppendorf tubes. Tubes were centrifuged (17,000*g*, 15 min, 4 °C) and the supernatant containing polar metabolites was transferred to a new centrifuge tube and placed in a Centrivap until lyophilized. Corresponding plates with the same treatment conditions were trypsinized at the same time metabolites were extracted and counted on a Beckman Coulter Counter to obtain total cell volume per well. Average cell volumes for each condition were calculated and used to normalize metabolites after centrifugation. Dried metabolites were resuspended in 40 μl solvent per μl cell volume in 80% HPLC-grade methanol containing ^13^C-labelled metabolites made by partial hydrolysis (12 h in 6 M HCl at 90 °C) of U-^13^C spirulina whole cells lyophilized powder (Cambridge Isotope Laboratories, CLM-8400-PK) as an internal standard to account for matrix effects and absolute quantitation of intracellular metabolites. Ion counts were normalized to the internal standard metabolite when appropriate to determine a response ratio.

#### Succinate and aspartate concentration measurements.

A standard curve of known succinate and aspartate concentrations over three orders of magnitude was generated in 80% HPLC-grade methanol containing ^13^C spirulina standard and run in parallel with the time-course experiment detailed above. Response ratios for succinate and aspartate ion counts from cell extracts were used to calculate a concentration according to the standard curve, which were then back calculated by cell volume to generate intracellular concentrations.

#### Other measurements.

For standard metabolic analysis, cells were seeded overnight at 1 × 10^5^ cells per well of a six-well dish. The next day, cells were washed twice with PBS and changed to the indicated medium supplemented with 10% dialysed FBS, 1% penicillin–streptomycin and treatments as indicated and returned to the tissue culture incubator. After 30–32 h, polar metabolites were extracted from cells by the same protocol as mentioned above.

### Isotope tracing

#### ^15^N Glutamine tracing.

The fractional contribution of individual components into their respective aspartate consuming fate was determined in 143B and H1299 cells for the salvageable metabolites asparagine (Asn), uridine (Uri), adenine (Ade), hypoxanthine (Hpx) (Cayman, 22254) and guanine (Gua) (Sigma, G11950), along with a vehicle treatment (Vec). The salvageable metabolites were spiked in from a 20× stock solution to achieve a final concentration of 500 μM Asn, 200 μM Uri, 100 μM Hpx, 100 μM Ade or 100 μM Gua. The fraction of salvage was determined by stable isotope tracing, performed using both Gln amide-^15^N (Cambridge Isotope Laboratories, NLM-557-PK) and Gln α-^15^N (Cambridge Isotope Laboratories, NLM-1016-PK) in separate reactions and added to DMEM without glucose, glutamine, pyruvate and phenol red (Sigma, D5030) supplemented with 10% dialysed FBS, 1× penicillin–streptomycin and 25 mM glucose (Sigma, G7528). This combination of cell lines, salvageable metabolites, and tracers gave 24 conditions that were labelled to steady state by culturing for four passages with a 1:20 split at each passage. At the end of the last passage each condition was split into four technical replicates and plated onto 24-well plates. Upon reaching confluency, polar metabolites were extracted and submitted to LC–MS. The relative contribution of guanine salvage into the GTP pool was determined using the m + 0 versus m + 3 GTP isotopologue fractions from the Gln amide-^15^N labelled samples. The relative contribution of both adenine and hypoxanthine salvage into the ATP pool was determined using the m + 0 ATP isotopologue fraction from the Gln amide-^15^N labelled samples and the direct contribution from adenine was determined using the m + 1 isotopologue fractions of aspartate compared with ATP from the Gln α-^15^N labelled samples.

#### U-^13^C Glutamine tracing.

WT 143B cells were seeded at 1 × 10^5^ cells per well of a six-well dish. The next morning, cells were washed twice with PBS and changed to DMEM without glucose, glutamine, pyruvate or phenol red (Sigma, D5030) supplemented with 10% dialysed FBS, 1% penicillin–streptomycin, 1 mM pyruvate, 25 mM ^12^C glucose (Sigma, G7528) and 4 mM U-^13^C glutamine (Cambridge Isotopes, CLM-1822). 143B cells were treated as indicated for 32 h, then collected by the same protocol detailed above.

### Liquid chromatography–mass spectrometry

Resuspended samples were transferred to LC–MS vials for measurement by LC–MS. Metabolite quantitation was performed using a Q Exactive HF-X Hybrid Quadrupole-Orbitrap Mass Spectrometer equipped with an Ion Max API source and H-ESI II probe, coupled to a Vanquish Flex Binary UHPLC system (Thermo Scientific). Mass calibrations were completed at a minimum of every 5 days in both the positive and negative polarity modes using LTQ Velos ESI Calibration Solution (Pierce). Polar Samples were chromatographically separated by injecting a sample volume of 1 μl into a SeQuant ZIC-pHILIC Polymeric column (2.1 × 150 mm, 5 mM, EMD Millipore). The flow rate was set to 150 μl min^−1^, the autosampler temperature set to 10 °C and column temperature set to 30 °C. Mobile Phase A consisted of 20 mM ammonium carbonate and 0.1% (v/v) ammonium hydroxide and Mobile Phase B consisted of 100% acetonitrile. The sample was gradient eluted (%B) from the column as follows: 0–20 min: linear gradient from 85% to 20% B; 20–24 min: hold at 20% B; 24–24.5 min: linear gradient from 20% to 85% B; 24.5 min–end: hold at 85% B until equilibrated with ten column volumes. Mobile phase was directed into the ion source with the following parameters: sheath gas = 45, auxiliary gas = 15, sweep gas = 2, spray voltage = 2.9 kV in the negative mode or 3.5 kV in the positive mode, capillary temperature = 300 °C, RF level = 40%, auxiliary gas heater temperature = 325 °C. Mass detection was conducted with a resolution of 240,000 in full-scan mode, with an AGC target of 3,000,000 and maximum injection time of 250 ms. Metabolites were detected over a mass range of 70–1,050 *m/z*. Quantitation of all metabolites was performed using Tracefinder v.4.1 (Thermo Scientific) referencing an in-house metabolite standards library using ≤5 ppm mass error. Data from stable isotope labelling experiments include correction for natural isotope abundance using IcoCor software v.2.2.

For experiments in Extended Data Fig. 7a–d, polar metabolite extracts were generated as described above and resuspended in HPLC-grade 80% methanol without stable isotope standards. Metabolite quantitation was performed using an Agilent 6495D Triple-Quadrupole Mass Spectrometer equipped with an Agilent Jet-Stream Heated ESI source coupled to an Agilent 1290 Infinity II UHPLC (Agilent Technologies). A Checktune was performed using the Agilent ESI-L low concentration tuning mix to assess the status of the instrument before data collection. Polar Samples were chromatographically separated by loading a sample volume of 2 μl onto one of two Poroshell 120 HILIC-Z columns (2.1 × 150mm, 2.7 μm, Agilent Technologies) running in a bespoke alternating column regeneration method. The flow rate was set to 600 μl min^−1^, the multisampler temperature set to 4 °C and column temperature set to 25 °C. Mobile phase A consisted of 0.1% formic acid with 10 mM ammonium formate and mobile phase B consisted of acetonitrile with formic acid at 0.1% (*v*/*v*). The sample was gradient eluted (%B) from the column as follows: 0–0.14 min, initial hold at 95% ‘B’; 0.14–2.29 min, 95% to 40% ‘B’; 2.29–4.0 min and hold at 40% ‘B’. At the end of the method, the column oven valve would switch over to the other (K’ value matched) HILIC-Z column and the gradient would run on column 2, while column 1 was regenerated at 40% to 95% ‘B’ from 0–0.56 min, followed by an increase in flow rate to 1,200 μl min^−1^ and held at 95% ‘B’ until ten column volumes were pumped through the column. Samples were analysed on the mass spectrometer using the following parameters: gas flow = 13.0 l min^−1^, sheath gas = 11 l min^−1^, nebulizer = 35 psi, gas temperature = 200 C, sheath gas temperature = 250 C, capillary voltage = 3,000 V, nozzle voltage = 1,500 V and a CAV voltage of 5 V. Metabolites were targeted in the multiple reaction monitoring mode with a dwell time of20 ms at unit resolution; compound collision energies were previously optimized in the automated mode of MassHunter’s built in optimizer module (v.12.1). Quantitation of all metabolites was performed using MassHunter’s Quantitative Analysis module referencing an in-house metabolite standards library.

### Molecular docking studies

Molecular docking was performed on the Fred Hutchinson Cancer Center high performance computing cluster using Autodock Vina v.1.2.7 (83,84) using the vina scoring function and exhaustiveness of 64. Receptors were prepared using ChimeraX v.1.10.1 and the OpenBabel web server^84^ and docking was performed in a 15 × 15 × 15 Å grid box centred on a single CP/aspartate binding pocket with grid space of 0.375. Docking results were ranked and visualized using ChimeraX v.1.10.1.

### ATCase expression and purification

The ATCase domain of human CAD was expressed and purified using a modification of a previously published workflow^85^. In brief, a gBlock was designed and purchased containing residues 1,915–2,225 of the human CAD protein (cDNA from transcript ENST00000264705.9) separated from an N-terminal 6×His-tagged MBP by a tobacco etch virus (TEV) protease cleavage site, with an SSG linker separating the 6×His tag from MBP, two SSG linkers immediately 5’ of the TEV site and a GS linker immediately 3’ of the TEV site. Gibson Assembly primers were designed using NEBuilder (New England Biolabs) (Supplementary Table 2) and used to amplify and incorporate Gibson overhangs onto the backbone and insert using Q5 hi-fidelity PCR Mastermix (NEB, M0492). This construct was assembled into a pET-28a(+) backbone using GeneArt Hifi Gibson Assembly Mastermix (Invitrogen, A46627), transformed into TOP10 competent cells (Invitrogen, C404003), purified using miniprep/midiprep kits (QIAGEN) and verified by long-read sequencing (Plasmidsaurus).

For protein expression, the MBP-ATCase construct was transformed into BL21 competent cells (Fisher, EC0114) and grown in autoinduction medium^86^ at 37 °C for 6 h, then 18 °C for 24 h to induce protein expression. Bacterial pellets were sonicated at 70% amplitude, 2 s on–5 s off for 5 min on ice in lysis buffer (20 mM Tris-HCl, pH 7.2, 500 mM NaCl, 10 mM imidazole, 5% glycerol, 0.5 mg ml^−1^ lysozyme (Thermo, 89833), 0.5% octyl-B-D-thioglucopyranoside (CHEM-IMPEX, 21810), 2 mM PMSF (Roche) and 2 mM β-mercaptoethanol (Sigma, M3148), and the pH was adjusted to 8.0 with NaOH), after which, Benzonase (~25 U ml^−1^), MgCl_2_ (1 mM) and CaCl_2_ (1 mM) were added and the lysate was incubated on ice for 15 min. The lysate was cleared by ultracentrifugation and batch-bound to HisPur Cobalt Resin (Thermo, 89964) equilibrated with binding buffer (20 mM Tris-HCl pH 7.2, 500 mM NaCl, 10 mM imidazole and 5% glycerol, pH adjusted to 8.0) for 30 min in the cold room with rocking. After binding, resin was moved into a gravity column, washed 3×2 CV with wash buffer (20 mM Tris-HCl, pH 7.2, 500 mM NaCl, 30 mM imidazole, 5% glycerol and 2 mM β-mercaptoethanol, pH adjusted to 8.0) and eluted with 5×1 CV elution buffer (wash buffer with 300 mM imidazole). MBP-ATCase presence and affinity purification was confirmed by SDS–PAGE of appropriate fractions on Bolt 4–12% Bis-Tris Plus polyacrylamide gels (Invitrogen) and subsequent Coomassie staining. Following affinity purification, elute fractions containing MBP-ATCase were pooled, concentrated to <2 ml using a 10,000 kDa MW-cutoff spin column (Amicon, UFC901024) and loaded onto a Superdex 200 16/600 column (Cytiva) equilibrated in gel filtration buffer (20 mM Tris-HCl, pH 7.2, 100 mM NaCl, 2% glycerol and 0.2 mM TCEP (Sigma, 646547)) for size-exclusion chromatography (SEC) on an ÅKTA machine. Following SEC, another diagnostic protein gel was run, and the fractions indicated in Extended Data Fig. 6c were pooled and stored at 4 °C for use in downstream analyses. Final protein concentrations were estimated using spectrophotometry (Nanodrop).

While initial efforts attempted to cleave off the 6×His-MBP tag using overnight dialysis in buffer containing TEV protease, it was found that isolated ATCase was unstable in solution. This fact, and the fact that ATCase has very few aromatic residues facilitating accurate detection and quantification by UV absorbance or BCA assays^85^, led to the decision to use the MBP-ATCase fusion protein in subsequent studies.

### ATCase activity assays

A colorimetric ATCase activity assays was established with minor modifications from previous studies^40,41,87^. Antipyrine/H_2_SO_4_ (5 g l^−1^ antipyrine (Sigma, A5882) in 50% *v*/*v* sulfuric acid (Sigma, 258105)) and oxime (8 g l^−1^ butanedione monoxime (Sigma, 31550) in 5% *v*/*v* glacial acetic acid (Millipore, K52336663013)) reagents were prepared and stored according to a previous study^87^. In brief, purified MBP-ATCase was diluted in cold activity assay buffer (50 mM Tris-acetate, pH 8.3, 0.01 mg ml^−1^ BSA fraction V fatty acid-free (Roche, 03117057001)) at ~0.12 μM. Enzyme was portioned out into a 96-well plate at 50 μl per well, mixed with appropriate substrates (aspartate, succinate and/or fumarate) diluted to 3× final concentration in activity assay buffer and pre-incubated on the benchtop for 5 min. Reactions were started by rapidly adding 50 μl per well CP diluted to 3× final concentration in activity assay buffer and allowed to proceed for 5 min in a benchtop incubator set to 37 °C. After incubation, reactions were quenched by addition of 150 μl per well colour solution (2:1 antipyrine/ H_2_SO_4_: oxime reagents, prepared immediately before use), sealing the plate with plate film (Thermo), pulse vortexing and heating on top of a 95 °C heat block for ~30 min to 1 h in room light. Plates were cooled to room temperature, and absorbance at 460 nm was subsequently quantified using a plate reader (Tecan). Blank controls were used to subtract background absorbance, and carbamoyl-aspartate calibration curves were run in each activity assay to verify linearity of the measured absorbances.

### Mass photometry

MP was performed on a Two^MP^ instrument (Refeyn) similarly to what was previously described^88^ using AcquireMP v.2024 and DiscoverMP v.2024 software for acquisition and analysis, respectively. MBP-ATCase (~40 nM) and appropriate substrates were prepared in MP buffer (50 mM Tris-acetate, pH 8.3) on ice and incubated for 2–4 min before imaging on uncoated glass slides (Refeyn). Droplet dilution autofocus was used to find focus in 20-μl droplet sizes. Mass calibrations were performed before each instrument run using MassFerence P1 calibrant (Refeyn, MP-CON-41033) according to manufacturer specifications. Multiple 1-min videos were collected per sample, and verified to agree qualitatively, and Gaussian fits were computed using DiscoverMP.

### Puromycin incorporation assay

143B cells were seeded in a six-well plate at 200,000 cells per well. The following day, cells were washed twice with Dulbecco’s PBS (DPBS) and switched into appropriate treatment medium (2 ml per well, DMEM + 10% dialysed FBS) for 24 h. Puromycin incorporation was conducted by spiking in puromycin (Sigma, P9620) at 10 μg ml^−1^ for exactly 30 min before cells were washed once again and protein lysates were collected in RIPA buffer (Sigma, R0278) supplemented with protease inhibitors (Fisher, A32953) and phosphatase inhibitors (Fisher, 78442). Then, 100 μg ml^−1^ cycloheximide (Cayman, 14126) was added to the appropriate samples 30 min before puromycin addition. Protein concentrations were determined and western blotting was performed as above. Membranes were incubated at 4 °C overnight with the following antibodies: anti-puromycin (Kerafast, Kf-Ab02366–1.1, 1:1,000 dilution) and anti-GAPDH (Cell Signalling, 5174S, 1:5,000 dilution) and imaged as above. Densitometry on entire puromycin lanes was performed using ImageJ2 v.2.9.0.

### Cell cycle analysis

For cell cycle analysis, cells were plated in six-well plates at 100,000 cells per well and incubated overnight. The following day, cells were washed three times with DPBS and switched into appropriate treatment medium (3 ml well, DMEM + 10% dialysed FBS) for the indicated times. To fix cells, replicate wells were trypsinized, pelleted and washed twice with DPBS. Cells were resuspended in 300 μl ice-cold PBS, and 700 μl ice-cold 100% ethanol was added dropwise to each sample while vortexing to fix. Fixed cells were stored at −20 °C until being processed for flow cytometry (no longer than 4 days). After all time points were collected, fixed cells were pelleted and washed with DPBS twice, then resuspended in 250 μl of 50 μg ml^−1^ propidium iodide (Biotium, 40017) with 100 μg ml ^−1^ RNase A (QIAGEN) staining solution for 1 h at room temperature or overnight at 4 °C, protected from light. Samples were then passed through a 0.35-μm filter into flow cytometry tubes (Falcon) before being analysed on a BD FACSymphony A52 Cell Analyzer running FACSDiva software. 10,000 events were recorded per sample. Data were analysed using the ‘Cell Cycle’ analysis module of FlowJo v.10.10.1.

### Animal studies

The *Rb1^lox/lox^; Trp53^lox/lox^; Ascl1-Cre-ERT2* model of neuroendocrine pituitary tumorigenesis, as described^89^ were bred to a *Sdhb^lox/lox^* allele from J. Favier^34^ to generate compound mutant mice of mixed C57BL6/129 background, used for this study. Tamoxifen induction of Cre-ERT2 under the control of *Ascl1* promoter was performed at 6 weeks of age by intraperitoneal injection of mice with tamoxifen (150 mg kg^−1^ day^−1^), prepared in sterile corn oil, over five consecutive days. Mice were killed when they exhibited poor body condition and necropsies were performed, with the skull either fixed in Bouin’s fixative or bisected, with part of the pituitary tumour excised and snap frozen on dry ice and the remainder fixed in the skull with Bouin’s for 1 week before processing and paraffin embedding. Tissue sections (4-μm thick) were stained with haematoxylin and eosin. Both male and female animals were used, but sex was not specifically accounted for in our analyses. All mouse experiments were reviewed and approved by the Fred Hutchinson Cancer Center Institutional Animal Care and Use Committee under protocol number 50783. Mice were housed on a 12-h light–dark cycle with controlled temperature (65–75 °F) and humidity (40–60%). Animals were fed with Inotiv’s Teklad 2918 Irradiated Global 18% protein rodent diet. All animals were housed in individually ventilated and HEPA-filtered microisolator cage Seal Safe GM500 cages from Tecniplast. Cages were changed every 2 weeks and feed and water were supplied ad libitum.

### Mouse pituitary tumour metabolomics and western blotting

For LC–MS analyses, frozen tumour chunks from RP/RP-SDHB mice were pulverized using liquid nitrogen-cooled instruments and portioned into pre-weighed tubes. HPLC-grade 80% methanol was added at 1 ml per 20 mg tissue and samples were vortexed for 30 min at room temperature to extract polar metabolites. Samples were spun at 17,000*g* for 15 min in a refrigerated centrifuge and 100 μl of supernatant was dried down in a refrigerated vacuum centrifuge overnight (Centrivap). Each sample was reconstituted in 500 μl of a 1:1 mix of ^13^C-labelled yeast and spirulina extracts (see above) and LC–MS was performed using a Q Exactive HF-X Hybrid Quadrupole-Orbitrap Mass Spectrometer as above. Wherever possible, ^13^C-labelled internal standards were used to calculate response ratios and correct for matrix effects. Due to systemic differences in global metabolite ion counts across samples, a ‘correction factor’ was created, consisting of the mean of the response ratios for five amino acids (leucine, lysine, threonine, tyrosine and phenylalanine), and ion counts for metabolites investigated in Fig. 6 were subsequently normalized using this factor. While this approach has been used in LC–MS studies previously^4^, we note that the carb-asp:aspartate ratio is insensitive to these systemic signal differences and is identically different between RP and RP-SDHB extracts whether or not this correction factor is used.

For western blotting, cell material pellets leftover after the spin step above were briefly dried in a refrigerated vacuum centrifuge to remove residual extraction solvent, and 200 μl RIPA buffer with protease/ phosphatase inhibitors and 5 mM EDTA was added per sample. Samples were vortexed for 5 min at room temperature and incubated on ice for 15 min, then this vortexing/incubation procedure was repeated once more. Protein concentrations were determined and western blotting was performed as above. Densitometry was performed using ImageJ2 v.2.9.0.

### Statistics and reproducibility

All graphs and statistical analyses were made in GraphPad Prism v.10.4.1. Replicates, defined as parallel biological samples independently treated, collected and analysed during the same experiment, are shown. Experiments were verified with two or more independent repetitions showing qualitatively similar results. Details pertaining to all statistical tests can be found in the figure legends. No statistical test was used to predetermine sample sizes, but our sample sizes are similar to those reported in previous publications^2,5,22^. The investigators were not blinded to allocation during experiments and outcome assessment. The experiments were not randomized. Data were only excluded during occasional Incucyte scans, where the microscope/ camera malfunctioned and no cells were found in the images. Data distribution was assumed to be normal but this was not formally tested.

### Reporting summary

Further information on research design is available in the Nature Portfolio Reporting Summary linked to this article.

## Extended Data

## Supplementary Material

Supplementary Information

Supplementary Video 1

Supplementary Video 2

Supplementary Video 3

Supplementary Video 4

Supplementary Video 5

The online version contains supplementary material available at https://doi.org/10.1038/s42255-026-01524-w.

## Data Availability

All data supporting the findings of this study are available within the paper and its Supplementary Information. Source data are provided with this paper.

## Code availability

For Extended Data Fig. 4, relevant data, code and analysis can be found at https://github.com/krdav/lab-work/tree/main/nucleotide_salvage_tracing.
