# Supplementary material for: Succinate dehydrogenase loss suppresses pyrimidine biosynthesis via succinate-mediated inhibition of aspartate transcarbamylase
Source: Nat Metab. 2026 May 4;8(6):1390–409. doi: 10.1038/s42255-026-01524-w (PMC13303085; doi:10.1038/s42255-026-01524-w)
Supplement: Supplementary file 1 — Supplementary Table 1: FH-KO guide RNAs. Supplementary Table 2: ATCase cloning primers. [file 42255_2026_1524_MOESM1_ESM.pdf]

nature metabolism

Supplementary information https://doi.org/10.1038/s42255-026-01524-w

Succinate dehydrogenase loss suppresses pyrimidine biosynthesis via succinate-mediated inhibition of aspartate transcarbamylase

In the format provided by the authors and unedited

Supplementary Table 1: FH KO guide RNAs

Guide	Sequence 5' → 3'
FH sgRNA1	AGGCAAGCCAAAAUUCCUUC
FH sgRNA2	GGUACAUAUUCUAUCCGGA
FH sgRNA3	CAAAGGUAUCAUAUUCUAUC

Supplementary Table 2: ATCase Cloning Primers

Primer	Sequence
backbone F	ctcgagcaccaccaccac
backbone R	gctgctgcccatggtatatc
insert F	accatgggcagcagccatcaccatcatcaccacag
insert R	gtggtggtgctcgagttagaaacggcccagcac
