# Supplementary material for: Succinate dehydrogenase loss suppresses pyrimidine biosynthesis via succinate-mediated inhibition of aspartate transcarbamylase
Source: Nat Metab. 2026 May 4;8(6):1390–409. doi: 10.1038/s42255-026-01524-w (PMC13303085; doi:10.1038/s42255-026-01524-w)
Supplement: Supplementary file 2 — Reporting Summary [file 42255_2026_1524_MOESM2_ESM.pdf]

nature portfolio

Corresponding author(s): Lucas B. Sullivan

Last updated by author(s): Mar 21, 2026

Reporting Summary

Nature Portfolio wishes to improve the reproducibility of the work that we publish. This form provides structure for consistency and transparency in reporting. For further information on Nature Portfolio policies, see our Editorial Policies and the Editorial Policy Checklist.

Statistics

For all statistical analyses, confirm that the following items are present in the figure legend, table legend, main text, or Methods section.

n/a	Confirmed	
☐	☒	The exact sample size (*n*) for each experimental group/condition, given as a discrete number and unit of measurement
☐	☒	A statement on whether measurements were taken from distinct samples or whether the same sample was measured repeatedly
☐	☒	The statistical test(s) used AND whether they are one- or two-sided<br>*Only common tests should be described solely by name; describe more complex techniques in the Methods section.*
☒	☐	A description of all covariates tested
☐	☒	A description of any assumptions or corrections, such as tests of normality and adjustment for multiple comparisons
☐	☒	A full description of the statistical parameters including central tendency (e.g. means) or other basic estimates (e.g. regression coefficient) AND variation (e.g. standard deviation) or associated estimates of uncertainty (e.g. confidence intervals)
☐	☒	For null hypothesis testing, the test statistic (e.g. *F*, *t*, *r*) with confidence intervals, effect sizes, degrees of freedom and *P* value noted<br>*Give P values as exact values whenever suitable.*
☒	☐	For Bayesian analysis, information on the choice of priors and Markov chain Monte Carlo settings
☒	☐	For hierarchical and complex designs, identification of the appropriate level for tests and full reporting of outcomes
☒	☐	Estimates of effect sizes (e.g. Cohen's *d*, Pearson's *r*), indicating how they were calculated

Our web collection on statistics for biologists contains articles on many of the points above.

Software and code

Policy information about availability of computer code

Data collection	Data collection for Incucyte experiments was performed using Sartorius Incucyte software (v2023A). Cell counts and cell size measurements were obtained by Beckman Coulter Multisizer 4 Particle Counter (Multi4) (v4.01). Western Blots were imaged and processed using LiCOR Image Studio Lite (v5.2.5).
Data analysis	TraceFinder (v4.1), FreeStyle (v1.3.115.19), GraphPad Prism (v10.4.1), R Studio (2023.09.1+494). All software and code used to analyze data in Extended Data Figures 4 can be found at https://github.com/krdav/lab-work/tree/main/nucleotide_salvage_tracing.

For manuscripts utilizing custom algorithms or software that are central to the research but not yet described in published literature, software must be made available to editors and reviewers. We strongly encourage code deposition in a community repository (e.g. GitHub). See the Nature Portfolio guidelines for submitting code & software for further information.

Data

Policy information about availability of data

All manuscripts must include a data availability statement. This statement should provide the following information, where applicable:

- Accession codes, unique identifiers, or web links for publicly available datasets
- A description of any restrictions on data availability
- For clinical datasets or third party data, please ensure that the statement adheres to our policy

We provide all source data within the manuscript and supplementary files. Raw ion counts for LC-MS data are provided in a supplemntary file. Raw GFP/RFP values

(per well) and raw nuclear RFP counts (per well) are also provided. tRNA aminoacylation charge as shown in Extended Data Figure 3 is provided in the supplement for all 20 amino acids. Uncropped western blot and gel images are provided in the source data.

Research involving human participants, their data, or biological material

Policy information about studies with human participants or human data. See also policy information about sex, gender (identity/presentation), and sexual orientation and race, ethnicity and racism.

Reporting on sex and gender	*Use the terms sex (biological attribute) and gender (shaped by social and cultural circumstances) carefully in order to avoid confusing both terms. Indicate if findings apply to only one sex or gender; describe whether sex and gender were considered in study design; whether sex and/or gender was determined based on self-reporting or assigned and methods used. Provide in the source data disaggregated sex and gender data, where this information has been collected, and if consent has been obtained for sharing of individual-level data; provide overall numbers in this Reporting Summary. Please state if this information has not been collected. Report sex- and gender-based analyses where performed, justify reasons for lack of sex- and gender-based analysis.*
Reporting on race, ethnicity, or other socially relevant groupings	*Please specify the socially constructed or socially relevant categorization variable(s) used in your manuscript and explain why they were used. Please note that such variables should not be used as proxies for other socially constructed/relevant variables (for example, race or ethnicity should not be used as a proxy for socioeconomic status). Provide clear definitions of the relevant terms used, how they were provided (by the participants/respondents, the researchers, or third parties), and the method(s) used to classify people into the different categories (e.g. self-report, census or administrative data, social media data, etc.) Please provide details about how you controlled for confounding variables in your analyses.*
Population characteristics	*Describe the covariate-relevant population characteristics of the human research participants (e.g. age, genotypic information, past and current diagnosis and treatment categories). If you filled out the behavioural & social sciences study design questions and have nothing to add here, write "See above."*
Recruitment	*Describe how participants were recruited. Outline any potential self-selection bias or other biases that may be present and how these are likely to impact results.*
Ethics oversight	*Identify the organization(s) that approved the study protocol.*

Note that full information on the approval of the study protocol must also be provided in the manuscript.

Field-specific reporting

Please select the one below that is the best fit for your research. If you are not sure, read the appropriate sections before making your selection.

☒ Life sciences ☐ Behavioural & social sciences ☐ Ecological, evolutionary & environmental sciences

For a reference copy of the document with all sections, see nature.com/documents/nr-reporting-summary-flat.pdf

Life sciences study design

All studies must disclose on these points even when the disclosure is negative.

Sample size	No statistical method was used to predetermine sample sizes. Similar to our previously published work, our sample sizes for every experiment were n=2-4 as technical replicates.
Data exclusions	No data were excluded from this study.
Replication	To verify the results of our findings, each experiment was conducted using at least two biological replications. All published results were reproducible.
Randomization	No requirements for randomization in this study.
Blinding	The investigators were not blinded in this study, as it was not possible for the in vitro studies presented in this manuscript.

Reporting for specific materials, systems and methods

We require information from authors about some types of materials, experimental systems and methods used in many studies. Here, indicate whether each material, system or method listed is relevant to your study. If you are not sure if a list item applies to your research, read the appropriate section before selecting a response.

Materials & experimental systems

n/a	Involved in the study	
☐	☒	Antibodies
☐	☒	Eukaryotic cell lines
☒	☐	Palaeontology and archaeology
☐	☒	Animals and other organisms
☒	☐	Clinical data
☒	☐	Dual use research of concern
☒	☐	Plants

Methods

n/a	Involved in the study	
☒	☐	ChIP-seq
☐	☒	Flow cytometry
☒	☐	MRI-based neuroimaging

Antibodies

Antibodies used

All antibodies, catalog numbers, and dilutions are provided here and in the methods section. anti-GOT2 (Proteintech, 14800-1-AP, 1:1000), anti-GOT1 (Cell Signaling, 34423S, 1:1,000), anti-GFP (Sigma, 1:1000), anti-FH (Origene, TA500675S, 1:1000), anti-SDHB (Atlas, HPA002868, 1:1,000), anti-pChk1 (Cell Signaling, 2348S, 1:1000), anti-Chk1 (Cell Signaling, 2G1D5, 1:1000), anti-pChk2 (Cell Signaling, 2197S, 1:1000), anti-GAPDH (Cell Signaling, 5174S, 1:5000), and anti-Vinculin (Sigma, SAB4200729, 1:10,000), anti-tubulin (Sigma, T6199). The next morning, membranes were washed three times with TBS-T, and the following secondary antibodies were added: 800CW Goat anti-Mouse IgG (LiCOR, 926-32210; 1:15,000), 680RD Goat anti-Rabbit IgG (LiCOR, 926-68071; 1:15,000).

Validation

The following antibodies are Rabbit: GOT2, GOT1, SDHB, p-Chk1, pChk2, GAPDH. The following are Mouse: GFP, FH, Vinculin, Tubulin. Antibody validation is provided on the manufacturer website. We also validated each antibody in our respective cell lines with a positive control for each western blot.

Eukaryotic cell lines

Policy information about cell lines and Sex and Gender in Research

Cell line source(s)

Human cancer cell lines (143B, H1299, HCT116, HT-1080) and LentiX-293T cells were obtained from the American Type Culture Collection (ATCC).

Authentication

All cell lines were authenticated by STR analysis at Fred Hutch.

Mycoplasma contamination

All cells lines routinely tested negative for mycoplasma.

Commonly misidentified lines (See ICLAC register)

No commonly misidentified cell lines were used.

Animals and other research organisms

Policy information about studies involving animals; ARRIVE guidelines recommended for reporting animal research, and Sex and Gender in Research

Laboratory animals

The Rb1lox/lox; Trp53lox/lox; Ascl1-Cre-ERT2 model of neuroendocrine pituitary tumorigenesis, as described were bred to a Sdhblox/lox allele to generate compound mutant mice of mixed C57BL6/129. Tamoxifen was administered to both male and female mice at 6 weeks of age to initate tumor formation and mice were euthanized when they became to burdered by disease (around 150-230 days later). See Extended Data Fig 9g for specific ages.

Wild animals

N/A

Reporting on sex

Sex was not a consideration in the original study design.

Field-collected samples

N/A

Ethics oversight

All mouse experiments were reviewed and approved by the Fred Hutchinson Cancer Center IACUC under Protocol Number 50783.

Note that full information on the approval of the study protocol must also be provided in the manuscript.

Plants

Seed stocks	*Report on the source of all seed stocks or other plant material used. If applicable, state the seed stock centre and catalogue number. If plant specimens were collected from the field, describe the collection location, date and sampling procedures.*
Novel plant genotypes	*Describe the methods by which all novel plant genotypes were produced. This includes those generated by transgenic approaches, gene editing, chemical/radiation-based mutagenesis and hybridization. For transgenic lines, describe the transformation method, the number of independent lines analyzed and the generation upon which experiments were performed. For gene-edited lines, describe the editor used, the endogenous sequence targeted for editing, the targeting guide RNA sequence (if applicable) and how the editor was applied.*
Authentication	*Describe any authentication procedures for each seed stock used or novel genotype generated. Describe any experiments used to assess the effect of a mutation and, where applicable, how potential secondary effects (e.g. second site T-DNA insertions, mosiacism, off-target gene editing) were examined.*

Flow Cytometry

Plots

Confirm that:

☐ The axis labels state the marker and fluorochrome used (e.g. CD4-FITC).

☐ The axis scales are clearly visible. Include numbers along axes only for bottom left plot of group (a 'group' is an analysis of identical markers).

☐ All plots are contour plots with outliers or pseudocolor plots.

☐ A numerical value for number of cells or percentage (with statistics) is provided.

Methodology

Sample preparation	For cell cycle analysis, cells were plated in 6-well plates at 100K cells/well and incubated overnight. The following day, cells were washed three times with Dulbecco's PBS (DPBS) and switched into appropriate treatment medias (3 mL/well, DMEM + 10% dialyzed FBS) for the indicated times. To fix cells, replicate wells were trypsinized, pelleted and washed twice with DPBS. Cells were resuspended in 300 µL ice-cold PBS, and 700 µL ice-cold 100% ethanol was added dropwise to each sample while vortexing to fix. Fixed cells were stored at -20°C until being processed for flow cytometry (no longer than 4 days). After all timepoints were collected, fixed cells were pelleted and washed with DPBS twice, then resuspended in 250 µL of 50 µg/mL propidium iodide (Biotium, 40017) with 100 µg/mL RNase A (Qiagen) staining solution for 1 hour at room temperature or overnight at 4°C, protected from light. Samples were then passed through a 0.35-µm filter into flow cytometry tubes (Falcon). 10,000 events were recorded per sample.
Instrument	BD FACSymphony A52 Cell Analyzer.
Software	Symphony was run using FACSDiva software. Data were analyzed using the 'Cell Cycle' analysis module of FlowJo 10.10.1.
Cell population abundance	Cell population abundance was optimized for 143B cells and were run according to standard PI staining protocols.
Gating strategy	Single cells were gated using forward and side-scatter, after which PI intensity was measured on the entire gated population. No live/dead stain or other markers were used.

☐ Tick this box to confirm that a figure exemplifying the gating strategy is provided in the Supplementary Information.
