# Supplementary material for: Succinate dehydrogenase loss suppresses pyrimidine biosynthesis via succinate-mediated inhibition of aspartate transcarbamylase
Source: Nat Metab. 2026 May 4;8(6):1390–409. doi: 10.1038/s42255-026-01524-w (PMC13303085; doi:10.1038/s42255-026-01524-w)

Fig. 4d

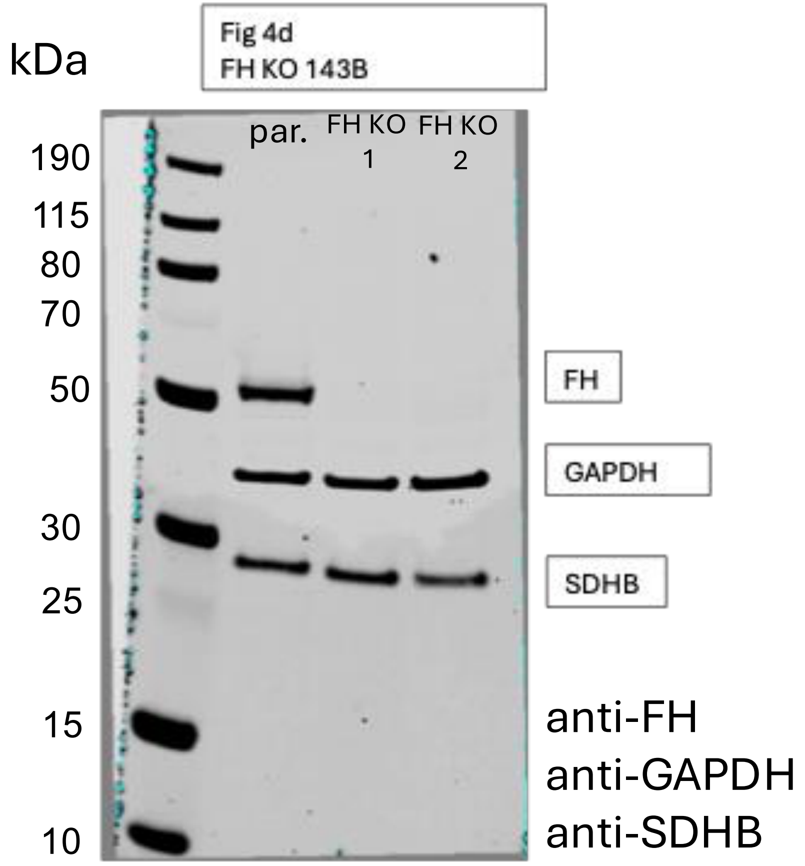

par. = parental

Fig. 5f

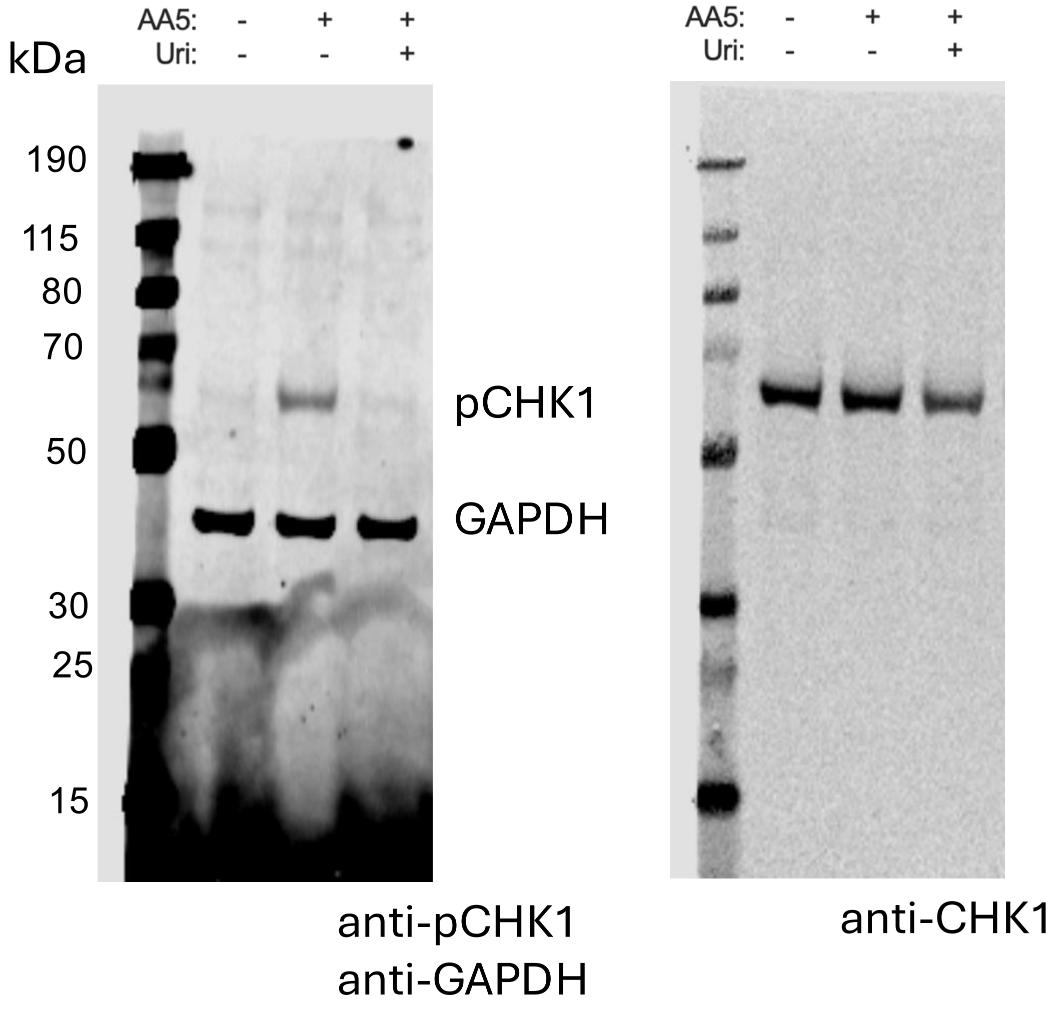
AA5: - + +
Uri: - - +
kDa
190
115
80
70
50
30
25
15
pCHK1
GAPDH
anti-pCHK1
anti-GAPDH
AA5: - + +
Uri: - - +
anti-CHK1

Fig. 6a

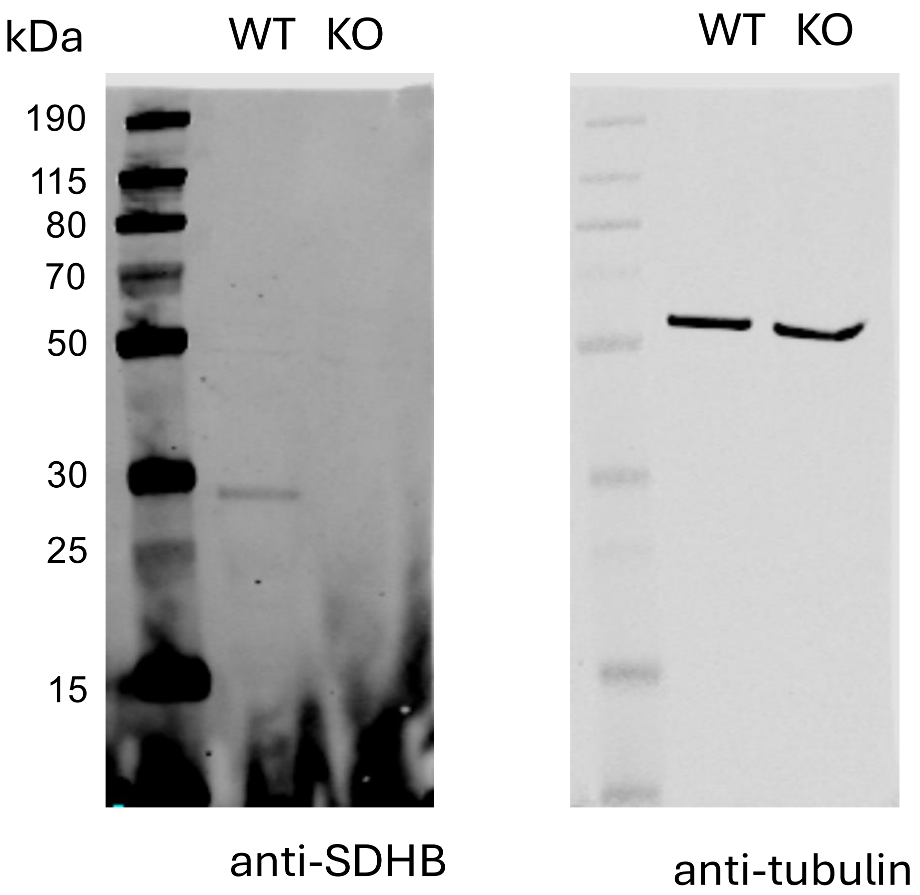

Fig. 6i

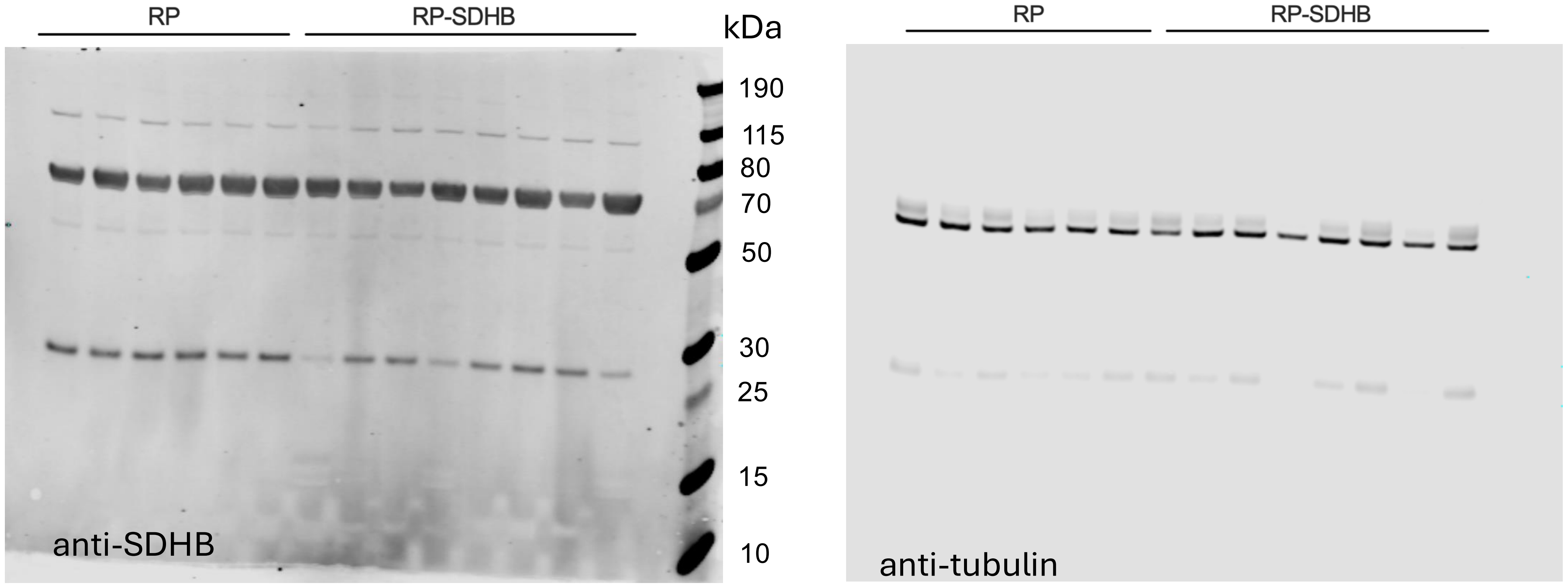
RP
RP-SDHB
kDa
190
115
80
70
50
30
25
15
10
anti-SDHB
RP
RP-SDHB
anti-tubulin

Extended Data Figure 2: 143B/H1299 GOT1/2 DKO

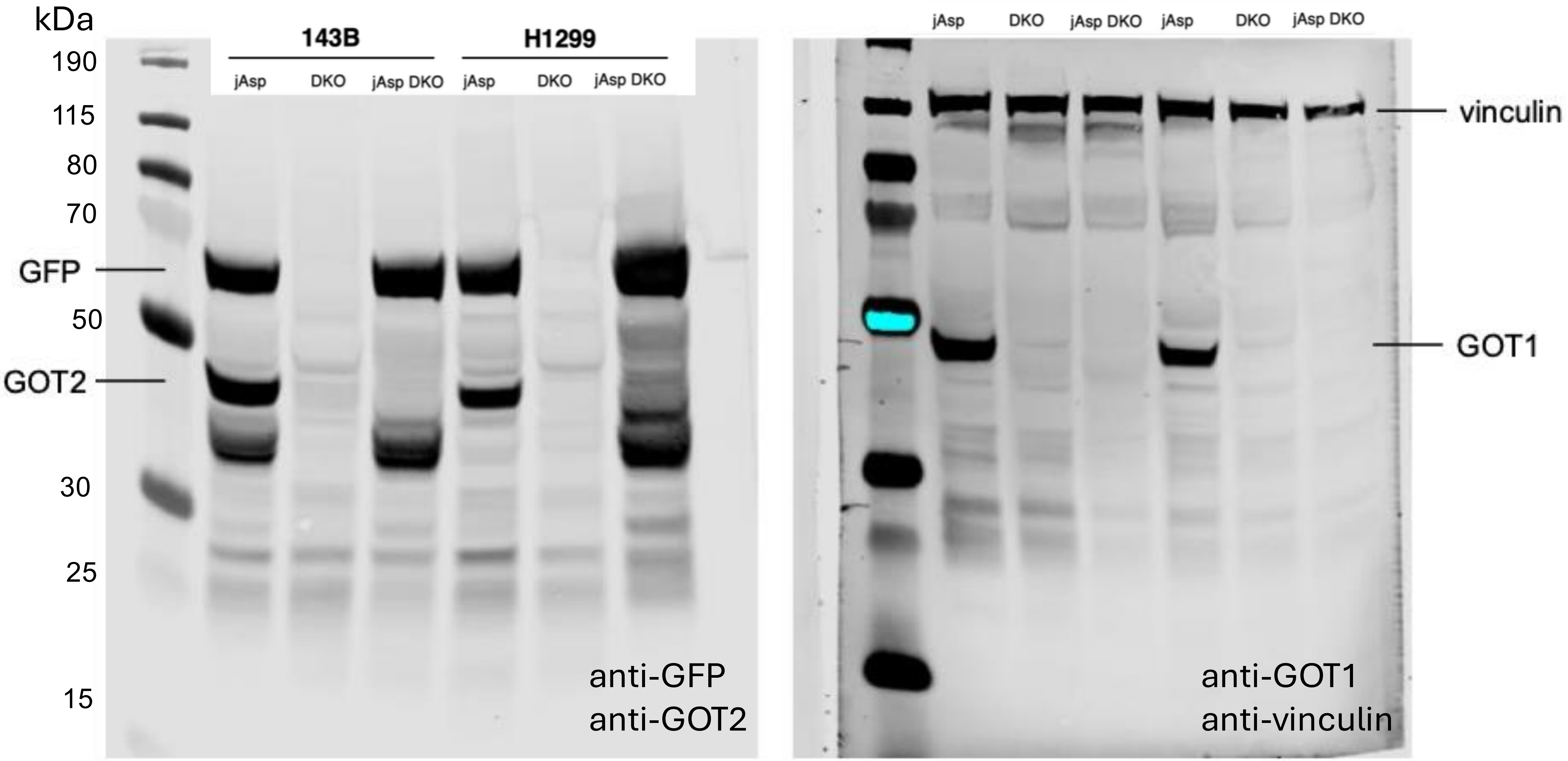

Ext. Data Fig. 5e

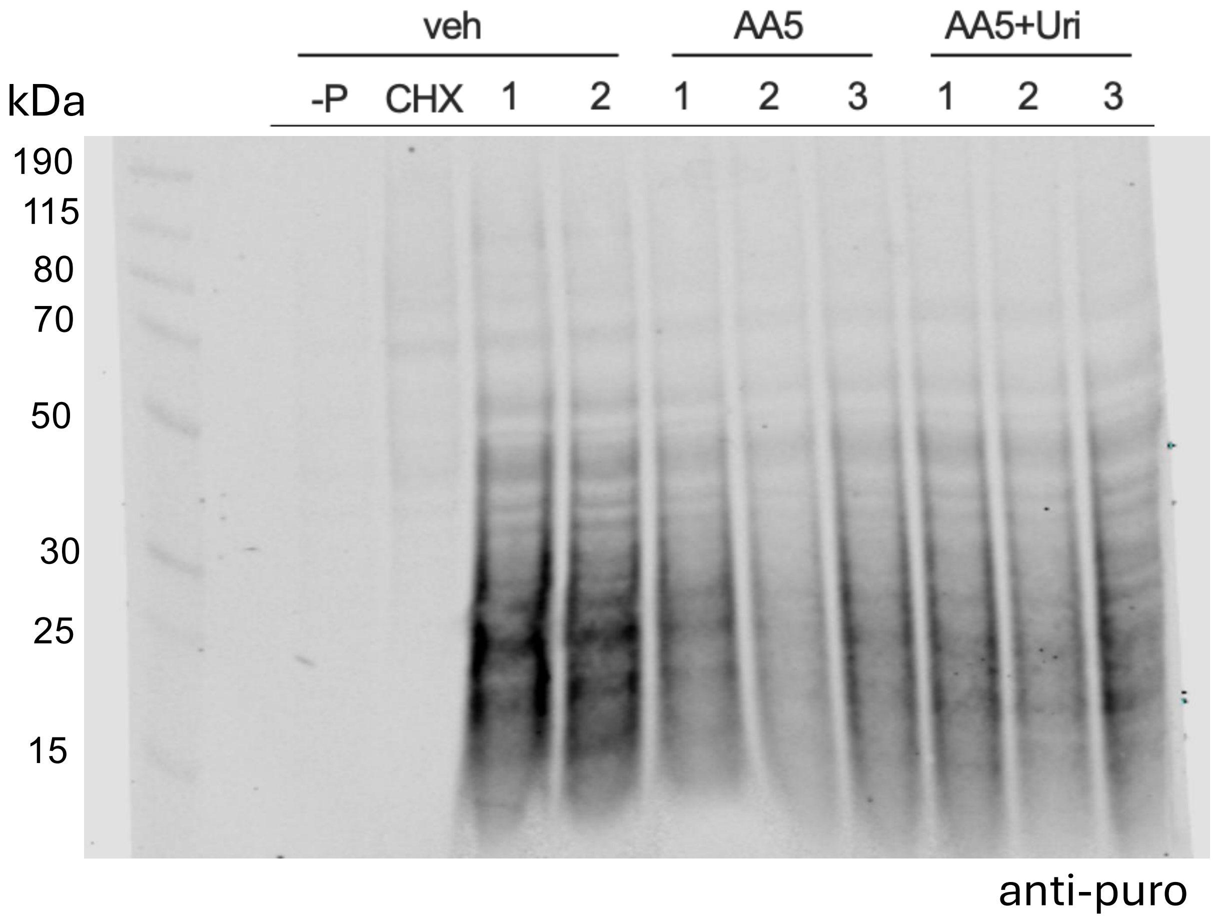

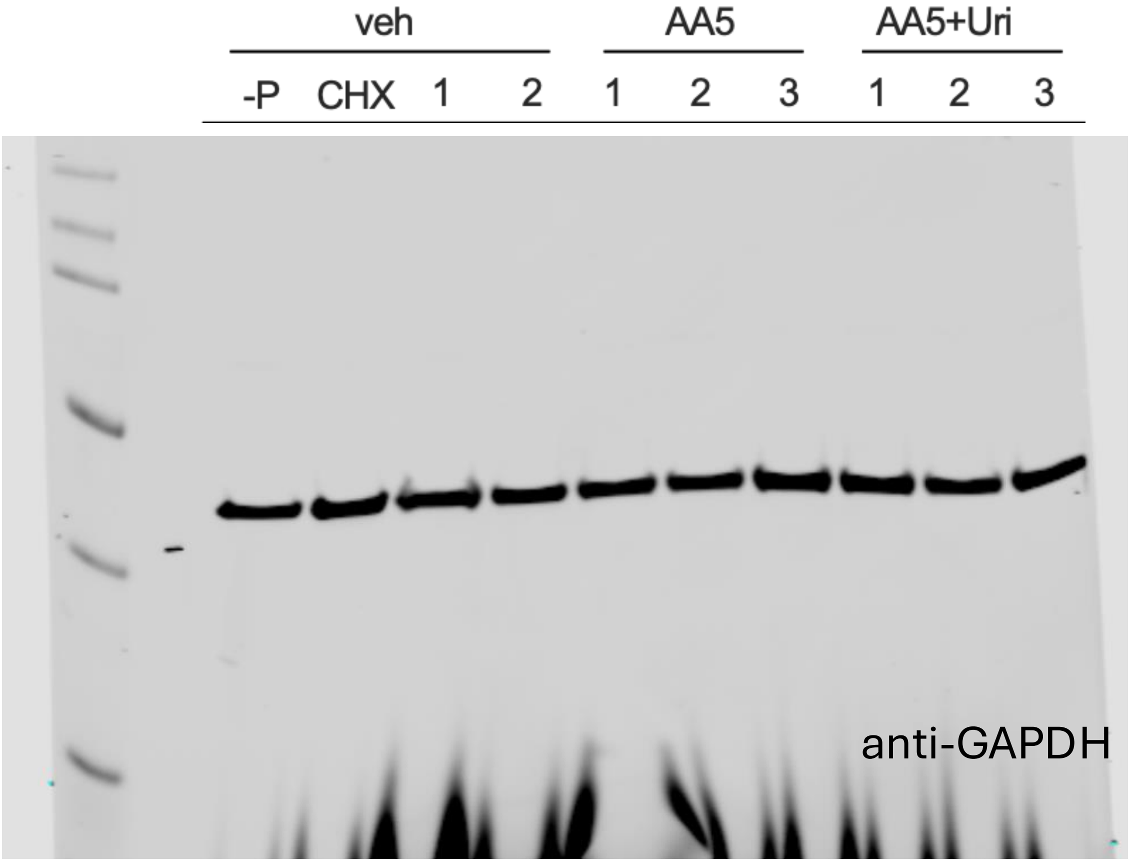

Ext. Data Fig. 6c

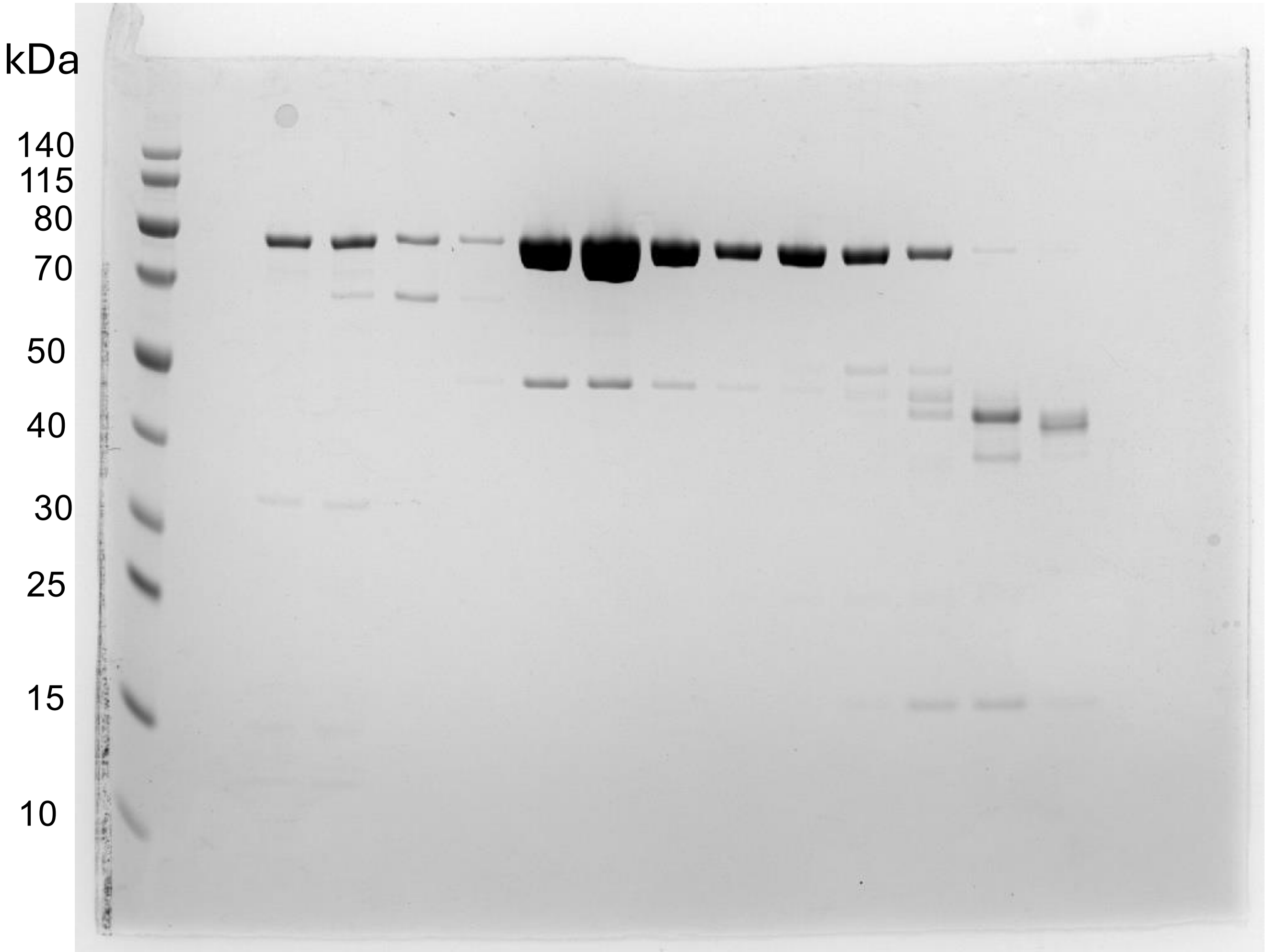

Coomassie-stained protein gel

Ext Data Fig 8c

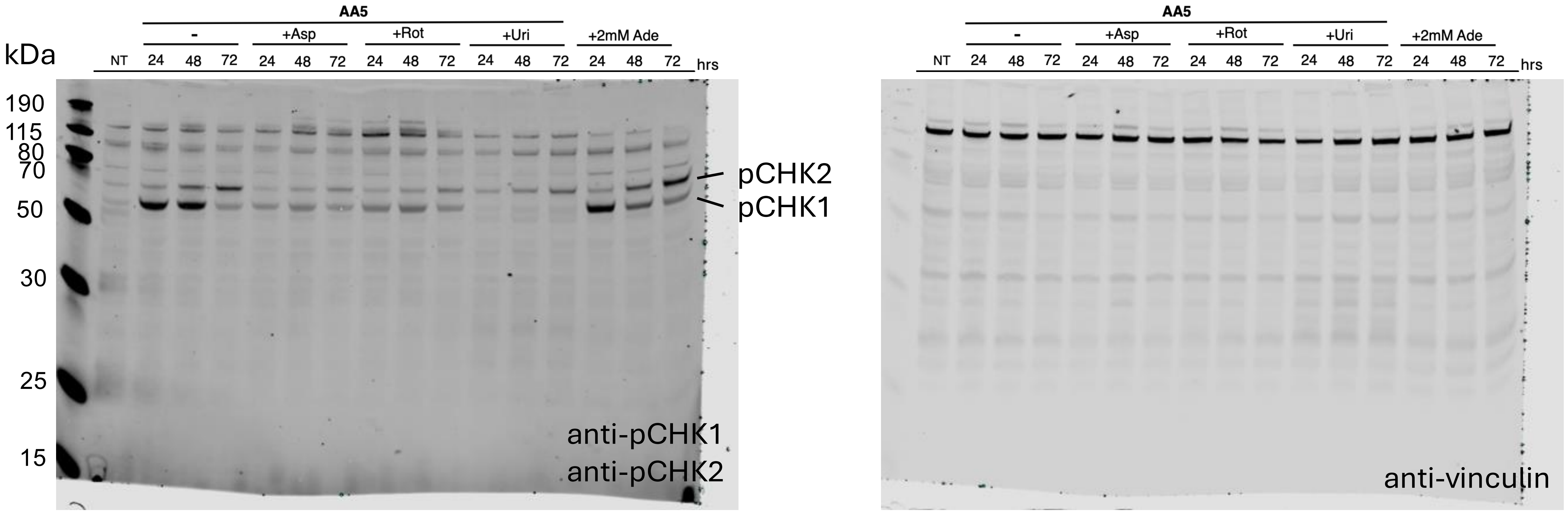

Ext Data Fig 8f

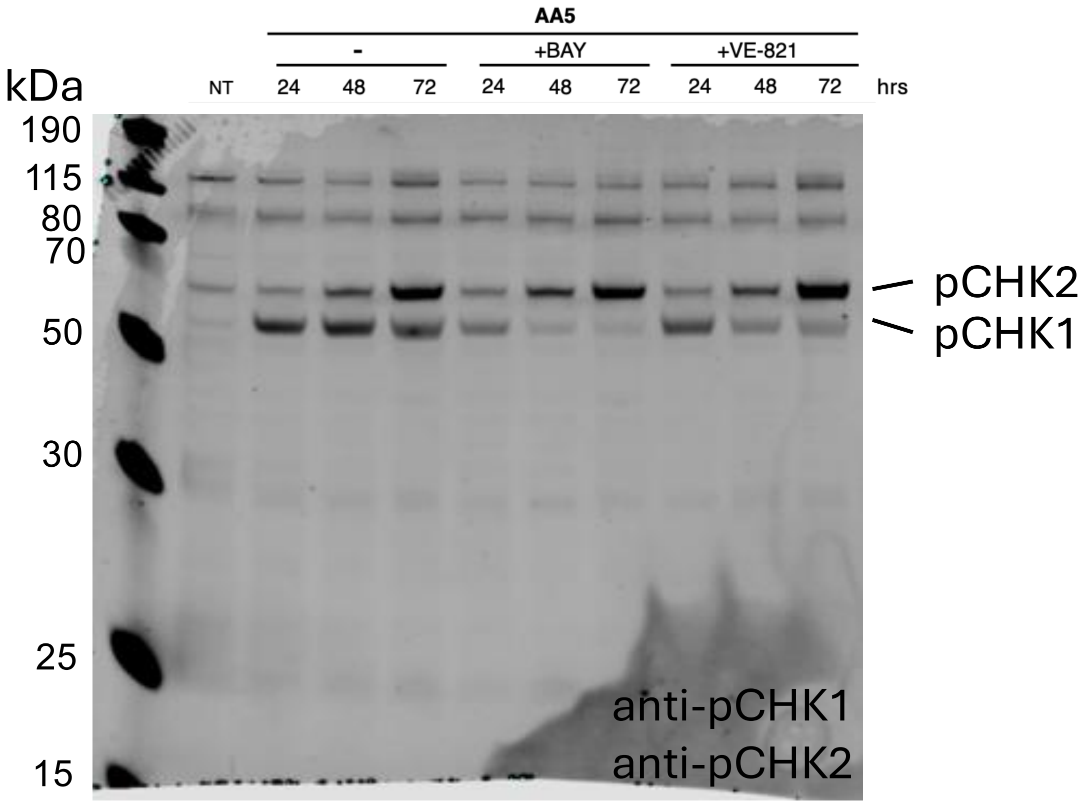

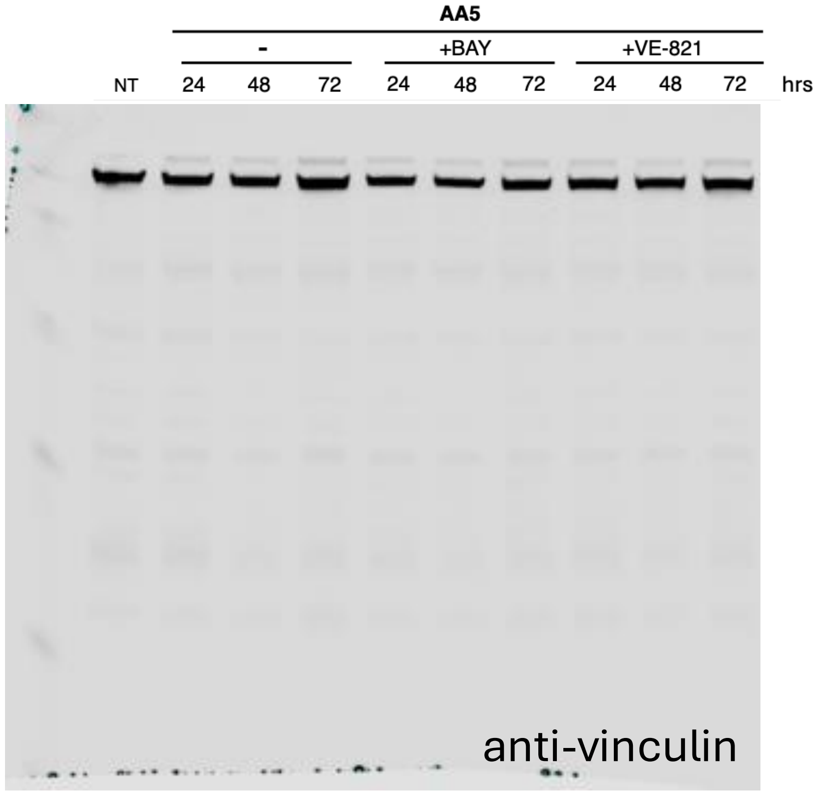

Supplement: Supplementary file 8 — Source data for Figs. 1–6 and Extended Data Figs. 1–9. Images and MolecularDocking_Outputs. [file 42255_2026_1524_MOESM8_ESM.zip › ATCase_SourceData/ATCase_Blots_SourceData_All.pdf]
