# Supplementary figures and images for: Succinate dehydrogenase loss suppresses pyrimidine biosynthesis via succinate-mediated inhibition of aspartate transcarbamylase
Source: Nat Metab. 2026 May 4;8(6):1390–409. doi: 10.1038/s42255-026-01524-w (PMC13303085; doi:10.1038/s42255-026-01524-w)

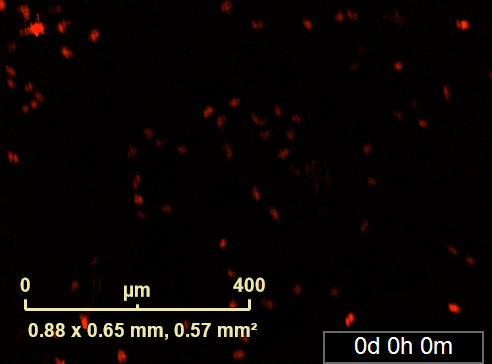

Supplement: Supplementary file 8 — Source data for Figs. 1–6 and Extended Data Figs. 1–9. Images and MolecularDocking_Outputs. [file 42255_2026_1524_MOESM8_ESM.zip › ATCase_SourceData/Source Data_ExtDataFig1_Images/143B_-Pyr_Red_A1_8_00d00h00m.tif]

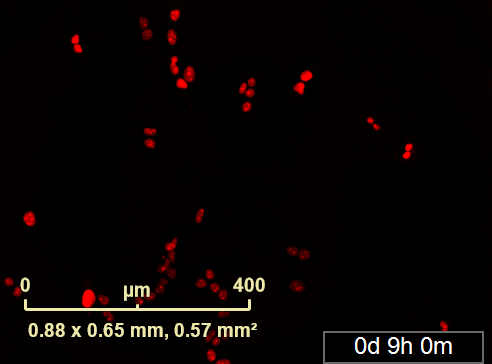

Supplement: Supplementary file 8 — Source data for Figs. 1–6 and Extended Data Figs. 1–9. Images and MolecularDocking_Outputs. [file 42255_2026_1524_MOESM8_ESM.zip › ATCase_SourceData/Source Data_ExtDataFig1_Images/143B_AA5_Red_B2_10_00d09h00m.tif]

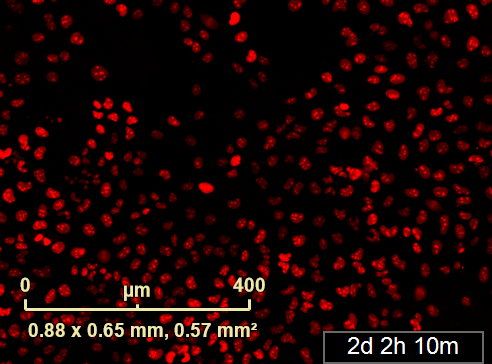

Supplement: Supplementary file 8 — Source data for Figs. 1–6 and Extended Data Figs. 1–9. Images and MolecularDocking_Outputs. [file 42255_2026_1524_MOESM8_ESM.zip › ATCase_SourceData/Source Data_ExtDataFig1_Images/143B_+Pyr_Red_A3_6_02d02h10m.tif]

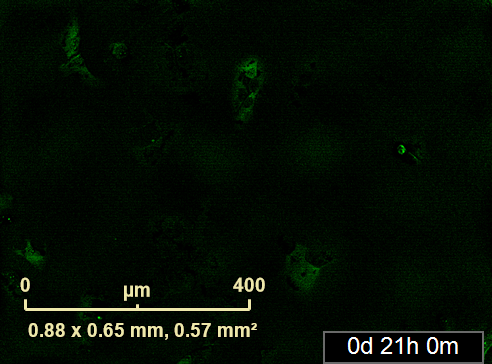

Supplement: Supplementary file 8 — Source data for Figs. 1–6 and Extended Data Figs. 1–9. Images and MolecularDocking_Outputs. [file 42255_2026_1524_MOESM8_ESM.zip › ATCase_SourceData/Source Data_ExtDataFig1_Images/143B_AA5_Green_B2_10_00d21h00m.tif]

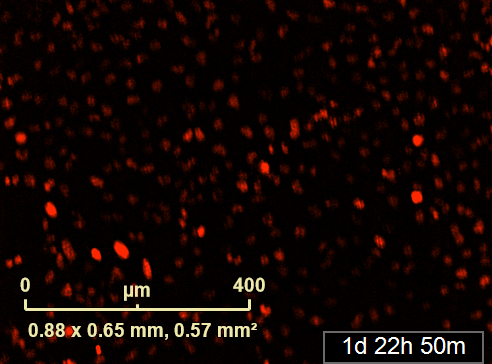

Supplement: Supplementary file 8 — Source data for Figs. 1–6 and Extended Data Figs. 1–9. Images and MolecularDocking_Outputs. [file 42255_2026_1524_MOESM8_ESM.zip › ATCase_SourceData/Source Data_ExtDataFig1_Images/143B_-Pyr_Red_A1_8_01d22h50m.tif]

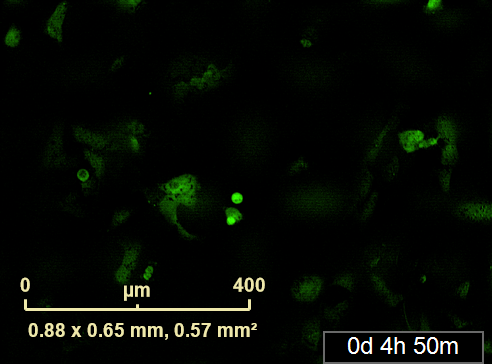

Supplement: Supplementary file 8 — Source data for Figs. 1–6 and Extended Data Figs. 1–9. Images and MolecularDocking_Outputs. [file 42255_2026_1524_MOESM8_ESM.zip › ATCase_SourceData/Source Data_ExtDataFig1_Images/143B_-Pyr_Rot_Green_B5_1_00d04h50m.tif]

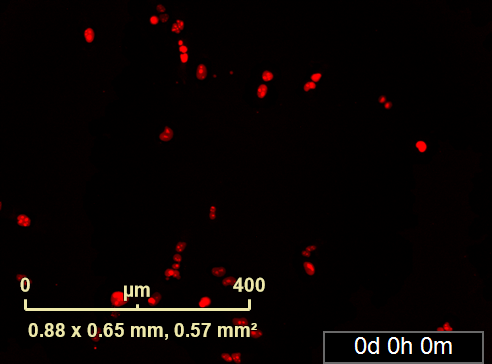

Supplement: Supplementary file 8 — Source data for Figs. 1–6 and Extended Data Figs. 1–9. Images and MolecularDocking_Outputs. [file 42255_2026_1524_MOESM8_ESM.zip › ATCase_SourceData/Source Data_ExtDataFig1_Images/143B_AA5_Red_B2_10_00d00h00m.tif]

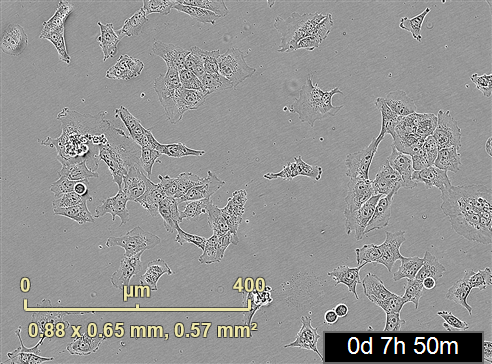

Supplement: Supplementary file 8 — Source data for Figs. 1–6 and Extended Data Figs. 1–9. Images and MolecularDocking_Outputs. [file 42255_2026_1524_MOESM8_ESM.zip › ATCase_SourceData/Source Data_ExtDataFig1_Images/143B_-Pyr_Rot_B5_1_00d07h50m.tif]

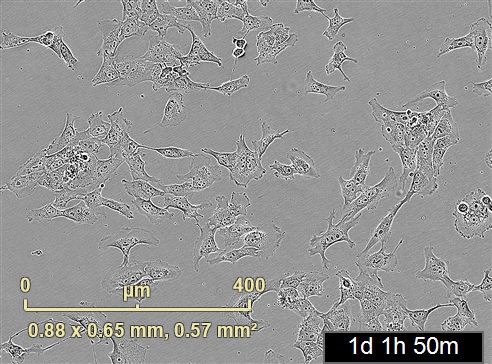

Supplement: Supplementary file 8 — Source data for Figs. 1–6 and Extended Data Figs. 1–9. Images and MolecularDocking_Outputs. [file 42255_2026_1524_MOESM8_ESM.zip › ATCase_SourceData/Source Data_ExtDataFig1_Images/143B_-Pyr_Rot_B5_1_01d01h50m.tif]

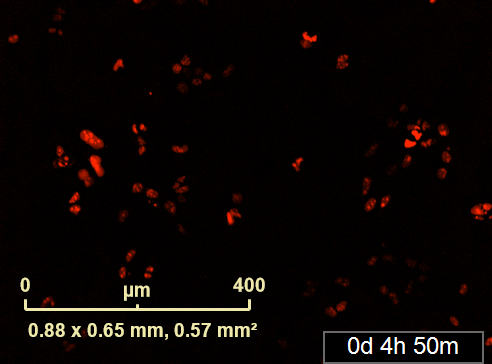

Supplement: Supplementary file 8 — Source data for Figs. 1–6 and Extended Data Figs. 1–9. Images and MolecularDocking_Outputs. [file 42255_2026_1524_MOESM8_ESM.zip › ATCase_SourceData/Source Data_ExtDataFig1_Images/143B_-Pyr_Rot_Red_B5_1_00d04h50m.tif]

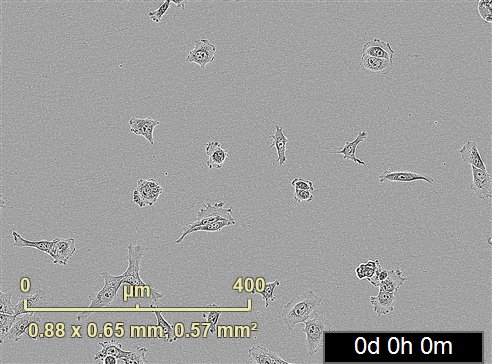

Supplement: Supplementary file 8 — Source data for Figs. 1–6 and Extended Data Figs. 1–9. Images and MolecularDocking_Outputs. [file 42255_2026_1524_MOESM8_ESM.zip › ATCase_SourceData/Source Data_ExtDataFig1_Images/143_+Pyr_Phase_A3_6_00d00h00m.tif]

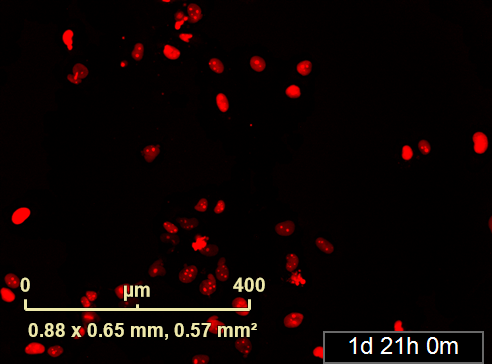

Supplement: Supplementary file 8 — Source data for Figs. 1–6 and Extended Data Figs. 1–9. Images and MolecularDocking_Outputs. [file 42255_2026_1524_MOESM8_ESM.zip › ATCase_SourceData/Source Data_ExtDataFig1_Images/143B_AA5_Red_B2_10_01d21h00m.tif]

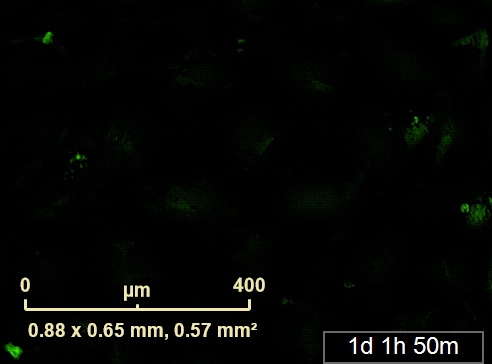

Supplement: Supplementary file 8 — Source data for Figs. 1–6 and Extended Data Figs. 1–9. Images and MolecularDocking_Outputs. [file 42255_2026_1524_MOESM8_ESM.zip › ATCase_SourceData/Source Data_ExtDataFig1_Images/143B_-Pyr_Rot_Green_B5_1_01d01h50m.tif]

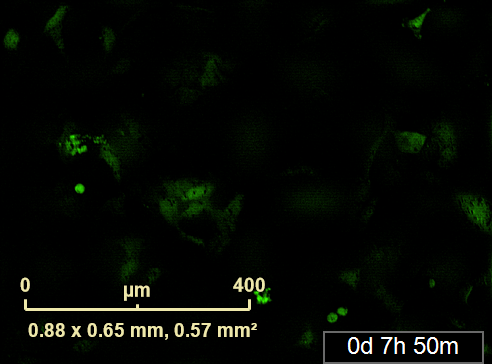

Supplement: Supplementary file 8 — Source data for Figs. 1–6 and Extended Data Figs. 1–9. Images and MolecularDocking_Outputs. [file 42255_2026_1524_MOESM8_ESM.zip › ATCase_SourceData/Source Data_ExtDataFig1_Images/143B_-Pyr_Rot_Green_B5_1_00d07h50m.tif]

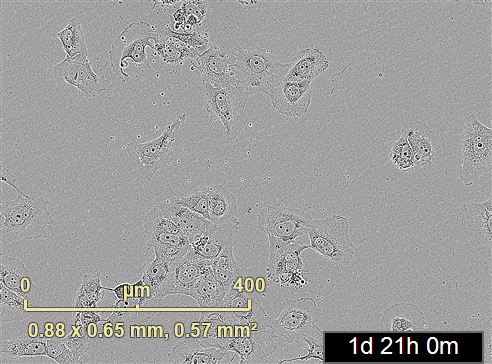

Supplement: Supplementary file 8 — Source data for Figs. 1–6 and Extended Data Figs. 1–9. Images and MolecularDocking_Outputs. [file 42255_2026_1524_MOESM8_ESM.zip › ATCase_SourceData/Source Data_ExtDataFig1_Images/143B_AA5_Phase_B2_10_01d21h00m.tif]

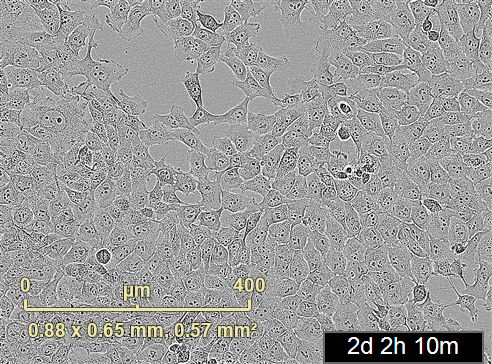

Supplement: Supplementary file 8 — Source data for Figs. 1–6 and Extended Data Figs. 1–9. Images and MolecularDocking_Outputs. [file 42255_2026_1524_MOESM8_ESM.zip › ATCase_SourceData/Source Data_ExtDataFig1_Images/143_+Pyr_Phase_A3_6_02d02h10m.tif]

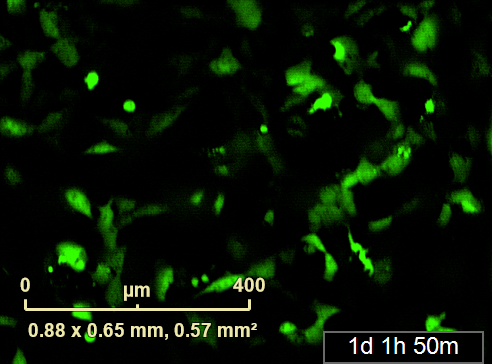

Supplement: Supplementary file 8 — Source data for Figs. 1–6 and Extended Data Figs. 1–9. Images and MolecularDocking_Outputs. [file 42255_2026_1524_MOESM8_ESM.zip › ATCase_SourceData/Source Data_ExtDataFig1_Images/143B_-Pyr_Green_A1_8_01d01h50m.tif]

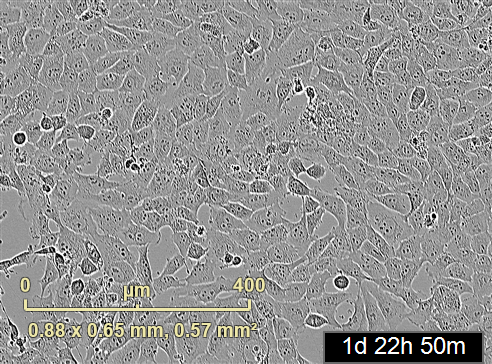

Supplement: Supplementary file 8 — Source data for Figs. 1–6 and Extended Data Figs. 1–9. Images and MolecularDocking_Outputs. [file 42255_2026_1524_MOESM8_ESM.zip › ATCase_SourceData/Source Data_ExtDataFig1_Images/143B_-Pyr_A1_8_01d22h50m.tif]

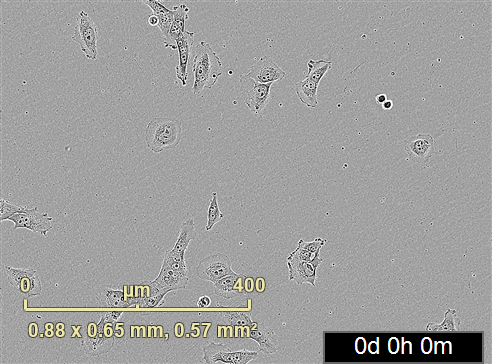

Supplement: Supplementary file 8 — Source data for Figs. 1–6 and Extended Data Figs. 1–9. Images and MolecularDocking_Outputs. [file 42255_2026_1524_MOESM8_ESM.zip › ATCase_SourceData/Source Data_ExtDataFig1_Images/143B_AA5_Phase_B2_10_00d00h00m.tif]

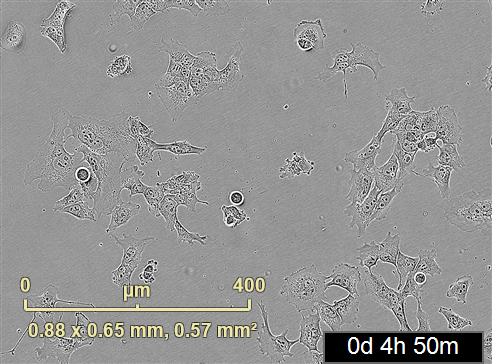

Supplement: Supplementary file 8 — Source data for Figs. 1–6 and Extended Data Figs. 1–9. Images and MolecularDocking_Outputs. [file 42255_2026_1524_MOESM8_ESM.zip › ATCase_SourceData/Source Data_ExtDataFig1_Images/143B_-Pyr_Rot_B5_1_00d04h50m.tif]

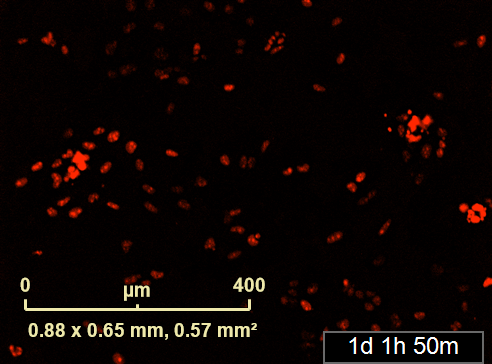

Supplement: Supplementary file 8 — Source data for Figs. 1–6 and Extended Data Figs. 1–9. Images and MolecularDocking_Outputs. [file 42255_2026_1524_MOESM8_ESM.zip › ATCase_SourceData/Source Data_ExtDataFig1_Images/143B_-Pyr_Rot_Red_B5_1_01d01h50m.tif]

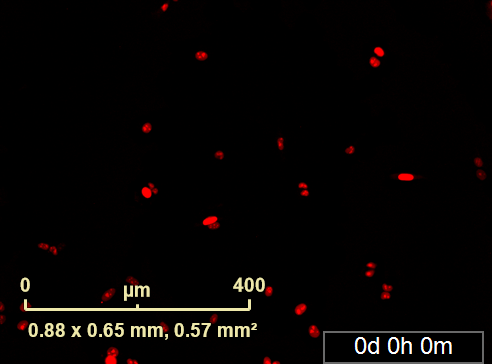

Supplement: Supplementary file 8 — Source data for Figs. 1–6 and Extended Data Figs. 1–9. Images and MolecularDocking_Outputs. [file 42255_2026_1524_MOESM8_ESM.zip › ATCase_SourceData/Source Data_ExtDataFig1_Images/143B_+Pyr_Red_A3_6_00d00h00m.tif]

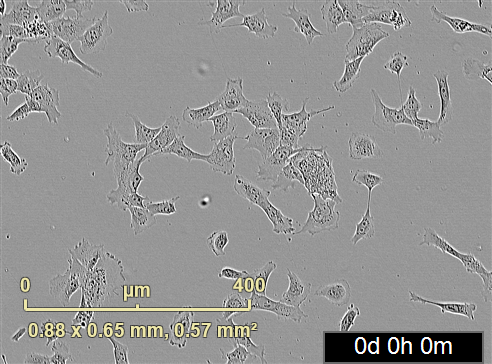

Supplement: Supplementary file 8 — Source data for Figs. 1–6 and Extended Data Figs. 1–9. Images and MolecularDocking_Outputs. [file 42255_2026_1524_MOESM8_ESM.zip › ATCase_SourceData/Source Data_ExtDataFig1_Images/143B_-Pyr_A1_8_00d00h00m.tif]

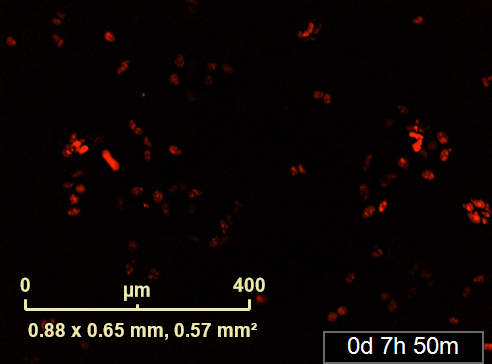

Supplement: Supplementary file 8 — Source data for Figs. 1–6 and Extended Data Figs. 1–9. Images and MolecularDocking_Outputs. [file 42255_2026_1524_MOESM8_ESM.zip › ATCase_SourceData/Source Data_ExtDataFig1_Images/143B_-Pyr_Rot_Red_B5_1_00d07h50m.tif]

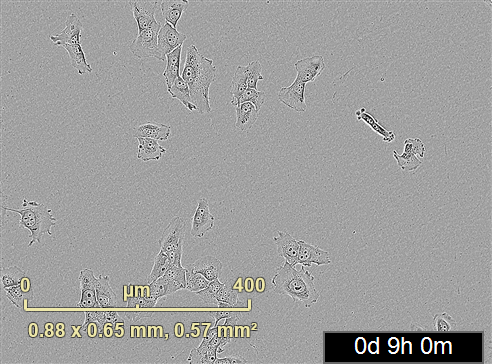

Supplement: Supplementary file 8 — Source data for Figs. 1–6 and Extended Data Figs. 1–9. Images and MolecularDocking_Outputs. [file 42255_2026_1524_MOESM8_ESM.zip › ATCase_SourceData/Source Data_ExtDataFig1_Images/143B_AA5_Phase_B2_10_00d09h00m.tif]

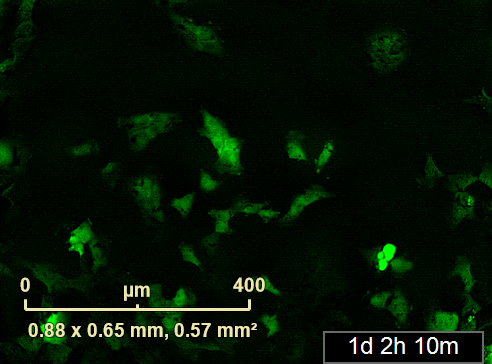

Supplement: Supplementary file 8 — Source data for Figs. 1–6 and Extended Data Figs. 1–9. Images and MolecularDocking_Outputs. [file 42255_2026_1524_MOESM8_ESM.zip › ATCase_SourceData/Source Data_ExtDataFig1_Images/143B_+Pyr_Green_A3_6_01d02h10m.tif]

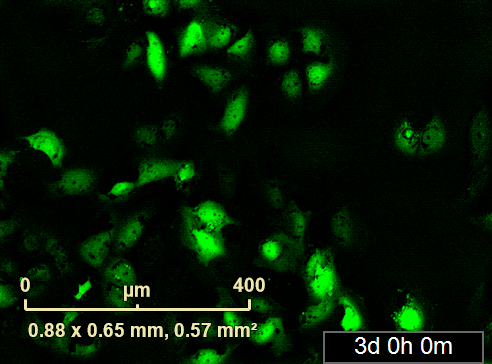

Supplement: Supplementary file 8 — Source data for Figs. 1–6 and Extended Data Figs. 1–9. Images and MolecularDocking_Outputs. [file 42255_2026_1524_MOESM8_ESM.zip › ATCase_SourceData/Source Data_ExtDataFig1_Images/143B_AA5_Green_B2_10_03d00h00m.tif]

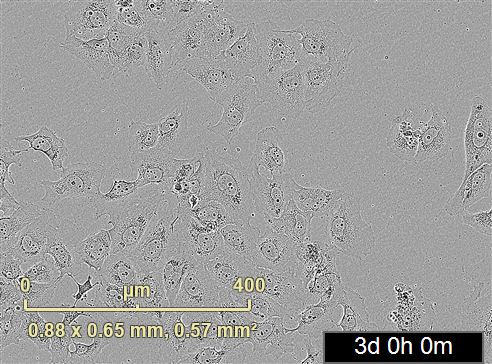

Supplement: Supplementary file 8 — Source data for Figs. 1–6 and Extended Data Figs. 1–9. Images and MolecularDocking_Outputs. [file 42255_2026_1524_MOESM8_ESM.zip › ATCase_SourceData/Source Data_ExtDataFig1_Images/143B_AA5_Phase_B2_10_03d00h00m.tif]

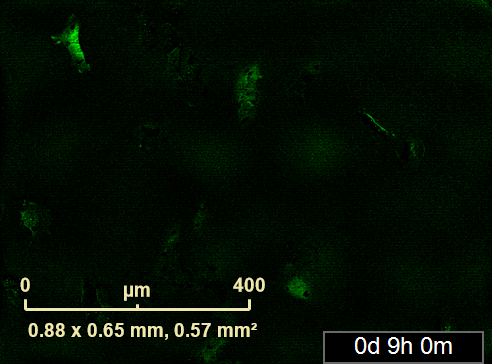

Supplement: Supplementary file 8 — Source data for Figs. 1–6 and Extended Data Figs. 1–9. Images and MolecularDocking_Outputs. [file 42255_2026_1524_MOESM8_ESM.zip › ATCase_SourceData/Source Data_ExtDataFig1_Images/143B_AA5_Green_B2_10_00d09h00m.tif]

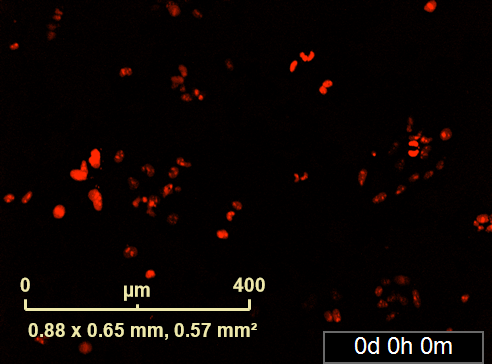

Supplement: Supplementary file 8 — Source data for Figs. 1–6 and Extended Data Figs. 1–9. Images and MolecularDocking_Outputs. [file 42255_2026_1524_MOESM8_ESM.zip › ATCase_SourceData/Source Data_ExtDataFig1_Images/143B_-Pyr_Rot_Red_B5_1_00d00h00m.tif]

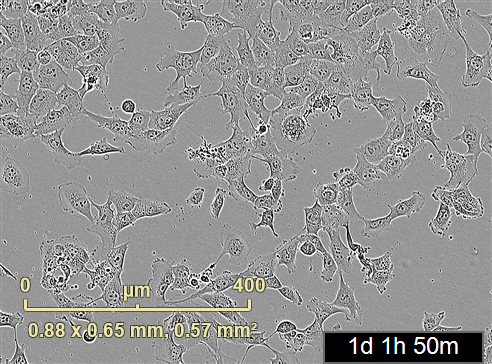

Supplement: Supplementary file 8 — Source data for Figs. 1–6 and Extended Data Figs. 1–9. Images and MolecularDocking_Outputs. [file 42255_2026_1524_MOESM8_ESM.zip › ATCase_SourceData/Source Data_ExtDataFig1_Images/143B_-Pyr_A1_8_01d01h50m.tif]

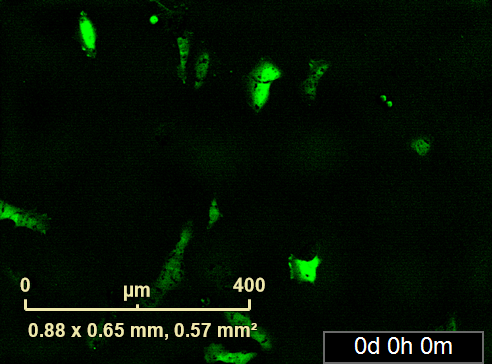

Supplement: Supplementary file 8 — Source data for Figs. 1–6 and Extended Data Figs. 1–9. Images and MolecularDocking_Outputs. [file 42255_2026_1524_MOESM8_ESM.zip › ATCase_SourceData/Source Data_ExtDataFig1_Images/143B_AA5_Green_B2_10_00d00h00m.tif]

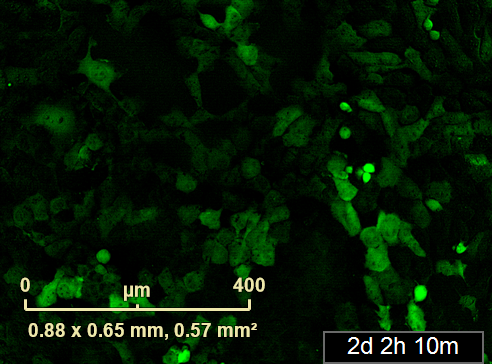

Supplement: Supplementary file 8 — Source data for Figs. 1–6 and Extended Data Figs. 1–9. Images and MolecularDocking_Outputs. [file 42255_2026_1524_MOESM8_ESM.zip › ATCase_SourceData/Source Data_ExtDataFig1_Images/143B_+Pyr_Green_A3_6_02d02h10m.tif]

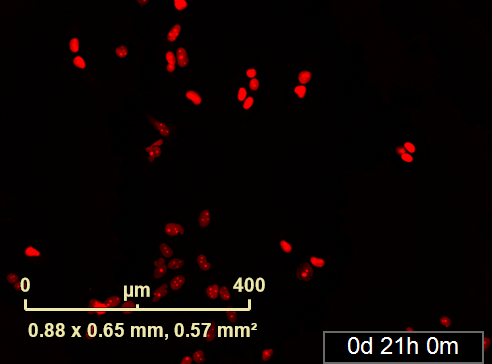

Supplement: Supplementary file 8 — Source data for Figs. 1–6 and Extended Data Figs. 1–9. Images and MolecularDocking_Outputs. [file 42255_2026_1524_MOESM8_ESM.zip › ATCase_SourceData/Source Data_ExtDataFig1_Images/143B_AA5_Red_B2_10_00d21h00m.tif]

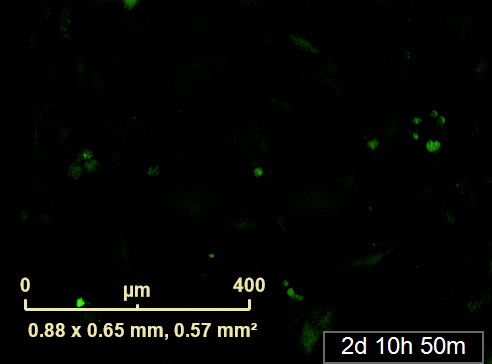

Supplement: Supplementary file 8 — Source data for Figs. 1–6 and Extended Data Figs. 1–9. Images and MolecularDocking_Outputs. [file 42255_2026_1524_MOESM8_ESM.zip › ATCase_SourceData/Source Data_ExtDataFig1_Images/143B_-Pyr_Rot_Green_B5_1_02d10h50m.tif]

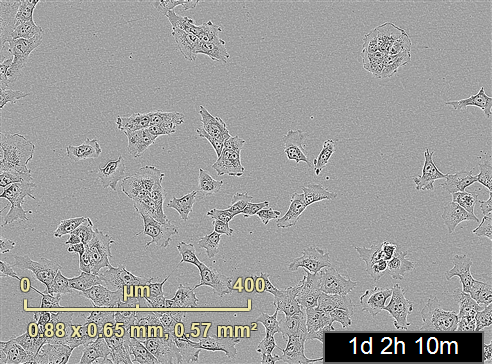

Supplement: Supplementary file 8 — Source data for Figs. 1–6 and Extended Data Figs. 1–9. Images and MolecularDocking_Outputs. [file 42255_2026_1524_MOESM8_ESM.zip › ATCase_SourceData/Source Data_ExtDataFig1_Images/143_+Pyr_Phase_A3_6_01d02h10m.tif]

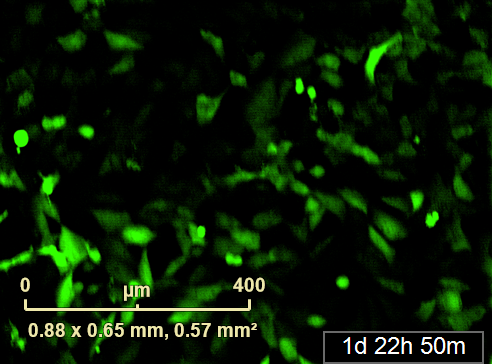

Supplement: Supplementary file 8 — Source data for Figs. 1–6 and Extended Data Figs. 1–9. Images and MolecularDocking_Outputs. [file 42255_2026_1524_MOESM8_ESM.zip › ATCase_SourceData/Source Data_ExtDataFig1_Images/143B_-Pyr_Green_A1_8_01d22h50m.tif]

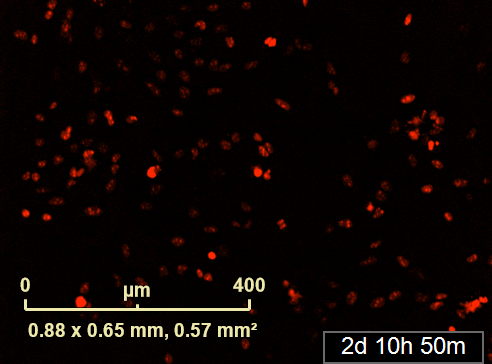

Supplement: Supplementary file 8 — Source data for Figs. 1–6 and Extended Data Figs. 1–9. Images and MolecularDocking_Outputs. [file 42255_2026_1524_MOESM8_ESM.zip › ATCase_SourceData/Source Data_ExtDataFig1_Images/143B_-Pyr_Rot_Red_B5_1_02d10h50m.tif]

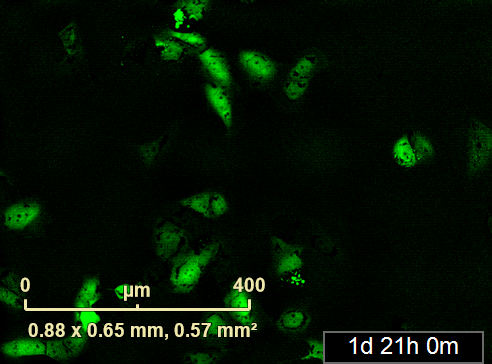

Supplement: Supplementary file 8 — Source data for Figs. 1–6 and Extended Data Figs. 1–9. Images and MolecularDocking_Outputs. [file 42255_2026_1524_MOESM8_ESM.zip › ATCase_SourceData/Source Data_ExtDataFig1_Images/143B_AA5_Green_B2_10_01d21h00m.tif]

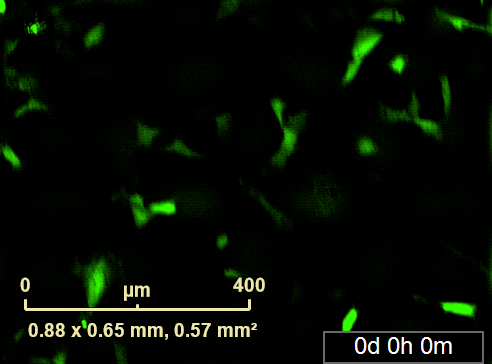

Supplement: Supplementary file 8 — Source data for Figs. 1–6 and Extended Data Figs. 1–9. Images and MolecularDocking_Outputs. [file 42255_2026_1524_MOESM8_ESM.zip › ATCase_SourceData/Source Data_ExtDataFig1_Images/143B_-Pyr_Green_A1_8_00d00h00m.tif]

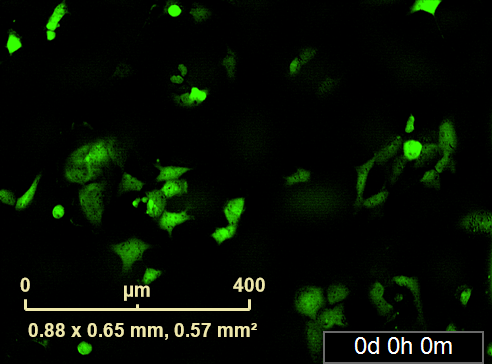

Supplement: Supplementary file 8 — Source data for Figs. 1–6 and Extended Data Figs. 1–9. Images and MolecularDocking_Outputs. [file 42255_2026_1524_MOESM8_ESM.zip › ATCase_SourceData/Source Data_ExtDataFig1_Images/143B_-Pyr_Rot_Green_B5_1_00d00h00m.tif]

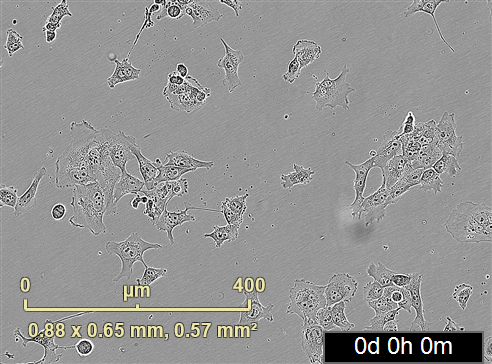

Supplement: Supplementary file 8 — Source data for Figs. 1–6 and Extended Data Figs. 1–9. Images and MolecularDocking_Outputs. [file 42255_2026_1524_MOESM8_ESM.zip › ATCase_SourceData/Source Data_ExtDataFig1_Images/143B_-Pyr_Rot_B5_1_00d00h00m.tif]

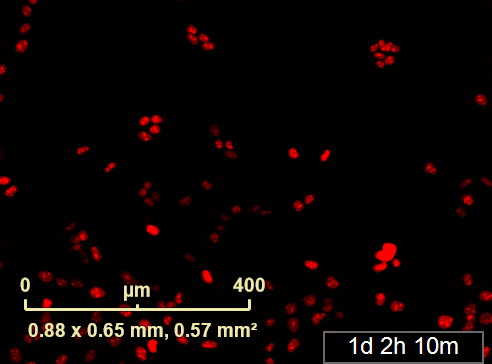

Supplement: Supplementary file 8 — Source data for Figs. 1–6 and Extended Data Figs. 1–9. Images and MolecularDocking_Outputs. [file 42255_2026_1524_MOESM8_ESM.zip › ATCase_SourceData/Source Data_ExtDataFig1_Images/143B_+Pyr_Red_A3_6_01d02h10m.tif]

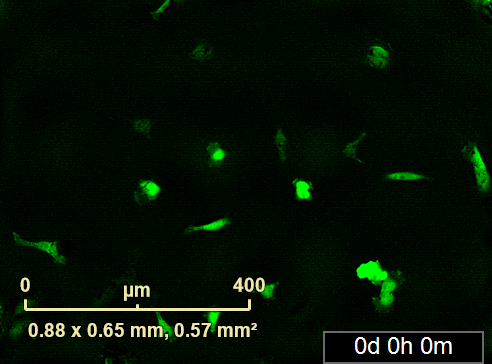

Supplement: Supplementary file 8 — Source data for Figs. 1–6 and Extended Data Figs. 1–9. Images and MolecularDocking_Outputs. [file 42255_2026_1524_MOESM8_ESM.zip › ATCase_SourceData/Source Data_ExtDataFig1_Images/143B_+Pyr_Green_A3_6_00d00h00m.tif]

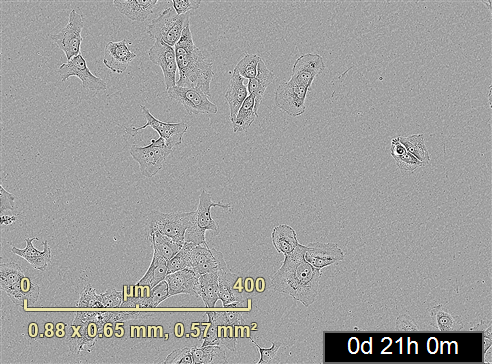

Supplement: Supplementary file 8 — Source data for Figs. 1–6 and Extended Data Figs. 1–9. Images and MolecularDocking_Outputs. [file 42255_2026_1524_MOESM8_ESM.zip › ATCase_SourceData/Source Data_ExtDataFig1_Images/143B_AA5_Phase_B2_10_00d21h00m.tif]

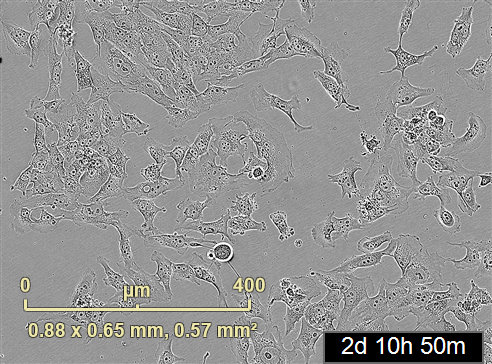

Supplement: Supplementary file 8 — Source data for Figs. 1–6 and Extended Data Figs. 1–9. Images and MolecularDocking_Outputs. [file 42255_2026_1524_MOESM8_ESM.zip › ATCase_SourceData/Source Data_ExtDataFig1_Images/143B_-Pyr_Rot_B5_1_02d10h50m.tif]

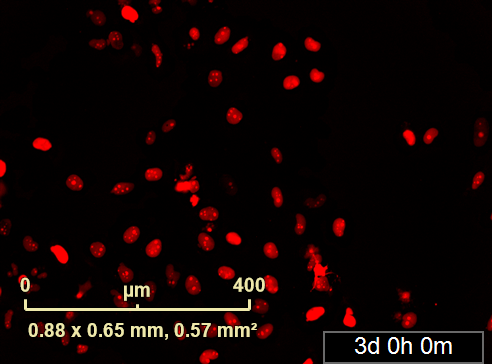

Supplement: Supplementary file 8 — Source data for Figs. 1–6 and Extended Data Figs. 1–9. Images and MolecularDocking_Outputs. [file 42255_2026_1524_MOESM8_ESM.zip › ATCase_SourceData/Source Data_ExtDataFig1_Images/143B_AA5_Red_B2_10_03d00h00m.tif]

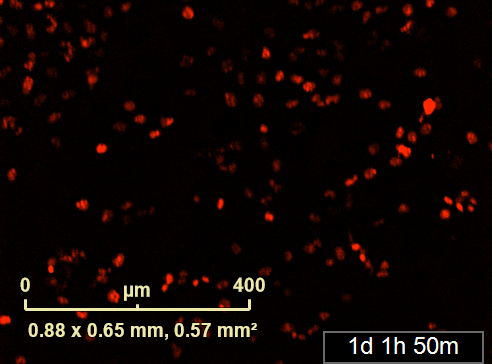

Supplement: Supplementary file 8 — Source data for Figs. 1–6 and Extended Data Figs. 1–9. Images and MolecularDocking_Outputs. [file 42255_2026_1524_MOESM8_ESM.zip › ATCase_SourceData/Source Data_ExtDataFig1_Images/143B_-Pyr_Red_A1_8_01d01h50m.tif]
